# The assessment of the usability of selected instrumental techniques for the elemental analysis of biomedical samples

**DOI:** 10.1038/s41598-021-82179-3

**Published:** 2021-02-12

**Authors:** Karolina Planeta, Aldona Kubala-Kukus, Agnieszka Drozdz, Katarzyna Matusiak, Zuzanna Setkowicz, Joanna Chwiej

**Affiliations:** 1 Faculty of Physics and Applied Computer Science, AGH University of Science and Technology, Krakow, Poland; 2 Institute of Physics, Jan Kochanowski University, Kielce, Poland; 3 Holly Cross Cancer Centre, Kielce, Poland; 4 Institute of Zoology and Biomedical Research, Jagiellonian University, Krakow, Poland

**Keywords:** Biological physics, Techniques and instrumentation, Biomarkers, Analytical chemistry, Chemical biology, Medicinal chemistry

## Abstract

The fundamental role of major, minor and trace elements in different physiological and pathological processes occurring in living organism makes that elemental analysis of biomedical samples becomes more and more popular issue. The most often used tools for analysis of the elemental composition of biological samples include Flame and Graphite Furnace Atomic Absorption Spectroscopy (F-AAS and GF-AAS), Inductively Coupled Plasma Optical Emission Spectroscopy (ICP-OES) and Inductively Coupled Plasma Mass Spectrometry (ICP-MS). Each of these techniques has many advantages and limitations that should be considered in the first stage of planning the measurement procedure. Their reliability can be checked in the validation process and the precision, trueness and detection limits of elements belong to the most frequently determined validation parameters. The main purpose of this paper was the discussion of selected instrumental techniques (F-AAS, GF-AAS, ICP-OES and ICP-MS) in term of the achieved validation parameters and the usefulness in the analysis of biological samples. The focus in the detailed literature studies was also put on the methods of preparation of the biomedical samples. What is more based on the own data the usefulness of the total reflection X-ray fluorescence spectroscopy for the elemental analysis of animal tissues was examined. The detection limits of elements, precision and trueness for the technique were determined and compared with the literature data concerning other of the discussed techniques of elemental analysis. Reassuming, the following paper is to serve as a guide and comprehensive source of information concerning the validation parameters achievable in different instrumental techniques used for the elemental analysis of biomedical samples.

## Introduction

Instrumental techniques are a group of research tools applied for investigation of analytes in various types of matter. These include i.a. spectroscopic techniques and mass spectrometry. Spectroscopic techniques are based on the generation and interpretation of the atomic spectra obtained as a result of the interaction of electromagnetic radiation with the analyte. These interactions involve physical phenomena such as absorption or emission and depending on the type of interaction, spectroscopic techniques are distinguished^1^. Mass spectrometry is based on the ionization of atoms contained in the analysed sample and separation of the formed charged particles based on their mass to charge ratio^2^. Both atomic and mass spectra allow obtaining qualitative and quantitative information on the composition of the analyte. Atomic absorption spectroscopy (AAS), inductively coupled plasma optical emission spectroscopy (ICP-OES) and inductively coupled plasma mass spectrometry (ICP-MS) are widely used in the studies on the elemental composition of samples of various origin, including biomedical samples^3–9^.

An important issue related to the reliability of the results of analyses is their validation. It is a process of determining the values of parameters characterizing the efficiency of operation and the suitability of a given technique for the research purposes set. Validation is carried out to ensure that the analysis process is fair and precise. Demonstrating the reliability of the results obtained in this way is very important both for their correct interpretation and for the possibility of their future use by other research groups. In the literature, the most frequently appearing validation parameters for quantitative elemental analyses are the accuracy and precision of the measurement as well as the detection limit of the determined elements^10–13^.

The biomedical samples can be divided into three main categories. The first category are liquid samples, which include blood and its liquid components^14^, urine^15^, cerebrospinal^16^ or amniotic fluid^17^. There is also a group of soft tissues, which are mainly organ or skin samples^18–22^. The third category includes hard tissues, i.e. hair^23^, nails^24^, kidney stones^25^, teeth^26^ and bones^12^. These samples can be of both human and animal origin, and the latter especially in the case of in vivo experiments^27–31^. The elements that they contain perform many important functions, and maintaining their content at the appropriate levels is crucial for the proper functioning of the living organism^32–35^. What is more, the changes in their tissue concentrations may reflect physiological and pathological processes occurring in the body^36–39^. Besides the analysis of essential elements, their physiological concentration and changes occurring during different pathological states, the investigations are focused on the evaluation of the content of toxic elements in case of poisoning or environmental exposure^7,8,40–42^.

Elemental analysis of biological samples is a difficult issue, mainly, due to their complex composition and to the low levels of the elements^43,44^. Therefore, among different factors that should be taken into account when choosing an analytical tool, the most important are: minimum detectable element concentrations, the number of elements one want to determine, the expected accuracy and precision of measurements or the available amount of research material^45–48^. Awareness of the analytical capabilities of a given technique is a key element to correctly plan the measurement procedure.

The following work was divided into two parts. The purpose of the first one was to discuss the use of AAS, ICP-OES and ICP-MS in the quantitative elemental analysis of biomedical samples. The values of accuracy, precision and detection limits of elements for various techniques were compared and discussed. What is more, the summary concerning the methods of preparation of biomedical samples was presented. The second part of the work was the evaluation of the usefulness of the total reflection X-ray fluorescence (TXRF) technique for elemental analysis of tissue samples. The TXRF, due to its analytical capabilities and sample preparation requirements, is similar to the discussed instrumental techniques of elemental analysis and therefore was chosen from the collection of methods based on X-ray fluorescence for comparison with other presented techniques. The principles of the TXRF and examples of its use for analysis of biomedical samples were discussed. The validation parameters for the technique were determined and compared with the values achieved for other of the discussed analytical techniques.

## Selected instrumental techniques and their use for elemental analysis of biomedical samples

### Atomic absorption spectroscopy (AAS)

**Figure 1:**
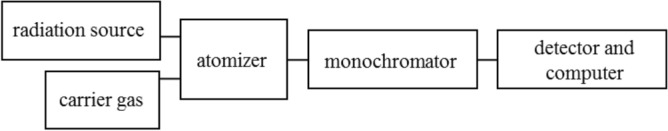
Block diagram of a typical instrument used for atomic absorption spectroscopy.

Atomic absorption spectroscopy is one of the most commonly used technique in analytical laboratories. Due to its simplicity and low operating costs, AAS in widely applied in single-elemental analysis. The basic principle of the AAS technique is that energy-characteristic radiation is selectively absorbed by free atoms of elements. The number of free atoms in the absorbing medium is proportional to the concentration of the element in the analysed sample. The absorbance, measured in the AAS technique, depends on the number of free atoms and thus on their concentration in the sample. This dependence is the basis for quantitative analyses carried out by AAS. Depending on the atomization method, two types of AAS are distinguished: flame (F-AAS) and graphite-furnace (GF-AAS). In the F-AAS, sample in an aerosol form is introduced into a burning flame, while in GF-AAS sample is transferred into a special cuvette, heated and gradually evaporated^49,50^. The block diagram of the apparatus used in the AAS technique is shown in the Fig. 1.

GF-AAS is based on the use of flameless atomizer, usually graphite cuvette to which sample is introduced and gradually heated. Increasing temperature results in drying the sample, removal of its matrix and atomization. Before measurement, substances reducing the volatility of the analysed element are added to the sample. These substances are called modifiers and their used make easier to separate analysed element from the matrix. Sometimes, interactions between free atoms and atomizer occur and it is the main disadvantage of this technique^49^. Xu et al. analysed the concentration of Al in human brain tissue. Due to serious interferences occurring between Al and P, which content is high in this type of samples, potassium dichromate as a chemical modifier was applied. It resulted in a decrease of elemental disturbances and improvement of Al determination^51^. Dudek-Adamska et al. investigated how the addition of chemical modifier influences the determination of Ni in human organ samples. For this purpose, magnesium nitrate, palladium nitrate and mixture of magnesium nitrate and ammonium dihydrogen phosphate were tested as chemical modifiers. Based on the performed validation tests they found that the determination of Ni was the best without any matrix modifiers and at temperatures 1300 °C and 2400 °C for pyrolysis and atomization, respectively^19^. A similar study performed for Cr showed better accuracy when magnesium nitrate was used as chemical modifier at temperatures of pyrolysis equals 1400 °C and atomization—2500 °C^52^. Application of palladium or magnesium nitrate as chemical modifiers for Al and Mn determination in human hair samples decreased the influence of background on the obtained results^53^. In turn, in case of Al analysis in human bone samples, there was no need to use an additional modifier because its function was fulfilled by Ca present at high concentration in bones^12^.

The type of used flame in F-AAS measurements depends on the elements under analysis. Goldberg et al. determined physiological concentrations of Cu, Mn, Fe and Ca in six regions of human brain. To analyse Cu and Mn, they used graphite furnace, while for Fe and Ca, air-acetylene and nitrous oxide-acetylene flames, respectively^54^. Flame AAS was used to determine the concentration of potentially toxic elements (Fe, Mg, Ca, Cu, Zn, Cr, Cd and Pb) in human hair samples. The technique was not useful for Cd and Pb determination due to the fact that results obtained for these elements were below the detection limits^34^. By using the GF-AAS technique human breast samples were analysed by Leung et al. The authors investigated the differences in Si concentration between tissues taken from women with and without silicone implants^55^. In cancerous breast tissue concentrations of Cd, Pb^56^ and Al^57^ were investigated. Villeneuve et al. determined levels of Fe in liver needle-biopsy samples taken from patients who suffered from cirrhosis and they assessed the variability of Fe content depending on the region of tissue origin^58^. Using the GF-AAS the possible exposure of steel industry workers on trace and toxic elements, produced in manufactures processes, was investigated. Pb, Cd, Ni, Cr^59^ as well as As, Cu, Co and Mn^60^ concentrations were determined in their blood, urine and hair samples. Campillo et al. developed a fast method for Mo, Cr and Al determination in human urine by GF-AAS. The procedure did not require the sample pre-treatment and the background signal was reduced by addition of hydrogen pyroxide and nitric acid to the sample^15^. Khlifi et al. analysed the content of As, Cd, Cr and Ni in healthy and cancerous tissue from patients suffering from head and neck cancers^61^.

Various methods are used for improving the sensitivity of measurements with AAS. Yaman et al. analyzed differences in concentrations of Cd, Cu, Zn, Fe, Mg, Ca and Ni between the cancerous and non-cancerous tissues taken from ovary and endometrium. For better sensitivity of Cd and Cu determination, they used the slotted tube atom trap (STAT)^62^. This device significantly increased the time of residence of the free atoms in the measurement area and allowed to obtain lower detection limits comparing to conventional F-AAS^50^. A very common procedure, leading to sample preconcentration and allowing the determination of elements at the ultra-trace levels, is cloud point extraction (CPE). This method involves adding to the sample some chemical compounds, mostly nonionic surfactants, which at high temperature forms a separate phase in the sample solution. The analyte, found in the acid solution of the sample previously subjected to digestion, concentrates together with the surfactant in a small volume of new established phase and thus it is separated from the sample. The surfactant-rich phase containing an analyte is then subjected to further analysis. CPE was used, among others, by Arain et al. for measurements of Ni content in blood and serum of patients who suffered from oropharyngeal cancer^63^. In turn, Shemirani et al. applied CPE for sample preconcentration analysing the content of Bi in human urine and hair^44^. Dual-cloud point extraction (d-CPE) used for the preconcentration of hair samples before Mn determination by F-AAS allowed to improve the recovery value from 97.1% (without preconcentration) to 99.2% (d-CPE)^64^. Another procedure used for sample preconcentration is solid-phase extraction. Baghban et al. applied it for human hair and nail samples before Cd and Pb determination by F-ASA. To verify the obtained results also GF-AAS measurements were performed^24^.

In some studies, different techniques are chosen for the analysis of individual element. Jablonska et al. determined Cd, As, Se and Fe concentrations in the tissue of human breast cancer trying to indicate whether there are relationships between their contents. For Cd and Se, GF-AAS was used with palladium and palladium-magnesium matrix modifier, respectively. Flame AAS was used for Fe determination whilst As concentration was measured using ICP-MS with kinetic energy discrimination chamber, which allowed for the decrease of the influence of polyatomic interferences^65^.

The elements such as As, Sb, Pb, Bi, Te, Se and Sn have a properties to form, with a hydrogen, volatile hydrides. This reaction coupled with an atomic absorption spectroscopy constitutes the basis of hydride generation atomic absorption spectroscopy (HG-AAS). Volatile hydrides of elements, which are formed by adding a reducing agent to the sample, are directed to the atomizer in the stream of carrier gas. As the element is isolated from the sample, matrix effects are reduced, what results in the improvement of detection limits. A technique basing on similar operating principles which allows for the determination of mercury, is called the Cold vapour atomic absorption spectroscopy (CV-AAS). Lech et al. used ICP-OES and CV-AAS for Hg determination in human organs and blood. CV-AAS is one of the primary tools applied for mercury determination. Due to the fact that this element might occur as the free atoms at room temperature, application of furnace is not necessary. As a result of adding a reducing agent to acid environment of analysed sample solution, the mercury ions contained in sample are reduced to its elemental form. Then they are transported in the stream of carrier gas to the measuring chamber. In this case, CV-AAS turned out to be a more sensitive technique of Hg determination in blood, characterized with the lowest detection limit of the element^46^.

### Inductively coupled plasma optical emission spectroscopy (ICP-OES)

Inductively coupled plasma optical emission spectroscopy is currently one of the most sensitive and precise technique of instrumental analysis. It is based on the measurement of radiation emitted by atoms (in the gaseous form) passing from excited to the lower energy state. Plasma, electrically neutral gas consisting of free electrons and positive ions, is the source of atoms excitations and it is generated by inductively coupled system. The resulting emission spectrum is linear, i.e. it consist of series of lines corresponding to specific wavelengths of radiation emitted by atom during transition between energy levels. The arrangement of emission lines is unique for particular element, therefore it is possible to identify it and determine its content in the sample.

**Figure 2:**
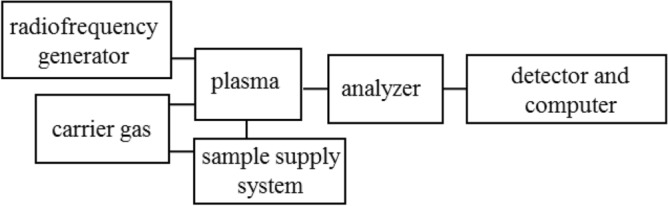
Block diagram of a typical instrument used for ICP-OES.

ICP-OES apparatus consists of a sample supply system, plasma generation system, analyzer, detector and computer. Its block diagram is presented in Fig. 2. The sample supply system is usually based on nebulization or thermal evaporation. In the first case, the sample in the form of solution is dispersed into the carrier gas stream and form an aerosol. In thermal evaporation, sample is heated which results in its evaporation and introduction into plasma with gas^66^. The technique of generating volatile hydrides is also used^67^. Radiation emitted by excited atoms goes to the analyzer, usually monochromators or polychromators and then it is registered by detector (photomultipliers or multi-channel spectrometers).

ICP-OES was used to determine physiological concentrations of 13 trace elements (Al, B, Ba, Cd, Co, Cr, Cu, Fe, Mn, Ni, Pb, Se, Sr and Zn) in human autopsy samples taken from cerebellum, heart, kidneys, liver, pancreas, spleen and ovary^3^. Other research aimed to find if there are any correlations between concentrations of different trace elements (Cu, Co, Cr, Fe, Mn, Ni, Se, Zn, Al, Ba, Cd, Pb, Sr) within particular organ and between different organs collected during autopsy. Such information may contribute to better understanding of interactions between trace elements and their distribution in human organs^20^. Chen et al. applied electrothermal vaporization as a sample supply method for determination of Ti, Cu, Cr, Fe, Zn and Ca in human hair and serum. The use of thermal evaporation minimizes matrix effects, allows to measure small amounts of sample and limits the need of its chemical pre-treatment^47^. ICP-OES was one of the techniques used to analyze organ samples taken from a person suspected to mercury poisoning. Blood, stomach, liver and kidney were examined for the content of this element. The technique proved to be useless in case of blood tests, where the Hg level was below the detection limit^46^. Tohno et al. analysed concentrations of Ca, P and Mg in various types of human arteries. It was investigated whether there are dependencies between the contents of particular elements and whether the obtained elemental levels correlate with the age of examined patients^68^. Similar study was carried out by Yang et al. They determined the content of Ca, Mg and Fe in rabbit arteries and developed analytical procedure allowing determination of the elemental composition for very small amount of the sample of biological origin^69^. Naganuma et al. determined the content of S, Mg, Ca, P, Zn, Fe and Al in human round ligaments. They analyzed the elemental changes occurring in tissues with age and the relationships between the contents of particular elements^70^. ICP-OES was one of the techniques used to examine the content of heavy metals (Cu, Fe, Mn, Zn, Cd, Pb) in human brain. This technique, however, did not prove to be useful in assessing of the Pb content, which was lower than the achieved detection limit^45^. Andrasi et al. used the ICP-OES to determine Al, Mg, P and Al, Zn, Cu, Mn and Fe levels in different regions of human brain. They verified differences in concentrations of these elements in samples taken from healthy people and those suffering from Alzheimer's disease^71,72^ and analysed the concentrations of Na, K, Mg, Fe, Cu, Mn Zn P and S in normal human brain^73^. The other research concerned the determination of the content of Ca, P, S, Fe, Mg, Zn and Cu in 28 regions of the brain taken from patient suffered from Wilson's disease^74^. The differences in the content of 21 elements (Ag, Al, As, Ba, Ca, Cd, Co, Cr, Cu, Fe, Mg, Mn, Na, Ni, Pb, Sb, Se, Sr, Tl, V and Zn) between healthy liver, breast and lungs and tissues taken from the tumor-altered organs were also examined^75^. ICP-OES was also used by Mohammadi et al. to determine the concentration of Se in human breast cancer tissues^56^.

Using the ICP-OES MacLachlan et al. determined the contents of Cu and Zn in liver, kidney and muscle of Australian sheep^76^. The concentrations of Ba, Cd, Co, Cr, Cu, Fe, Hg, Li, Mn, Mo, Ni, Pb, Sr and Zn in wolf liver samples were also evaluated^77^. Due to the extensive use of rats in laboratory experiments, their organs are often the subject of elemental analysis, also using the ICP-OES. Leblondel et al. measured concentrations of 14 elements (Na, K, Ca, Mg, S, P, Fe, Sr, Mn, Cu, Zn, Mo and Ba) in whole blood, plasma, liver, kidney, brain, heart, spleen, skeletal muscle, thymus and bone of rat^78^. In turn, Shapira et al. analyzed whether ketamine affects Ca and Mg concentrations in the brain of head trauma rats^27^. The effect of furosemide on Cd, Cu, Fe, Mg, Pb, Se and Zn contents in rat liver, kidney, lung and serum was also investigated^79^. The ICP-OES was also used in the assessment of the impact of arsenic, administered orally to rats, on the concentration of Cu, Zn and Mn in the liver and kidney^21^.

ICP-OES is often used along with the ICP-MS. Using the first technique, the contents of the major elements are determined, whilst ICP-MS is used for trace elements determination. Using such a solution, Takahashi et al. determined the content of P, K, Na, Fe, Mg and Ca in the liver of the Wistar rat^13^. Similarly, concentrations of the major elements (Na, Mg, Si, P, K, Ca, Fe and Zn) in the rat kidney were determined by Shimamura et al.^28^ whilst Sivrikaya et al. analysed the influence of Zn supplementation on Pb, Co, Mo, Cr, B, Mg, Fe, Cu, Ca, Zn and Se distribution in this organ^80^. In the literature one can find also information concerning the use of the ICP-OES in the analysis of liquid clinical samples. Korvela et al. checked whether there are differences in the concentration of Ca, Mg, P, K and Na in the cerebrospinal fluid of people suffering from neuropathic pain, subjected to spinal cord stimulation^16^. Cerebrospinal fluid, serum, blood and urine were the subject of investigation of Forte et al. who examined whether there are differences in concentrations of Ca, Cu, Fe, Mg, Si and Zn between the samples of biological fluids taken from healthy people and those suffering from Parkinson's disease^81^. Human blood and plasma were also analysed by Harrington et al. in terms of Ca, Fe, K, Mg and Na contents^32^ whilst Chen et al. used electrothermal vaporization to analyse the human serum and determine the content of Ti, Cu, Cr, Fe, Zn and Ca. The applied technique of sample introduction allowed for direct analysis of samples with low mass and contributed to the improvement of the detection limits of elements and minimized the matrix effect^47^. Bianchi et al. have undertaken to optimize the ICP-OES measurement procedure aiming at the determination of the content of Li, Na, K, Al, Fe, Mn and Zn in the human serum^82^ whilst Rahil-Khazen et al. carried out validation of the procedure of trace elements analysis (Al, B, Ba, Be, Cd, Co, Cr, Cu, Fe, Li, Mn, Ni, Pb, Se, Sr and Zn) in such samples. It was found that ICP-OES cannot be used as a routine technique of human plasma analysis in case of Al, Be, Co, Cd Cr, Ni and Pb as the concentrations of these elements are below the detection or quantification limits. However, the analysis of the mentioned elements could be possible when their concentrations would increase due to the toxication. The other way to get an information about concentrations of these elements, presented by authors, is application of standard addition method. In this method, increasing amounts of analyte are adding to the sample and the signal intensity for a given analyte concentration is measured. Then, a regression line is created and by extrapolation, knowing the signal intensity in the original sample, one can read the element concentration^83^. Hasegawa et al. examined the elemental composition of bone-marrow fluid. Using the ICP-OES, they determined concentrations of Na, K, Fe, P, Ca, Mg, Al and Zn in the samples^35^.

The ICP-OES is also used for the elemental analysis of hard biological samples. Sahuquillo et al. determined concentrations of Cu, Fe, Mn and Zn in human gallstones previously prepared using the focused-microwave wet digestion method. They analysed the impact of Ca on the determination of other elements. They found that high Ca level in samples influenced the measured Fe and Zn concentrations and in order to reduce this effect they recommended to prepare a calibration solution with a concentration of Ca similar to those of the sample^25^. ICP-OES has also been used to determine the content of Al, B, Ba, Ca, Cu, Fe, K, Li, Mg, Mn, Na, P, S, Sr, V and Zn in human bone of the ribs. It was checked whether there are dependencies between the levels of elements and the age and sex of people from whom samples were taken^84^. Animal bones (domestic dog) were analysed for Zn, Cu, Pb, Cd and Hg content^85^. Chew et al. used the ICP-OES to analyse Zn, Pb and Cu concentrations in human teeth^26^. The discussed technique was also used to investigate the level of selected elements in human hair^47,86^.

### Inductively coupled plasma mass spectrometry (ICP-MS)

Inductively coupled plasma mass spectrometry is an elemental analysis technique that derives from the ICP-OES. ICP is used here to ionize the atoms of the sample which are afterwards separated and identified based on their mass-to-charge ratio. The composition of ions in plasma is proportional to their concentration in the original sample solution. ICP-MS allows for precise identification as well as quantitative multi-elemental analysis and what is more, it makes possible to measure particular isotopes of the analyzed element. Another important feature of the ICP-MS is the ability to detect and measure elements occurring in the sample in very low concentrations. Therefore, it significantly exceeds the capabilities of other techniques of elemental analysis. Also, analysis of non-metals can be carried out with very good sensitivity. Additional advantages, such as high accuracy and precision of measurements as well as minimal disturbances, make ICP-MS one of the most important and useful technique of trace analysis of biomedical samples^2^.

**Figure 3:**
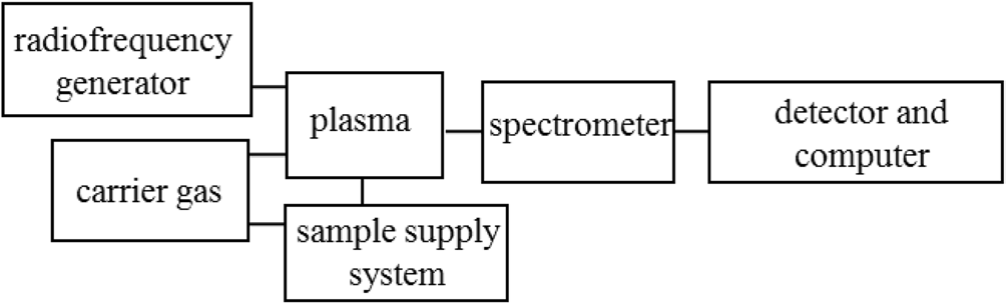
Block diagram of a typical instrument used for ICP-MS.

The basis of analysis using mass spectrometry is to obtain the mass spectra of the analysed sample showing the distribution of ions (or other charged particles) as a function of their mass-to-charge ratio. The apparatus for ICP-MS measurements consists of an ICP forming module, a sample introduction system, a mass spectrometer with an ion detector and data acquisition system. It is presented in Fig. 3.

ICP-MS was applied for multielemental (Al, As, Ba, Ca, Cd, Co, Cr, Cu, Mg, Mn, Ni, Pb, Sb, Se, Sr, U, V and Zn) analysis of amniotic fluid[17]. It was also used to determine the concentrations of Rb, Cu, Se, Ba, Sr, Zr, Cs, Sb, Sn, Mo, Ag and W in human bone-marrow fluid samples^35^. Korvela et al. analysed Ti, As, Rb, Sr and Ba contents in cerebrospinal fluid of patients with neuropathic pain, who were subjected to spinal cord stimulation^16^. Cerebrospinal fluid as well as blood, serum and urine were the subject of investigation of Forte et al., who determined Al and Mn concentrations in these samples. The purpose of their study was the comparison of elemental composition of samples taken from healthy people and patients diagnosed with Parkinson disease^81^. ICP-MS was also used by Harrington et al. for determination of mineral elements (Na, Ca, Mg, K, Fe, Zn, Cu and Se) content in human blood and serum samples. The authors presented the methodological approach for the analysis of small sample volumes (about 250 µl) and the obtained results were with agreement with the literature data^32^. The concentration of Sb was examined in blood, serum, urine and hair of patients with parasitic disease^87^. The technique was also used for determination of 37 trace elements in more than a hundred human blood samples and the obtained results are to help toxicologist in the assessment of health effects caused by possible environmental exposure to metals^88^. ICP-MS, with high resolution magnetic sector, was applied for determination of physiological concentrations of 16 trace elements in children plasma. For most samples, Cd, Pb, V, Cr and As contents were very low, even below the calculated limits of quantification^89^. Human plasma was also the subject of the study carried out by Meyer et al., who used ICP-MS for analysis of significant (Mg, Ca, Fe, Cu, Zn, Mo, Se, I) and toxic (As and Cd) elements^90^. Heitland et al. studied the case of child poisoned with hexavalent chromium and inorganic arsenic. They used ICP-MS to determine a concentration of Cr (VI) in erythrocytes and total Cr and As content in blood, plasma, urine and liver tissue taken from the child. Using high-performance liquid chromatography (HPLC) that allowed to separate the inorganic species of As from sample, they also managed to determine its concentration in urine. The addition of ethanol to urine before measurement reduced non-spectral interferences caused by the presence of carbon. Additionally, this procedure increased the efficiency of nebulization and therefore gave the higher sensitivity of the As determination^8^. The ICP-MS analysis of trace elements in urine was carried out to designate the differences in their concentrations between adults and children^91^. ICP-MS is also used to determine particular isotopes of elements in a sample. Among others, abundances of Fe isotopes in human blood^92^ and uranium in human urine samples were analysed^93^.

ICP-MS was used for Fe, Cu, Mg, Mn, Ca and Zn analysis in samples taken from 13 regions of human brain. The correlations between elemental composition and age as well as inter-hemispherical differences were investigated^9^. ICP-MS was also applied for quantitative analysis of gadolinium in different regions of the brain taken post-mortem from patients in which Gd was used as a contrast agent during MRI examination^94^. Panayi et al. determined concentrations of Cd and Zn in brain samples taken from patients who suffered from Alzheimer’s disease and from senile involution cortical changes^95^. ICP-MS was used also for multielemental (48 elements) analysis of human lung samples collected during surgical procedures. Dependencies between elemental composition and the patient gender, nicotine smoking and occupational exposure to metals were examined^18^. Boulyga et al. performed elemental analysis of thyroid tissue taken from people living in the Chernobyl area. Despite the small quantities of examined material (down to 1 mg) they obtained high sensitivity of measurements. In the case of iodine determination, they observed losses of this element due to its volatility and high ionization potential. Based on the results obtained from the measurements of certified reference materials (CRM) they calculated correction factor for quantitative analysis of iodine in sample^38^. The ICP-MS was also applied for multielemental analysis of esophageal tissues taken from patients suffered from esophageal squamous cell carcinoma. Concentrations of elements in normal tissue, cancerous tissue and its surrounding area were compared^96^. Using ICP-MS the content of Ca, Cu, Fe, As, Mg, Ni, Cd and Cr in stomach tissue taken from patients with cancer was also determined^97^. In turn, Jablonska et al. measured the concentrations of Cd, As, Se and Fe in breast cancer tissue^65^. Varga et al. examined concentrations of Cr, Mn, Fe, Ni, Cu, Zn, Rb and Pb in liver samples taken from patients suspected with chronic diffuse liver disease^39^ whilst Sahin et al. determined Al, Fe, Cd, Mn, Cr, Cu, Pb, Ni, Zn, Ag and Co concentrations in liver of patients suffered from hepatitis B^98^. The technique was also used for determination of Cr, Co and V concentrations in heart samples^99^.

Batista et al. used ICP-MS for multielemental analysis of human hair, previously prepared for analysis using ultrasound extraction. However, the method was not useful for Ag, Se and Mo determinations^100^. Prejac et al. analysed human hair and blood for strontium content, assuming that hair can be a long-term biological indicator of nutrition in case of this element^23^. Using ICP-MS, MacLachlan et al. determined the content of As, Cd, Co, Pb, Hg, Mo and Se in liver, kidney and muscle from Australian sheep^76^ whilst Garcia-Vaquero et al. examined changes of As, Ca, Cd, Co, Cr, Fe, Hg, Mn, Mo, Ni, Pb, Se, Sn and Zn concentrations in organs of beef calves which were induced by Cu dietary supplementation^101^. In turn, Gui et al. used ICP-MS to measure the concentration of Zr in liver, kidney and urine of rats^102^.

**Figure 4:**
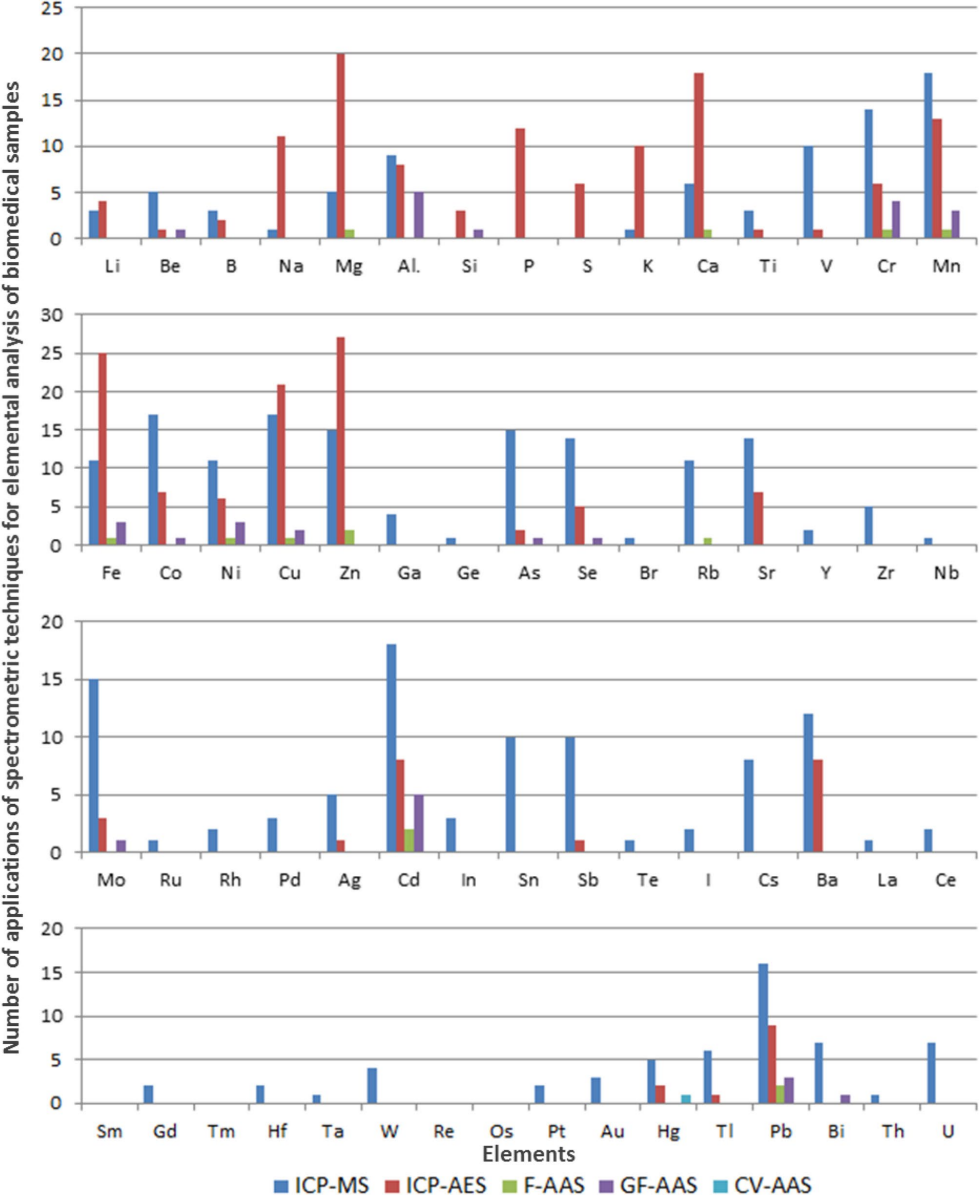
Number of applications of particular analytical technique for elemental analysis of biomedical samples depending on the determined element (based on the papers cited in the following article).

Based on the available literature data, charts were prepared to show the frequency of application of selected analytical techniques for the determination of particular elements in biomedical samples. They are placed in the Fig. 4.

As it can be seen from the Fig. 4, the most commonly used techniques of elemental analysis of biomedical samples are ICP-MS and ICP-OES. Probably this results from the fact that they make possible simultaneous multielemental analysis, in contrast to AAS technique usually allowing the determination of single elements. ICP-OES is usually selected for the analysis of major and minor elements, while ICP-MS for trace and ultra-trace ones. This is because ICP-MS offers the lowest detection limits amongst other discussed techniques of elemental analysis. ICP-MS is more versatile, however it is also more complicated and much more expensive comparing to ICP-OES. Therefore, ICP-OES can be a good alternative to ICP-MS, especially in case of analysis of elements occurring in samples at higher concentrations. Such approach was utilized by Harrington et al. who performed a multielemental analysis of human blood and serum. For this purpose, they used the ICP-OES to determine Ca, Mg, Na, K, and ICP-MS for Co, Zn, Cu, Se, Mo, Cr, Mn and Fe^32^. Forte et al. determined Ca, Cu, Fe, Mg, Si and Zn in human body fluids using ICP-OES, whilst concentrations of Al and Mn with the use of ICP-MS^81^. Similar approaches were applied in the works of Korvela et al.^16^, Shimamura et al.^28, 103^, Takahashi et al.^13^, MacLachlan et al.^76^ and Alimonti et al.^37^*.*

## Preparation of biological samples for elemental analysis

In order to enable the analysis of biomedical samples using discussed instrumental techniques removing of its organic matrix is a crucial step. This process is called mineralization and results in oxidation of the hydrogen contained in sample to water, carbon to carbon dioxide and appearing of the free nitrogen. Usually, the sample is decomposed with the use of oxidizing acids in conditions of higher temperature. As a result of mineralization, the volatile, organic components are removed from the sample whilst its inorganic part is transformed to solution which can be analysed.

Based on the information contained in the papers dealing with the elemental analysis of biomedical samples, a summary concerning the used sample preparation methods was made and is placed in Table 1. It can be seen that the most popular is microwave-assisted acid digestion, which is used for liquids and soft or hard tissues dissolution. Microwave energy is supplied directly to the sample, which causes its effective heating and improves its decomposition. The conditions of microwave heating process, such as temperature and duration is very different. Slightly less often the mineralization of samples in acids with the use of thermal conductivity is used. For this purpose, samples with acid addition are heated either in laboratory ovens or on hotplates. Since the thermal energy is not transferred directly to the sample, this method is less effective than the one using microwave energy. The digestion using both microwave energy and thermal conductivity, can be carried out in open and closed systems (the samples are placed in Teflon vessels and sealed). The advantage of the method based on a closed system is that in such conditions, high pressure is generated in the vessel due to the evolution of gases (volatile components of the sample). With increasing pressure in the vessel, the boiling point of acids used for digestion also increases, which in turn makes possible to use higher temperatures for sample decomposition than in open systems. This allows to decompose the sample more efficiently and faster than in traditional open systems. Also, the probability of sample contamination with components from the environment is much lower. Since direct information about whether digestions were carried out in an open or closed system is rarely placed in papers, such a distinction has not been prepared. The mineralization of biomedical samples by using only acid digestion, without microwave or thermal support, is the least common method. However, one can find examples of its use for mineralization of blood, organ tissues and bones.

**Table 1:** Summary of methods used for preparation of biomedical samples for elemental analysis (based on the literature data).

Sample preparation (method of digestion)		Type of sample
Microwave-assisted acid digestion	Wet sample	Brain^19, 20, 101^, stomach^19, 46, 97^, liver^19, 20, 46, 77, 101, 102^, kidney^19, 20, 46, 101, 102^, pancreas^20^, spleen^20, 101^, heart^19, 20^, lung^19^, diaphragm^101^, breast^65^, hair^42, 46, 64, 100^, blood^23, 32, 46, 59, 60, 81, 92^, serum^32, 82, 83^, amniotic fluid^17^, urine^59, 60, 102, 104^
	Dry sample	Brain^9, 45, 51, 71, 72, 95, 105^, liver^13, 39, 41, 58, 79^, kidney^79, 80^, heart^79, 99^, thyroid^38^, lung^18, 79^, esophageal^96^, muscle^41^, breast^57^, arteries^69^, hair^23, 59, 60^, gallstones^25^, bone^12^
Thermal-heating acid digestion	Wet sample	Brain^78, 94, 106^, cerebrum^21^, liver^8, 21, 78^, kidney^21, 28, 78^, spleen^21^, colon^36^, heart^78^, lung^21^, muscle^78^, breast^22^, skin^21^, hair^86^, blood^59, 63, 107^, serum^63, 90^, bone-marrow fluid^35^, urine^44, 59^, teeth^26^, bone^78, 84^
	Dry sample	Brain^27, 45, 71–73^, liver^98, 108^, muscle^108^, breast^55, 56, 109^, arteries^68^, uteri round ligaments^70^, hair^24, 34, 44, 59^, fingernail^24^, bone^6^
Acid digestion	Wet sample	Stomach^46^, liver^46, 76^, kidney^46, 76^, muscle^76^, hair^46, 47^, blood^46^
	Dry sample	Liver^110^, bone^85^
**Sample preparation without digestion procedure**		
Preparation of powdered sample followed by dilution in water to obtain slurry		Hair^53^
Dilution with Triton X-100 and chemical modifier		Serum^12^
Dilution with matrix modifier		Serum^55^
Dilution with PTFE emulsion, Triton X-100 and water		Serum^47^
Dilution with Triton X-100		Serum^79^
Dilution with acid and internal standard		Plasma^89^
Dilution with Triton X-100, diammonium hydrogen phosphate (as matrix modifier) and acid		Blood^14^
Dilution with internal standard and Triton X-100		Blood^107^
Dilution with Triton X-100, internal standard and ammonia solution		Blood^8, 88^, plasma^8^, erythrocytes^8^
Dilution with acid		Cerebrospinal fluid^16^
Dilution with hydrogen peroxide, acid and Triton X-100		Urine^15^
Dilution with acid and water		Urine^81, 91^
Dilution with acid, internal standard and ethanol		Urine^8^
Dilution with water		Serum^81^, cerebrospinal fluid^37, 81^, urine^8^
Dilution with internal standard		Serum^111^, cerebrospinal fluid^111^

As one can notice from Table 1, a very popular procedure of body fluids preparation is the dilution of samples (with distilled water or acid) or adding to them various compounds. However, it should be remembered that the dilution of samples can be a problem when the elements under analysis occur in the sample at very low concentrations. Matrix modifiers are added to samples before GF-AAS analysis to reduce the measurement interferences^53,55,57,59,108^. PTFE (polytetrofluoroethylene) slurry is used as a chemical modifier for direct serum analysis by ICP-OES with electrothermal vaporization as a method of sample introduction^47^. Triton X-100 is a detergent applied to dissolve proteins and lipids of cell membranes, often used for blood^8,14,88,107^, serum^12,47,79^ and plasma^8^ preparation. For these types of samples, ammonium compounds are often added as they prevent the coagulation of sample components^8,14,88^.

## Parameters used for validation of analytical techniques

### Precision

Precision is a parameter that characterizes the closeness between the results of independently carried out measurements. It is the result of random errors occurring during the measurement procedure by using the given analytical technique^112^. It is expressed as a standard deviation (SD), relative standard deviation (RSD, which is equal to SD divided by the mean measurement result) and most often as a coefficient of variance (CV or also VC, CV[%] = RSD*100). Repeatability, intermediate precision and reproducibility are distinguished in term of precision^113^. It is said repeatability when there is no long-time interval between the considered measurements and they are performed on the same equipment in a laboratory by one operator^114^. In the literature, repeatability is also referred to as intra-day precision (for measurements performed on the same day), intra-assay precision (or within-run—repeatability of results obtained in a given measurement series) or inter-assay precision. Intermediate precision refers to the deviation between the results obtained in the same laboratory when the measurements were carried out over a longer period of time (e.g. several weeks). In addition, it is not necessary for the measurements to be carried out by the same operator using the same instrument. Different laboratory reagents and accessories may also be used^114^. The terms commonly used in the literature in respect to inter-mediate precision are inter-day precision, within-laboratory reproducibility, day-to-day precision. The least used term in the literature in relation to precision is reproducibility. It expresses a standard deviation of the results obtained by a given technique in different laboratories. It is recommended that the determination of the measurement precision should be carried out using the test sample, with matrix and the concentration of the elements as similar as possible to the authentically analysed sample^115^. In the literature, however, one can find out the values of measurement precision for reference and standard materials as well. There are many recommendations for determining precision and there is no single fixed formula. It is important, however, that the experimentalists put in their work information how this validation parameter was obtained. Discrepancies that can be observed concern the type of sample for which measurements are made, the number of measurements carried out in a given series and the number of series as well as concentration levels for which precision is determined.

### Trueness

Trueness is a parameter that defines the closeness of the obtained result, understood as the average value from the measurement series, to the expected value. It expresses the systematic error of measurement appearing with the use of a given analytical technique^114^. In order to determine the trueness, certified reference materials are most frequently used. The value of trueness for a given technique can be obtained also using another analytical technique, called the reference. Often, instead of determining the trueness using CRMs, or as an additional option, the “spike and recovery” method is used. It involves adding a known quantity of the analyte to the previously measured sample and afterwards its re-measuring. In such a case, the trueness is referred to as recovery. If the enrichment of the sample with the analyte took place at an early stage of its pre-treatment, calculated trueness also takes into account the procedure of sample preparation^113, 115^. Often in scientific articles, only experimental and certified values are given, without indicating the value of trueness. The most often used formula for trueness calculation is Trueness[%] = (experimental value/certified value)*100. Then, the result closer to 100% indicates better trueness. The same dependence occurs for recovery (Recovery[%] = ((c1−c2)/c3)*100, where c1 is the concentration of the analyte after sample spiking, c2 is the concentration of the analyte before sample spiking and c3 is a concentration of spiked analyte). One can also meet the trueness expressed as Bias or Mean Relative Error and calculated according to the formula Bias[%] = [experimental value of concentration-certified value of concentration (or known, added concentration)]/certified value (or added)*100. In this case, a smaller measurement error will result in a closer to 0% Bias value. In literature, the word "accuracy" is often used instead of trueness. This is improper, due to the fact that accuracy concerns the correspondence between the true value and the result of a single measurement (not an average value). The accuracy of the measurement (the magnitude of the total error) consists of trueness (systematic error) as well as precision (random error)^112,116^. Both accuracy and trueness, as well as trueness and recovery seem to be often used interchangeably in scientific articles, which is also not correct. Therefore, the details concerning the method of their determination is very important for proper interpretation of the results.

### Limit of detection

Limit of detection (LOD) is a parameter that indicates the smallest content of an analyte that can be detected with a certain probability and by using a given analytical procedure but not necessarily quantified^117^. It can be expressed as micrograms per gram or micrograms per litre units. In the literature, various possibilities to calculate the limit of detection can be found. The most common formula used for LOD calculations is LOD = 3*SDblank, where SDblank is the standard deviation of the analyte content in blank. It is recommended to calculate the standard deviation for 10 independent measurements^115^ however in scientific articles their number differs. One can also find the following formula for LOD calculation LOD=$\stackrel{-}{x}$blank + 3*SDblank. It is used when the level of the determined analyte is measurable in blank solution^110^. The detection limit can also be expressed in the signal domain according to the formula: yd=$\stackrel{-}{x}$blank + 2*t*SDblank, where yd is the average value of a signal for a blank and t is Student’s t-distribution coefficient. Then, from the calibration curve and based on the detection limit in the signal domain, the value corresponding to the concentration unit of the measured analyte is read^82^. Discussing the detection limit, one can also distinguish the value associated with used instrument (Instrument Detection Limit, IDL) and the applied measurement method (Method Detection Limit, MDL). The first one is determined on the basis of measurements made for standard solutions (blank measurement), which have not been subjected to any preparation. In turn, MDL determines the limit of detection for the method, thus it is influenced by all stages of sample preparation for analysis. MDL values are always greater than those of IDL (determined for the same analytical technique)^17,114^.

## The comparison of the discussed techniques of elemental analysis in respect of their use for the analysis of biological samples and achievable validation parameters

Tables 2, 3, and 4 summarize the values of selected validation parameters determined in research papers dealing with the elemental analysis of biomedical samples using various instrumental techniques. Additionally in the Table S1 and S2 of Supplementary materials, the details concerning the type of samples and the methods of their preparation as well as used instrument and experimental conditions in particular papers were placed. The validation parameters taken into account during data collection were the precision of measurements, the trueness and the limit of detection for the analysed element. When it was possible, an attempt was made to distinguish the parameter characterizing precision into repeatability, inter-day (assay) precision and reproducibility. For the trueness, based on the information contained in the papers, it was indicated whether it was calculated based on the CRM measurements or using the sample spiking method. If the publications contained only information on the certified and measured value of the concentration of the analysed element, to enable the comparisons, the trueness was calculated according to the following formula Trueness[%] = (experimental value/certified value)*100. It was also marked if trueness was calculated as Bias. A large variety of methods for calculating the detection limits of elements was noticed. Therefore, only the type of matrix for which the detection limit of the analysed element was indicated, whether it was a blank solution or the analysed real biomedical sample. Also, values of LODs are expressed in both volume and mass units. Since there is a lack of information necessary to standardize them, the original format of units has been left.

**Table 2:** Values of precision, trueness and detection limit for different elements determined by using AAS.

Element	Precision (%CV) a: intra-day precision b: inter-day precision c: reproducibility	Trueness (%) a: calculated with CRM b: as recovery	Limit of detection a: in blank solution b: in real sample
**CV AAS**			
Hg	**a: ** below 3.7^46^	**a: ** 100.0–104.5^46^ **b: **89.9–99.2^46^	**a: ** 0.0003 μg/mL^46^ 0.00019 μg/mL^46^
**F AAS**			
Mg		**b: ** 98.1^34^	**a: ** 0.05 μg/mL^34^
Ca		**b: ** 102.4^34^	**a: ** 0.01 μg/g^34^
Cr		**b: ** 102.4^34^	**a: ** 0.05 μg/g^34^
Mn	a: 3.2^64^	**a: ** 97.1–99.2^64^ **b: ** 99.4^64^ 99.3^64^	**a: ** 0.000097 μg/mL^64^
Fe		**b: ** 97.8^34^	**a: ** 0.08 μg/g^34^
Ni		**a: ** 98.8^63^ 99.2^63^	**a: ** 0.00052 μg/mL^63^
Cu		**b: ** 104.1^34^	**a: ** 0.03 μg/g^34^
Zn		**a: ** 95.9^110^* 95.1^110^* **b: **95.2^34^	**a: ** 0.03 μg/g^34^
Cd	**nd: ** 2.1^24^	**b: ** 96.0–99.0^24^ 94.6^34^	**a: ** 0.00011 μg/mL^24^ 0.03 μg/g^34^
Pb	**nd: **1.9^24^	**b: ** 95.8–101.2^24^ 96.0^34^	**a: ** 0.0003 μg/mL^24^ 0.03 μg/g^34^
**GF AAS**			
Be			**b: ** 0.000007 μg/mL^5^ 0.000002 μg/mL^5^
Al	**a: ** 1.9–9.0^53^ 0.8–2.8^12^ **b: **5.8–7.2^12^ **nd: ** ± 7.15^15^	**a: ** 83.3^15^ 100^15^ 42.8^51^ 97.6^51^ 52^57^ **b: ** 67–98^51^ 97.0 ± 3.8–103.4 ± 2.8^53^ 98–109^12^ 103^57^	**a: ** 0.9 μg/mL^53^ 0.00023 μg/mL^12^ 0.00035 μg/mL^12^ 0.00048 μg/mL^57^ 0.0011 μg/mL^15^ **b: ** 0.000023 μg/mL^12^ 0.001 μg/mL^12^
Si	**nd: ** 8.4^55^ 5.9^55^	**b: ** 97–104^55^	
Cr	**a: ** 2.0–2.9^108^ **c: ** 5.35–6.59^59^ 1.9–4.1^108^ **nd: ** ± 4.37^15^	**a: ** 101.2^15^ 107.7^15^ 99.0–99.9^59^* 91.76–102.49^108^ **b: ** 83.1–86.3^108^	**a: ** 0.0000062 μg/mL^59^ 0.00042 μg/mL^15^ **nd: ** 0.001 μg/g^108^
Mn	**a: ** 0.5–3.3^53^ **nd: ** 7.26–9.17^60^	**a: ** 99–99.3^60^* 98^45^* 99.1^110^* 95.3^110^* **b: ** 99.5 ± 0.8–103.3 ± 1.2^53^	**a: ** 27.6 μg/g^53^ 0.00013 μg/g^60^ **nd: ** 0.6 μg/g^45^
Fe		**a: ** 97^45^* 106^105^* 101^105^*	**nd: ** 1.6 μg/g^45^
Co	**nd: ** 7.69^60^ 0.80^60^ 6.30^60^	**a: ** 98.9–99.6^60^*	**a: ** 0.0013 μg/g^60^
Ni	**c: ** 3.06–8.24^59^ **nd: ** 1.52^19^ (standard solution) 10.4^19^ (sample) 4.38^19^ (sample)	**a: ** + 27.4^19^^#^ − 2.9^19^^#^ + 52.5^19^^#^ + 41.7^19^^#^ + 8.1^19^^#^ − 24.3^19^^#^ + 59.3^19^^#^ 99.8–100.3^59^* **b: ** 92.1–123.5^19^	**a: ** 0.00023 μg/mL^19^ 0.000025 μg/g^59^
Cu	**nd: ** 7.35^60^ 1.29^60^ 6.1^60^	**a: ** 96.4–99.8^60^* 99^45^* 102.5^110^* 103.8^110^*	**a: ** 0.00000017 μg/g^60^ **nd: ** 0.2 μg/g^45^
Zn		**a: ** 97^45^*	**nd: ** 0.3 μg/g^45^
As	**nd: ** 4.73^60^ 6.2^60^	**a: ** 99.4^60^* 103.2^60^*	**a: ** 0.0000159 μg/g^60^
Mo	**nd: ** ± 5.9^15^	**b: ** 95.3–103.0^15^	**a: ** 0.00081 μg/mL^15^
Cd	**c: ** 4.72–7.69^59^ **nd: ** 3.6^14^ 3.2^14^ (standard material)	**a: ** 100.3–102^59^* 86^45^* 105^14^ 115^14^	**a: ** 0.00018 μg/g^56^ 0.00002 μg/g^59^ 0.005 μg/mL^14^ **nd: ** 0.02 μg/g^45^
Pb	**c: ** 3.63–7.76^59^	**a: ** 100.5–100.9^59^* 106^45^*	**a: ** 0.00157 μg/g^56^ 0.001 μg/g^59^ **nd: ** 0.1 μg/g^45^
Bi	**nd: ** 4.3^44^ 4.7^44^	**a: ** 96.8^44^ **b: ** 97.7^44^ 101.1^44^	**a: ** 0.00002 μg/mL^44^ 0.0015 μg/mL^44^

*nd* method of parameter determination was not defined;

^#^Trueness expressed as a Bias;

*Calculated based on the literature data.

**Table 3:** Values of precision, trueness and detection limit for different elements determined by using ICP-OES.

Element	Precision (%CV) a: intra-day precision b: inter-day precision c: reproducibility	Trueness (%) a: calculated with CRM b: as recovery	Limit of detection a: in blank solution b: in real sample
**Li**	**a: ** 12.9^83^ **b: ** 9.0^83^	**b: ** 90.2^82^ 110^83^	**a: ** 0.0033 μg/mL^82^ 0.000278 μg/mL^83^* **nd: ** 0.042 μg/mL^77^
**Be**		**b: ** 103^83^	**a: ** 0.000216 μg/mL^83^*
**B**	**a: ** 2.0^83^ **b: ** 4.5^83^	**b: ** 96^83^	**a: ** 0.002162 μg/mL^83^*
**Na**	**nd: ** 0.8–3.6^13^ 3^16^ 2^35^	**a: ** 91^71^* 96–103^13^ **b: ** 89.1^82^	**a: ** 0.402 μg/mL^13^ **b: ** 0.4 μg/mL^16^ 0.013 μg/mL^82^ **nd: ** 0.11 μg/mL^78^
**Mg**	**nd: ** 1.7–2.0^13^ 7^16^ 3^35^	**a: ** 99^69^ 100^71^* 97^79^ 95^13^ 98.5^84^* **b: ** 92–104^69^ 83^79^	**a: ** 0.02 μg/mL^79^ 0.931 μg/mL^13^ **b: ** 0.04 μg/g^69^ 0.1 μg/mL^16^ **nd:** 0.000015 μg/mL^78^
**Al**	**nd: ** 60^35^	**a:** 101^3^* 96^3^* **b: ** 88.9^82^ 107^83^	**a: ** 0.0005 μg/mL^82^ 0.0243 μg/mL^83^*
**Si**	**nd: ** 5.3^81^		
**P**	**nd: ** 1.0^13^ 2.0^16^ 3.0^35^	**a: ** 90–98^13^ 99.8^84^*	**a: ** 0.23 μg/mL^13^ **b: ** 2.0 μg/mL^16^ **nd:** 0.033 μg/mL^78^
**S**			**nd:** 0.035 μg/mL^78^
**K**	**nd: ** 2.0–4.7^13^ 2^16^ 0.6^35^	**a: ** 98^71^* 72–73^13^ 104^84^* **b:** 89.3^82^	**a: ** 0.065 μg/mL^82^ 1.146 μg/mL^13^ **b:** 5.9 μg/mL^16^ **nd:** 0.41 μg/mL^78^
**Ca**	**nd:** 5^13^ 6^16^ 2.0^35^	**a:** 96^47^ 102^69^ 94^71^* 101–104^13^ 100.6^84^* **b:** 96–105^69^ 101^81^	**a:** 0.023 μg/mL^13^ **b:** 0.005 μg/g^69^ 0.1 μg/mL^16^ **nd:** 0.00001 μg/mL^78^
**Ti**		**a:** 89^47^	
**Cr**	**a:** 7.8^83^ **b:** 39.6^83^	**a:** 111^47^ 98^3^* 84^3^* **b:** 100^83^	**a:** 0.00364 μg/mL^83^* **nd:** 0.000366 μg/mL^77^
**Mn**	**a:** 14.7^83^ **b:** 26.6^83^ **nd:** 10–15^25^	**a:** 88–103^3^* 98^45^* 112^71^* 85.7^83^* 96^86^* 88^86^* **b:** 88.6^82^ 96^83^	**a:** 0.00054 μg/mL^82^ 0.00011 μg/mL^83^* **nd:** 0.4 μg/g^45^ 0.000403 μg/mL^77^ 0.00025 μg/mL^78^
**Fe**	**a:** 0.5^83^ **b:** 1.9^83^ **nd:** 1.9–2.2^13^ 2^35^ 25^25^	**a:** 113^47^ 100.3–104.9^3^* 99.7^69^ 90^79^ 94–98^13^ 91.2^83^* 93^84^* 106^86^* 92^86^* **b:** 91^79^ 93–105^69^ 91.5 ± 1.8^82^ 95^83^	**a:** 0.038 μg/mL^79^ 0.459 μg/mL^13^ 0.00091 μg/mL^82^ 0.001117 μg/mL^83^* **b:** 0.5 μg/g^69^ **nd:** 2.3 μg/g^45^ 0.000562 μg/mL^77^ 0.0009 μg/mL^78^
**Co**		**a:** 105^3^* 99^3^* 100^83^* 95^86^* 97^86^* **b:** 91^83^	**a:** 0.00825 μg/mL^83^* **nd:** 0.00024 μg/mL^77^
**Ni**		**a:** 103^3^* 92^3^* 96^86^* 109^86^* **b:** 93^83^	**a:** 0.002935 μg/mL^83^* **nd:** 0.00114 μg/mL^77^
**Cu**	**a:** 0.6^83^ **b:** 2.3^83^ **c:** 11.1^26^ **nd:** 2.9^81^ below 5^25^	**a:** 90^47^ 101–107^3^* 99^45^* 106^71^* 102^76^* 94^79^ 91–107^81^ 103.4^83^* 99^86^* 108^86^* **b:** 95^79^ 95^81^ 97^83^	**a:** 0.0109 μg/g^76^ 0.003 μg/mL^79^ 0.003178 μg/mL^83^* **nd:** 2.1 μg/g^45^ 0.000588 μg/mL^77^ 0.0004 μg/mL^78^
**Zn**	**a:** 0.3^83^ **b:** 1.3^83^ **c:** 1.55^26^ **nd:** 10–15^25^	**a:** 108^47^ 93–101^3^* 103^45^* 113^71^* 103^76^* 87^79^ 92–105^81^ 101^83^* 97^84^* 93^86^* 106^86^* **b:** 87^79^ 90.7^82^ 110^83^	**a:** 0.0357 μg/g^76^ 0.012 μg/mL^79^ 0.0039 μg/mL^82^ 0.000981 μg/mL^83^* **nd:** 1.1 μg/g^45^ 0.000391 μg/mL^77^ 0.0014 μg/mL^78^
**Se**	**a:** 9.3^83^ **b:** 9.1^83^	**a:** 95–109^3^* 94.5^83^* **b:** 91^83^	**a:** 0.036 μg/mL^79^ 0.022898 μg/mL^83^* **nd:** 0.0002 μg/g^56^
**Sr**	**a:** 2.3^83^ **b:** 4.6^83^	**a:** 88–101^3^* 95^84^* **b:** 104^83^	**a:** 0.000175 μg/mL^83^* **nd:** 0.00138 μg/mL^77^ 0.00002 μg/mL^78^
**Mo**			**nd:** 0.000784 μg/mL^77^ 0.0006 μg/mL^78^
**Cd**	**a:** 23.7^83^ **b:** 18.0^83^	**a:** 92–102^3^* 91^45^* 112^79^ 115^86^* 85^86^* **b:** 90^83^ 116^79^	**a:** 0.001 μg/mL^79^ 0.000337 μg/mL^83^* **nd:** 0.1 μg/g^45^ 0.000132 μg/mL^77^
**Ba**	**a:** 0.6^83^ **b:** 1.5^83^ **nd:** below 6^118^	**a:** 4^118^^#^ 7^118^^#^ **b:** 105^83^ 76–104^118^ 85–101^118^	**a:** 0.000412 μg/mL^83^* 0.00011 μg/mL^118^ **nd:** 0.000531 μg/mL^77^ 0.00006 μg/mL^78^
**Hg**	**a:** below 6^46^	**a:** 80^46^	**a:** 0.00002 μg/mL^46^ 0.000007 μg/mL^46^ 0.023 μg/g^46^ **nd:** 0.00553 μg/mL^77^
**Pb**	**c:** 10.3^26^	**a:** 102–113^3^* 83^86^* **b:** 103^83^	**a:** 0.019 μg/mL^79^ 0.007874 μg/mL^83^* **nd:** 2.0 μg/g^45^ 0.00343 μg/mL^77^

*nd* method of parameter determination was not defined;

^#^Trueness expressed as a Bias;

*Calculated based on the literature data.

**Table 4:** Values of precision, trueness and detection limit for different elements determined by using ICP-MS.

Element	Precision (% CV) a: repeatability b: inter-day (assay) c: reproducibility	Trueness (%) a: calculated with CRM b: as recovery	Limit of detection a: in blank solution b: in real sample
**Li**	**nd: ** 5.6^37^	**a:** 105^37^* **b:** 102^37^*	**a:** 0.004 μg/g^18^ 0.000007 μg/mL^37^
**Be**			**a:** 0.0001 μg/g^18^ 0.00009 μg/g^100^
**B**	**a:** 3.7^89^ **b:** 10.8^89^	**b:** 98.04^89^	**a:** 0.47 μg/g^18^
**Na**	**nd:** 1.0^119^ 1.2^119^	**a:** 103.1^38^* 94^39^* 108^119^	**a:** 1 μg/g^38^ **nd:** 0.0002 μg/mL^119^
**Mg**	**a:** 4.0^17^ 0.79^96^ 0.6^90^ **b:** 1.6^90^ 5.0^17^ **nd:** 1.5^119^	**a:** 93^17^ 99^9^* 93. 2^38^* 95^96^* 99^39^* 103.3^90^	**a:** 0.0004 μg/mL^17^ (IDL) 0.00088 μg/mL^17^ (MDL) 5 μg/g^38^ **b:** 0.00234 μg/mL^90^ **nd:** 0.0004218 μg/mL^96^ 0.00007 μg/mL^119^
**Al**	**a:** 1.6^17^ 1.28^96^ 7.4^89^ **b:** 2.3^17^ 12.7^89^ **nd:** 6.2–6.7^13^	**a:** 66^13^ 113^17^ 115^96^* **b:** 89.82^89^	**a:** 0.000029 μg/mL^13^ 0.00081 μg/mL^17^ (IDL) 0.0026 μg/mL^17^ (MDL) 0.388 μg/g^18^ 0.0001 μg/g^100^ **nd:** 0.0023464 μg/mL^96^ 0.00174 μg/g^120^
**P**	**nd:** 0.9^119^ 3.9^119^	**a:** 104^119^	**nd:** 0.000012 μg/mL^119^
**S**	**nd:** 0.8^119^ 3.6^119^	**a:** 100^119^	**nd:** 0.0003 μg/mL^119^
**K**	**nd:** 1.2^119^ 3.1^119^	**a:** 98.7^38^* 98^119^	**a:** 3 μg/g^38^ **nd:** 0.011 μg/mL^119^
**Ca**	**a:** 1.2^90^ 5.2^17^ 3.61^96^ **b:** 6.4^17^ 2.5^90^ **nd:** 2.1^119^ 9.5^119^	**a:** 95^17^ 100^9^* 96.9^38^* 64^96^* 105^39^* 103.9^90^ 93^119^	**a:** 0.015 μg/mL^17^ (IDL) 0.056 μg/mL^17^ (MDL) 0.801 μg/g^101^ **b:** 0.04088 μg/mL^90^ **nd:** 0.0025794 μg/mL^96^ 0.00003 μg/mL^119^
**Ti**	**a:** 3.90^96^ **nd:** 4^16^		**a:** 0.048 μg/g^18^ **b:** 0.5 μg/mL^16^ **nd:** 0.0001132 μg/mL^96^
**V**	**a:** 5.2^17^ 1.91^96^ 4.7^89^ **b:** 10.9^89^ 8.4^17^	**a:** 107^17^ 104.7^38^* 67^96^* 111.22^89^	**a:** 0.0000017 μg/mL^17^ (IDL) 0.0000027 μg/mL^17^ (MDL) 0.014 μg/g^18^ 0.005 μg/g^38^ 0.0001 μg/g^100^ **nd:** 0.0000038 μg/mL^96^ 0.000135 μg/mL^99^
**Cr**	**a:** 3.4^17^ 3.31^96^ 3.8^89^ **b:** 4.4^17^ 5.6^89^ **nd:** 3.1^119^	**a:** 88^17^ 125.9^38^* 100^96^* 106.25^89^ 63^101^*	**a:** 0.000042 μg/mL^17^ (IDL) 0.000052 μg/mL^17^ (MDL) 0.024 μg/g^18^ 0.3 μg/g^38^ 0.0001 μg/g^100^ 0.0014 μg/g^101^ **b:** 0.00005 μg/mL^8^ 0.0001 μg/mL^8^ 0.0001 μg/mL^8^ 0.00025 μg/mL^8^ **nd:** 0.0000649 μg/mL^96^ 0.00113 μg/g^98^ 0.000116 μg/mL^99^ 0.0000014 μg/mL^119^
**Mn**	**a:** 4.5^17^ 2.05^96^ 3.3^89^ **b:** 10.0^17^ 4.5^89^ **nd:** 2.1–3.6^13^ 1.1^100^ 1.1^100^ 2.9^119^ 2.7^119^	**a:** 99–110^13^ 94^17^ 97^9^* 97.6^38^* 87^96^* 97^39^* 99^100^* 93^100^* 104^101^* 104^119^ **b:** 102.52^89^	**a:** 0.000071 μg/mL^13^ 0.000012 μg/mL^17^ (IDL) 0.000025 μg/mL^17^ (MDL) 0.018 μg/g^18^ 0.07 μg/g^38^ 0.0016 μg/g^101^ 0.001 μg/g^100^ **nd:** 0.0000194 μg/mL^96^ 0.00021 μg/mL^98^ 0.000003 μg/mL^119^
**Fe**	**a:** 1.13^96^ 1.5^89^ 2.4^90^ **b:** 3.7^89^ 4.1^90^ **nd:** 2.5^119^ 4.1^119^	**a:** 98^9^* 102.0^38^* 97^96^* 95^39^* 97.9^90^ 111^101^* 71^101^* 104^119^ **b:** 102.66^89^	**a:** 0.089 μg/g^18^ 4 μg/g^38^ 0.136 μg/g^101^ **b:** 0.00205 μg/mL^90^ **nd:** 0.0001827 μg/mL^96^ 0.00149 μg/g^98^ 0.00002 μg/mL^119^
**Co**	**a:** 3.5^17^ 4.10^96^ 3.7^89^ **b:** 5.6^17^ 9.6^89^ **nd:** 1.1–8.2^13^ 4.6^119^	**a:** 92^76^* 92–100^13^ 96^17^ 77.2^38^* 110^39^* 105.09^89^ **b:** 89^101^*	**a:** 0.004 μg/g^76^ 0.000016 μg/mL^13^ 0.0000043 μg/mL^17^ (IDL) 0.0000061 μg/mL^17^ (MDL) 0.001 μg/g^18^ 0.007 μg/g^38^ 0.00004 μg/g^100^ 0.0002 μg/g^101^ **nd:** 0.0000061 μg/mL^96^ 0.00024 μg/g^98^ 0.000177 μg/mL^99^ 0.0000008 μg/mL^119^
**Ni**	**a:** 2.8^17^ 5.40^96^ **b:** 6.2^17^ **nd:** 5.6^119^	**a:** 110^17^ 88.9^38^* 100^96^* 138^101^*	**a:** 0.0004 μg/mL^17^ (IDL) 0.00051 μg/mL^17^ (MDL) 0.016 μg/g^18^ 0.1 μg/g^38^ 0.0025 μg/g^101^ **nd:** 0.0000405 μg/mL^96^ 0.00064 μg/g^98^ 0.00002 μg/mL^119^
**Cu**	**a:** 2.1^17^ 1.26^96^ 1.3^89^ 1.5^90^ **b:** 3.9^17^ 9.7^89^ 4.2^90^ **nd:** 1.3–4.1^13^ 2^35^ 1.9^100^ 0.7^100^ 2.9^119^ 2.9^119^	**a:** 89–97^13^ 92^9^* 96.5^38^* 125^101^* 100^101^* 94^39^* 100^96^* 107.5^90^ 95.87^89^ 89^17^ 95^100^* 81^100^* 107^119^	**a:** 0.000017 μg/mL^13^ 0.000039 μg/mL^17^ (IDL) 0.000072 μg/mL^17^ (MDL) 0.049 μg/g^18^ 0.1 μg/g^38^ 0.0029 μg/g^100^ 0.0304 μg/g^101^ **b:** 0.00010 μg/mL^90^ **nd:** 0.0000399 μg/mL^96^ 0.00059 μg/g^98^ 0.000007 μg/mL^119^
**Zn**	**a:** 5.3^17^ 0.90^96^ 1.1^89^ 3.6^90^ **b:** 7.5^17^ 8.7^89^ 5.3^90^ **nd:** 1.5–3.4^13^ 22.8^95^ 16.3^95^ 1.2^100^ 0.2^100^ 2.4^119^ 5.3^119^	**a:** 93–97^13^ 88^17^ 116^9^* 101–104^95^* 101.2^38^* 102^96^* 102^39^* 94.39^89^ 97.3^90^ 110^101^* 91^101^* 97^100^* 98^100^* 108^119^ **b:** 111 ± 30^95^ 99 ± 12^95^	**a:** 0.000017 μg/mL^13^ 0.00096 μg/mL^17^ (IDL) 0.002 μg/mL^17^ (MDL) 0.0107 μg/g (32 μg/mL)^95^ 0.58 μg/g^18^ 0.2 μg/g^38^ 0.0042 μg/g^100^ 0.9 μg/g^101^ **b:** 0.00024 μg/mL^90^ **nd:** 0.0005154 μg/mL^96^ 0.00251 μg/g^98^ 0.00002 μg/mL^119^
**Ga**	**a:** 2.47^96^		**a:** 0.0007 μg/g^18^ **nd:** 0.0000452 μg/mL^96^
**Ge**			**a:** 0.001 μg/g^18^
**As**	**a:** 4.4^17^ 4.14^96^ 7.7^89^ 3.6 **b:** 10^17^ 10.9^89^ 3.7^90^ **nd:** 4^16^	**a:** 96^76^* 97^17^ 86.4^38^* 45^96^* 110.36^89^ 98.7^90^ 83^101^* 88^101^*	**a: ** 0.0047 μg/g^76^ 0.000037 μg/mL^17^ (IDL) 0.000043 μg/mL^17^ (MDL) 0.016 μg/g^18^ 0.002 μg/g^38^ 0.0004 μg/g^100^ 0.0003 μg/g^101^ **b:** 1.0 μg/mL^16^ 0.00001 μg/mL^90^ 0.00005 μg/mL^8^ **nd:** 0.0009148 μg/mL^96^
**Se**	**a: ** 1.8^17^ 5.47^96^ 9.0^89^ 2.1^90^ **b:** 2.9^17^ 11.2^89^ 3.0^90^ **nd:** 30^35^ 10^100^ 6.4^100^	**a: ** 95^76^* 119^17^ 81.4^38^* 69^96^* 119.15^89^ 101.5^90^ 125^101^* 64^100^* 62^100^*	**a: ** 0.017 μg/g^76^ 0.000062 μg/mL^17^ (IDL) 0.000094 μg/mL^17^ (MDL) 0.12 μg/g^18^ 0.04 μg/g^38^ 0.0015 μg/g^101^ **b:** 0.00004 μg/mL^90^ **nd:** 0.0003438 μg/mL^96^
**Br**			**a: ** 2.168 μg/g^18^
**Rb**	**a: ** 2.8^89^ **b:** 9.6^89^ **nd:** 1.4–2.7^13^ 4^16^ 0.7^35^	**a: ** 1.01^13^ 103.3^38^* 106^39^* **b:** 104.06^89^	**a: ** 0.000012 μg/mL^13^ 0.021 μg/g^18^ 0.002 μg/g^38^ **b:** 1.0 μg/mL^16^
**Sr**	**a:** 2.6^17^ 1.0^96^ 2.4^89^ **b:** 4.6^17^ 7.9^89^ **nd:** 6.9–16.8^13^ 8^16^ 0.4^35^ 2.9^37^	**a:** 125^13^ 110^17^ 99.1^38^* 135^96^* **b:** 92.03^89^ 98^37^*	**a:** 0.000017 μg/mL^13^ 0.000014 μg/mL^17^ (IDL) 0.000037 μg/mL^17^ (MDL) 0.036 μg/g^18^ 0.05 μg/g^38^ 0.00001 μg/mL^37^ **b:** 1.0 μg/mL^16^ **nd:** 0.0000190 μg/mL^96^
**Y**	**nd:** 12.1^119^		**a:** 0.0006 μg/g^18^ **nd:** 0.0000012 μg/mL^119^
**Zr**	**a:** 1.7–4.2^102^ **b:** 2.0–6.1^102^ **nd:** 10^35^ 3.0^37^	**b:** 0.1–7.2^102^^#^ 91.0 -118^102^ 106^37^*	**a:** 0.000055 μg/mL^102^ 0.000002 μg/mL^37^ 0.008 μg/g^18^
**Nb**			**a:** 0.0005 μg/g^18^
**Mo**	**a:** 4.25^96^ 3.2^89^ 3.5^90^ **b:** 8.8^89^ 7.9^90^ **nd:** 1.1–3.0^13^ 5^35^ 2.8^37^ 2.9^119^	**a:** 102^76^* 102–107^13^ 95^96^* 102^37^* 108.7^90^ 110^119^ 105.49^89^ **b:** 96^101^* 104^37^*	**a:** 0.0057 μg/g^76^ 0.000008 μg/mL^13^ 0.01 μg/g^18^ 0.006 μg/g^38^ 0.0048 μg/g^101^ 0.000005 μg/mL^37^ **b:** 0.000025 μg/mL^90^ **nd:** 0.0000194 μg/mL^96^ 0.0000012 μg/mL^119^
**Ru**			**a:** 0.017 μg/g^18^
**Pd**			**a:** 0.002 μg/g^18^
**Ag**	**nd:** 8^35^		**nd:** 0.00013 μg/g^98^
**Cd**	**a:** 6.1^17^ 1.96^96^ 4.1^89^ 5.8^90^ **b:** 10.0^17^ 9.3^89^ 6.3^90^ **nd:** 1.1–2.6^13^ 6.6^95^ 4.2^95^ 4.6^14^ 9.5^14^ 0.3^119^	**a:** 106^76^* 94–114^13^ 83^17^ 99^95^* 96^95^* 91.6^38^* 40^96^* 94.73^89^ 98.8^90^ 93^14^ 101^14^ 120^101^* 95^101^* 111^119^ **b:** 102 ± 7^95^ 102 ± 3^95^	**a:** 0.0029 μg/g^76^ 0.000001 μg/mL^13^ 0.0000088 μg/mL^17^ (IDL) 0.000013 μg/mL^17^ (MDL) 0.00006 μg/g^95^ (0.017 μg/mL) 0.002 μg/g^18^ 0.001 μg/g^38^ 0.00008 μg/g^100^ 0.0001 μg/g^101^ 0.0001 μg/mL^14^ **b:** 0.000001 μg/mL^90^ **nd:** 0.0000032 μg/mL^96^ 0.00047 μg/g^98^ 0.0000006 μg/mL^119^
**In**			**a:** 0.0002 μg/g^18^
**Sn**	**a:** 0.57^96^ **nd:** 30^35^ 5.2^37^	**a:** 73.3^38^* **b:** 104^37^*	**a:** 0.004 μg/g^18^ 0.04 μg/g^38^ 0.00003 μg/mL^37^ 0.0001 μg/g^101^ **nd:** 0.0000139 μg/mL^96^
**Sb**	**a:** 2.8^17^ 0.85^96^ **b:** 3.6^17^ **nd:** 10^35^ 2.3^37^	**a:** 104^17^ 90.0^38^* **b:** 96^37^*	**a:** 0.000017 μg/mL^17^ (IDL) 0.000028 μg/mL^17^ (MDL) 0.002 μg/g^18^ 0.0006 μg/g^38^ 0.000002 μg/mL^37^ **nd:** 0.0000193 μg/mL^96^
**Te**			**a:** 0.006 μg/g^18^
**I**	**a:** 3.1^90^ **b:** 4.7^90^	**a:** 98.7^38^* 94.5^90^	**b:** 0.00010 μg/mL^90^
**Cs**	**nd:** 2.9–4.3^13^ 20^35^		**a:** 0.000001 μg/mL^13^ 0.0009 μg/g^18^
**Ba**	**a:** 4.4^17^ 1.19^96^ **b:** 6.0^17^ **nd:** 4^16^ 3^35^ 4.7^37^ 1.5^119^	**a:** 110^17^ 92^96^* **b:** 92^37^*	**a:** 0.000011 μg/g^17^ (IDL) 0.00038 μg/mL^17^ (MDL) 0.025 μg/g^18^ 0.03 μg/g^38^ 0.0002 μg/g^100^ 0.00001 μg/mL^37^ **b:** 0.5 μg/mL^16^ **nd:** 0.0011218 μg/mL^96^ 0.0000014 μg/mL^119^
**Ce**		**a:** 82.5^38^*	**a:** 0.0008 μg/g^18^ 0.002 μg/g^38^
**Sm**			**a:** 0.002 μg/g^18^
**Gd**			**a:** 0.001 μg/g^18^
**Tm**			**a:** 0.0006 μg/g^18^
**Hf**			**a:** 0.0002 μg/g^18^
**Ta**			**a:** 0.002 μg/g^18^
**W**	**nd:** 9^35^ 4.2^37^	**b:** 98^37^*	**a:** 0.000001 μg/mL^37^ 0.074 μg/g^18^
**Re**			**a:** 0.0002 μg/g^18^
**Os**			**a:** 0.013 μg/g^18^
**Pt**			**a:** 0.0005 μg/g^18^
**Au**			**a:** 0.001 μg/g^18^
**Hg**		**a:** 100^76^* 84.1^38^* 97^101^* 74^101^*	**a:** 0.0011 μg/g^76^ 0.037 μg/g^18^ 0.007 μg/g^38^ 0.0002 μg/g^101^
**Tl**	**nd:** 3.4^37^	**a:** 99^37^* **b:** 94^37^*	**a:** 0.0005 μg/g^18^ 0.000001 μg/mL^37^ 0.00004 μg/g^100^
**Pb**	**a:** 5.2^17^ 1.28^96^ 4.8^89^ **b:** 8.3^17^ 8.6^89^ **nd:** 10.0–10.5^13^	**a:** 96^76^* 109–112^13^ 125^17^ 94.3^38^* 93^39^* 86^101^* **b:** 92.85^89^	**a:** 0.0079 μg/g^76^ 0.00001 μg/mL^13^ 0.000099 μg/mL^17^ (IDL) 0.00016 μg/mL^17^ (MDL) 0.002 μg/g^18^ 0.005 μg/g^38^ 0.0009 μg/g^100^ 0.0013 μg/g^101^ **nd:** 0.0000414 μg/mL^96^ 0.00036 μg/g^98^ 0.000004 μg/mL^119^
**Bi**	**nd:** 4.3^37^	**a:** 102^37^* **b:** 110^37^*	**a:** 0.000001 μg/mL^37^ 0.002 μg/g^18^ 0.004 μg/g^38^
**U**	**a:** 2.3^17^ **b:** 3.1^17^	**a:** 111^17^ 106.1^38^*	**a:** 0.00000029 μg/mL^17^ (IDL) 0.00000045 μg/mL^17^ (MDL) 0.0002 μg/g^38^ 0.00005 μg/g^100^

*nd* method of parameter determination was not defined;

^#^ Trueness expressed as a Bias;

*Calculated based on the literature data.

Unfortunately, especially in the case of precision and limit of detection, a clear information on the procedure for calculating a validation parameter is not provided by the authors. Very often only its value is indicated. Furthermore, the measurement conditions (i.e. the number of repetitions) applied in the analysed research work are very different. Therefore, the prepared summary is intended to provide a general view of the analytical capabilities of selected instrumental techniques in studying the elemental composition of biomedical samples based on its validation results.

The focus was on the discussion of validation parameters obtained for selected elements, which are very often the subject of analysis in case of biomedical samples due to their significant importance for the proper functioning of living organisms. These include P, S, K, Ca, Fe, Cu, Zn and Se.

The data concerning P found for ICP-OES and ICP-MS show that both techniques are characterized by very good precision values. In case of determination of P using ICP-OES, the precision is in the range from 1% (calculated as RSD of mean value in five independent measurements of pork liver and bovine liver reference materials)^13^ to 3% (calculated as RSD from 3 independent measurements of human bone marrow fluid sample)^35^. In turn, for ICP-MS, one can see strong dependence of precision values from the matrix of studied sample. In the work of Takasaki et al. the precision of P concentration measurement for the reference material (NIST SRM 1577b bovine liver) is 0.9%, while for the sample of *E. coli* cells is 3.9%^119^. Analysis of P content carried out using ICP-OES and ICP-MS is characterized by a high value of the trueness of the obtained results. The best value of trueness, equal to 99.8%, was obtained in the work of Zaichick et al. who were studying using the ICP-OES the correlation between elemental composition of bones and age/sex of people. Trueness was calculated there based on the measurement of the reference material (SRM NIST 1486 Bone Meal)^84^. The values of limit of detection obtained for P using both discussed techniques are satisfactory. The lowest LOD for this element (found based on referenced papers) is 0.000012 µg/ml and it was obtained by Takasaki et al. using ICP-MS. The authors conducted a multielemental analysis of a very small amount of analyte (20 µl) using a highly effective sample introduction system. Compared to the conventional technique, requiring about 2 ml of sample, they achieved a significant improvement in the absolute detection limit value (from 28 to 0.2 pg) for P^119^. Based on the results for real biomedical samples (human cerebrospinal fluid) Korvela et al. received a LOD for P equals to 2 µg/ml^16^.

In the work of Takasaki et al. the precision of S measurement performed using ICP-MS depended on the sample type. For the reference material (NIST SRM 1577b bovine liver) it was 0.8%, while for the sample of *E. coli* cells was 3.6%^119^. In this study also trueness was determined and equaled 100%^119^. The detection limit of S obtained using ICP-MS was two order of magnitude smaller than that in the ICP-OES technique and these values equaled 0.0003 µg/ml^119^ and 0.035 µg/ml^78^, respectively.

The precision values obtained for K using ICP-OES varied from 0.6%^35^ to 4.7%^13^. As in the case of P and S, the precision of K measurements carried out using ICP-MS depended on the matrix and was equal to 1.2% for reference material and to 3.1% for *E. coli* cells^119^. The best trueness of the obtained results of K concentration, amounting to 98.7%, was obtained in the work of Boulyga et al. which regarded the multielemental analysis of small amounts of pathologically changed thyroid tissue carried out using the ICP-MS. Trueness was tested based on measurements of the reference material NIST SRM 1566a (oyster tissue). The LOD of K obtained by Boulyga et al. for thyroid samples was 3 µg/g^38^. In turn, this determined by Korvela et al. for cerebrospinal fluid using the ICP-OES was 5.9 µg/ml^16^. The lowest detection limit of the element, similarly as for P and S, was obtained in the work of Zaichack et al. using ICP-MS and it was equal to 0.01 µg/ml^84^.

In presented examples of Ca determination in biological samples, the ICP-OES, ICP-MS, and F-AAS were used for elemental analysis. The accuracy of the results obtained using the ICP-OES ranged from 2% (calculated as RSD of 3 measurements of human bone marrow-fluid)^35^ to 6% (determined as RSD of mean values of Ca content in human cerebrospinal fluid)^16^. The values of precision for Ca determinations using the ICP-MS was similar and did not exceed 9.5%. The best value for this validation parameter (1.2%), was obtained in the work of Meyer et al. as an intra-day precision (three samples of human serum were separately digested on one day and measured)^90^. Determination of Ca concentration in biomedical samples using all of the three mentioned techniques is generally characterized by a very good value of trueness which usually falls within the range from 93%^119^ to 105%^39, 69^. The best trueness for Ca determination (100%) was obtained in the work of Krebs et al. It was calculated based on the comparison of the certified and the measured value of this element concentration in the certified material NIST RM 8414 (bovine muscle) using ICP-MS^9^. The lowest detection limit for Ca (0.00001 µg/ml) was obtained in the work of Leblondel et al. who have studied the distribution of elements in rat tissues using ICP-OES^78^. In turn, in the work of Yang et al. a limit of detection of Ca obtained for sample of rabbit artery using ICP-OES was 0.005 µg/g (calculated as 3*SD from 16 measurements)^69^.

Fe is very often the subject of studies carried out using the F-AAS, GF-AAS, ICP-OES and ICP-MS. In general, it can be stated that for techniques using plasma for analytes determination the obtained values of precision are very good and do not exceed 4.1%. The highest value of this parameter in case of Fe determination (25%) was contained in the work of Sahuquilo et al. concerning the elemental analysis of human gallstones using the ICP-OES. It was pointed out that this may be caused by the low content of this element in the analysed sample (pure cholesterol gallstone sample)^25^. For all discussed techniques of Fe determination very good results of trueness were obtained, calculated both with spike sample methods and based on measurements of reference materials. The highest agreement between the measured and certified values of Fe concentration was obtained using the ICP-OES in the works of Rahil-Khazen et al. (100.3% for human hair reference material GBW 09101 measurements)^3^ and Yang et al. (99.7% for NIST bovine liver standard reference material measurements)^69^. The best value of Fe recovery for the real sample matrix was obtained with the use of F-AAS (97.8%) in the work of Fakayode et al., where the contents of trace elements in human hair samples were examined^34^. Based on the analysed papers it can be said that the lowest detection limit for Fe is 0.0001827 µg/ml and was obtained by Xie et al. during measurements of standard solution using ICP-MS^96^. For the same technique, the value of Fe detection limit determined based on the measurements of real sample of human serum was 0.00205 µg/ml in the work of Meyer et al.^90^.

All techniques selected for the discussion were used to determine the content of Cu in biomedical samples. Performed measurements are characterized by good precision values, ranging from 0.6% (within-run precision calculated based on 20 measurements of a digested human serum sample using ICP-OES)^83^ to 11.1% (reproducibility determined based on 6 measurements, done with the use of ICP-OES, of Cu concentration in the solution of digested tooth samples)^26^. In most cases, the trueness obtained for Cu ranges from 81% (ICP-MS)^100^ to 108% (ICP-OES)^86^. Also, the values of recovery parameter are satisfactory for these techniques and range from 95% (ICP-OES)^81^ to 104.1% (F-AAS)^34^. Both the best and the worst agreement between measured and certified Cu concentration values were obtained in the work of Garcia-Vaquero et al. The trueness of 100% was obtained when measuring DORM-3 CRM (fish protein), whilst 125% when analysing CRM 186 (pig kidney)^101^. The lowest LOD for Cu was obtained in the work of Afridi et al. based on blank solution GF-AAS measurements and this value is 0.00000017 µg/g^60^. Analysing the real sample (human serum) and using for this purpose ICP-MS, Meyer et al. obtained LOD for Cu equal to 0.0001 µg/ml^90^.

Zn is an element frequently examined in biomedical samples. The precision of Zn measurements performed using ICP techniques is satisfactory for most of the discussed papers and ranges from 0.3% (ICP-OES)^83^ to 8.7% (ICP-MS)^89^. In the work of Panayi et al., where Cd and Zn contents in brain tissue were measured using ICP-MS, the precision of Zn determination was 22.8% and 16.3% depending on the type of tissue examined^95^. Observed higher values of this parameter in case of results obtained for real samples reflect possible non-homogeneity in their elemental composition. Also the trueness of the results obtained using the discussed techniques can be considered as satisfactory. When comparing certified and measured values, the trueness ranges from 87% (measurements of standard reference material CRM 1577-bovine liver using ICP-OES)^79^ to 116% (calculated based on measurements of standard tissue of bovine muscle with the use of ICP-MS)^9^. The obtained recovery values range from 87% (2 µg of the analyte added to rat liver samples and reanalysed using ICP-OES)^79^ to 111% (analysed samples spiked with a known amount of an element and measured with the use of ICP-MS)^95^. The lowest LOD for Zn is 0.000017 µg/ml and was determined using the ICP-MS by Takahashi et al. It was calculated as the concentration of the element for which the number of counts is three times higher than the standard deviation of the background counts^13^. The lowest LOD of Zn for the matrix constituting the biomedical sample was 0.00024 µg/ml and was calculated based on results obtained for human serum using the ICP-MS^90^.

The presented validation parameters regarding the determination of Se in biomedical samples refer to the use of the ICP-OES and ICP-MS. In most cases, obtained results are characterized by very good precision from 1.8% (expressed as repeatability calculated from 6 measurements of CRM Seronorm Trace Elements Serum L-2 carried out on the same day)^17^ to 11.2% (inter-day precision expressed as the relative standard deviation of 10 measurements of CRM Clinchek Blood Plasma Control level 1)^89^. The highest value of the precision for Se found in discussed papers, equals to 30%, was obtained by Hasegawa et al. who have studied the elemental composition of human bone-marrow fluid^35^. The parameter was calculated as RSD of 3 replicated measurements of the sample done using ICP-MS. The values of the trueness in case of Se determination ranged from 62 to 125% and depended on the applied analytical technique. The best agreement between the measured and certified Se concentrations, equals to 101.5%, was obtained in the work of Meyer et al. for human blood serum reference material with the use of ICP-MS^90^. The lowest LOD for Se, achieved also by Meyer et al., was equal to 0.00004 µg/ml for human serum samples^90^.

## Evaluation of the usefulness of the total reflection X-ray fluorescence (TXRF) for the elemental analysis of animal tissues

The total reflection X-ray fluorescence is a technique of quantitative elemental analysis and it is based on the registration of fluorescent radiation emitted by atoms excited as a result of ionization of their inner shell. The fundamental principle of TXRF is application of measurement geometry, which allows for the occurrence of total X-ray reflection phenomena. Primary, X-ray radiation is used for atoms ionization and it falls on a sample surface at very small glancing angle and is totally reflected from a sample carrier. Thus allows for double excitation of atoms, first by primary, incident beam and then by reflected beam. The energy of emitted fluorescent radiation is characteristic for each element and depends on the atomic number. Therefore, the measurement of the fluorescent radiation energy, allows identifying elements present in the sample^121^.

**Figure 5:**
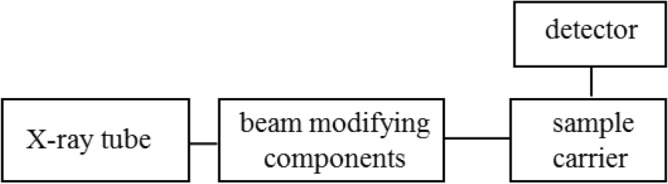
Block diagram of a typical instrument used for TXRF.

A layout of the experimental setup used for TXRF measurements is presented in Fig. 5. X-ray tubes with molybdenum anode are most often used as X-ray sources for the TXRF. The spectrum of the beam generated in the lamp is continuous and requires monochromatization to reduce background radiation. Therefore, the beam passes through the set of modifying components, like shut-off reflectors and crystalline or multilayer monochromators. The most commonly used detectors for TXRF are semiconductor detectors (mainly Silicon Drift Detector or Si(Li)). The detector, sample carrier and source of radiation are arranged at angle 90°, ant that geometry allows for a significant decrease of the background of the recorded spectrum To improve the detection of low-energy characterisctic radiation, if possible, the analysis should be conducted in a vacuum to reduce the absorption of radiation in the air. It is also important to provide a suitably thin layer of the sample, which also affects the elimination of radiation absorption effects^122^.

The TXRF is widely used in the analysis of biomedical samples, both solid and liquid^123,124^. For liquid samples, such as body fluids, the measurement can be carried out directly^111,125,126^ or after appropriate dilution of the sample^107,127^. Solid samples, both soft (such as organ tissues^106,128^) and hard (e.g. bones^6^) require sample preparation. Its purpose is to conduct the sample into the liquid form and to remove the organic matrix, which could contribute to the increase of scattered radiation and worsening of the detection limits of the elements.

Ostachowicz et al. used the TXRF to determine concentrations of Na, Mg, Cl, K, Ca, Cu, Zn and Br in serum and cerebrospinal fluid taken from patients suffering from amyotrophic lateral sclerosis and healthy people constituting a control group^111^. Margui et al. have performed a multi-elemental analysis of whole blood taken from patients with thyroid diseases. For this purpose, they examined the influence of different dilution procedures and sample volume deposited on the reflector on the carried elemental analysis^107^. Sanchez et al. have analysed oral fluids of women with osteoporosis and osteopenia in terms of P, S, Cl, K, Ca, Cr, Fe, Ni, Cu and Zn contents^129^. Martinez et al. have determined the concentration of Fe in the blood of people living in the Mexico Valley metropolitan zone. Before analysis, Fe was extracted from the samples, which allowed to reduce the detection limit of the element and the obtained results were compared with the levels of Fe in environmental samples^124^. Majewska et al. have used the TXRF to determine the reference values of K, Ca, Ce, Mn, Fe, Ni, Cu, Zn, Br, Rb and Sr in human urine^104^. Another study concerned the determination of gadolinium content in urine and blood plasma using the TXRF. The same samples were also measured using ICP-MS in order to perform a comparison with results obtained by TXRF. Concentrations of Gd determined by both techniques showed very good correlations^125^. It was found that TXRF can be considered as a simple technique for routine analysis of gadolinium levels in body fluids in clinical laboratories^125^. Abraham et al. have analysed the concentration of Ti, V and Al in oral fluids taken from patients with dental implants. The study was performed in order to explore the possibility of corrosion effect of metals in implanted materials. As an X-ray source for the TXRF, they used synchrotron radiation (SR-TXRF, Total Reflection X-ray fluorescence with Synchrotron Radiation). The use of a linearly polarized synchrotron radiation in TXRF reduces the influence of scattered radiation on the recorded spectrum. High intensity and monochromatization of the exciting synchrotron beam lead to further improvement of the detection limits of analysed elements^126^.

Wagner et al. have performed a multielemental analysis of muscle and liver fish tissues. The objective of their study was investigation of the influence of environmental pollution caused by the local coal industry on the fish tissues and find out if it may stands a potential danger for the consumers^41^. Marco et al. have analysed the contents of Cu and Zn in human brain and Fe, Cu, Zn, Se and Pt in human serum. Different procedures of sample pretreatment, including digestion and preparing slurries, as well as methods of standardization were investigated^130^. Serpa et al. have studied the content of Al, P, S, Cl, K, Ca, Ti, Fe, Cu, Zn, Br and Rb in different parts of rat brain. They indicated how the concentrations of the elements change with the age of the animal^106^. In turn, Varga et al. analysed content of Cr, Mn, Fe, Ni, Cu, Zn, Rb and Pb in the samples of liver biopsy taken from patients suffered from chronic diffuse liver disease^39^. There are many studies where TXRF is used for the comparison of elemental composition of cancerous and healthy tissues. Such studies were done for colon^131^, breast^22,109,131^, stomach^131^, uterus^132^, prostate^128^, intestine^36^ and the corresponding healthy tissues of these organs. Other investigation concerned the elemental analysis of brain tumours of various types and grades^133^ as well as breast, lung and intestinal cancer tissues^134^. Using the TXRF, attempts were also made to find correlations between the concentrations of trace elements in samples of cancerous and benign tissues of the rectum, colon, thyroid, kidneys, larynx and lung^134^. Czarnowski et al. have analysed the distribution of trace elements in normal and cancerous tissues of the human stomach, colon and rectum. The frozen samples were not digested but were cut in a microtome and directly put on the reflector^135^. Using the TXRF, the concentrations of elements (P, S, K, Ca, Fe, Ni, Zn, Cu, Br) were compared in cancerous and healthy breast tissues. Several fragments were taken from each tissue to determine the variability of elemental distribution in the obtained samples^123^. The technique has also been successfully used to determine the platinum content in biological samples. These were the tissues collected during biopsy of patients subjected to Cisplatin chemotherapy at different times from its administration. The obtained detection limit of platinum was 0.1 ppm^136^. Due to the TXRF analysis speed, uncomplicated measurement process and accuracy, this technique was considered suitable for carrying out trace analyses of small amounts of samples in medical and clinical trials^136^.

Carvalho et al. have analysed distribution of Ca, Mn, Fe, Cu, Zn, Sr, Ba and Pb in human femur originated from archaeological excavations^6^. Rodriguez et al. have investigated whether the dog hair may be considered as potential biomarker of environmental arsenic exposure. Samples were measured using TXRF and the ICP-OES to check the accuracy of proposed methodology and there were no significant differences between the results obtained with these two techniques^42^.

### Experimental animals and sample preparation

The subject of the study was a group of six male Wistar rats, which were originated from the colony of the Department of Neuroanatomy, Institute of Zoology and Biomedical Research, Jagiellonian University, Krakow. The animals constituted a control group in our previous experiment focused on the elemental anomalies occurring in rat organs after the systemic exposure to iron oxide nanoparticles^7^.

All procedures in which animals were involved were carried out with the approval of the Bioethical Commission of the Jagiellonian University (agreement no. 121/2015) and were performed in accordance with the international standards. The rats were housed in cages, with the access to water and standard rodent diet ad libitum. On the 60th day of their postnatal development, the animals were euthanized and perfused with 0.9% saline of high analytical purity. Muscle, brain, kidney, liver, heart and spleen were excised from the bodies, weighted and quickly frozen in liquid nitrogen. Removed organs, packed in sterile Whirl-pack bags, were stored in temperature − 80 °C.

For elemental analysis of rat organs the TXRF was applied. Before measurements, the tissues were weighted and digested. The organ weights were in range of 0.417–0.610 g for muscle, 1.722–1.923 g for brain, 2.126–2.806 g for kidney, 8.713–14.880 g for liver, 0.805–1.116 g for heart and 0.498–0.773 g for spleen. The removed livers, compared to the other organs, were significantly larger in weight. Therefore, each individual liver was cut into 5 or 6 separate pieces and separately subjected to mineralization. Digestion process was performed in few steps and the maximum achieved temperature was equal 190 °C at pressure 30 bar. Each organ was placed in a separate teflon vessel adding high purity 65% nitric acid (100,441/Suprapur, Merck Group). The typical volume of nitric acid was about 2.5 ml per 1 g of tissue. Microwave-assisted digestion was performed with the use of mineralizer SpeedWave 4 (Berghof). The conditions of the mineralization process were chosen according to the recommendations of the mineralizer manufacturer. After digestion, the teflon vessels were cooled down and their contents were separately poured to the test tubes. The samples were stored at a low temperature until analysis.

### Basis of quantitative elemental analysis

The quantitative analysis was carried out with the use of the internal standard method. For this purpose gallium solution at concentration of 10 ppm was used. Typically, 0.3 ml of such solution was added to 1 ml of the digested sample and the content of the test tube was thoroughly mixed using a laboratory shaker to ensure the homogeneity of the solution. Afterwards, 2 µl of sample solution was transferred onto quartz glass carrier (Bruker Nano) and dried on a heating plate. Before this procedure the sample carriers were cleaned according to the producer recommendations and tested through the control measurements of background.

Analysis of the element contents in the liquid sample, using the internal standard method, is based on the relation (1):

1 $${C}_{i}=\frac{{C}_{IS}*{N}_{i}}{{N}_{IS}*{S}_{i}}$$

where, ${C}_{i}$ concentration of the element *i* in the liquid sample [ppm], ${C}_{IS}$ known concentration of the internal standard [ppm], ${N}_{i}$ and ${N}_{IS}$ the numbers of counts for the element *i* and for the internal standard, ${S}_{i}$ relative sensitivity for the element *i* determined by the analysis of calibration standards solutions.

In order to calculate the content of the element in the liquid sample of tissue, the dilution resulting from the addition of the internal standard has to be taken into account. The concentration of the element *i* in the liquid sample of the tissue is, then, expressed by the formula (2):

2 $${C}_{i}^{s}=d*{C}_{i}$$

where, $d$ coefficient of the dilution equal to the sum of the volume of the liquid sample and the volume of the solution of the internal standard.

To determine the concentration of the element in the organ, the mass conversion factor ${k}_{n}$ for the organ *n* should also be taken into account which is expressed by the Eq. (3):

3 $${k}_{n}=\frac{{m}_{n}+{m}_{a}}{{m}_{n}}$$

where, ${m}_{n}$ mass of the organ *n*, ${m}_{a}$ mass of the nitric acid added during mineralization.

As a result, the concentration ${C}_{i}^{n}$ of the element *i* in the organ *n* is calculated based on the dependence (4):

4 $${C}_{i}^{n}={C}_{i}^{s}*k.$$

### Evaluation of validation parameters

Limit of detection ${LOD}_{ij}$ [ppm] for the element *i* in the organ *j* was calculated based on the results obtained for six samples of each organ in accordance with the formula (5):

5 $${LOD}_{ij}=\frac{3*{C}_{ij}*\sqrt{{N}_{BG}}}{{N}_{ij}}$$

where, ${C}_{ij}$ concentration of the element *i* in the organ *j* [ppm], ${N}_{BG}$ area of the background under ${K}_{\alpha }$ line for the element *i* in the organ *j* [a.u.], ${N}_{ij}$ area of the peak for ${K}_{\alpha }$ line of the element *i* in the organ *j* [a.u.].

Final values of the detection limits of the examined elements for each organ were calculated as an average of the results obtained for six analysed samples.

Both precision and trueness of the technique were determined using standard reference material IAEA-A-13—the freeze dried animal blood. Six SRM samples weighing 200 mg were prepared, placed in separate teflon vessels with 5 ml of high purity 65% nitric acid and digested. Before elemental analysis of the reference material, 0.3 ml of the solution of the internal standard was added to each 1 ml of the sample. Similarly, as in the case of animal samples, the solution contained Ga at concentration of 10 ppm. For TXRF analysis 2 µl of sample solution was deposited on quartz glass pad (Bruker Nano) and dried on a heating plate. In order to determine the repeatability, first the coefficient of variation ${CV}^{i}$ for the element *i* was determined in each measurement series as:

6 $${CV}^{i}=\frac{1}{{\stackrel{-}{x}}^{i}}*\sqrt{\frac{\sum_{j=1}^{n}{\left({x}_{j}^{i}-{\stackrel{-}{x}}^{i}\right)}^{2}}{n-1}}*100\%$$

where, ${\stackrel{-}{x}}^{i}$ the average concentration of the element *i* in a given measurement series [ppm], ${x}_{j}^{i}$ the concentration of the element *i* obtained for single measurement *j* [ppm], $n$ number of measurements in single series.

Repeatability of the analytical technique, express as coefficient of variation ${CV}_{g}^{i}$ and taking into account all measurement series for each element *i* is defined as:

7 $${CV}_{g}^{i}=\sqrt{\frac{1}{k}\sum_{j=1}^{k}{\left({CV}_{j}^{i}\right)}^{2}}$$

where, $k$ the number of measurement series.

Trueness for used analytical technique was calculated for every element according to the formula (8):

8 $$T=\frac{{\stackrel{-}{a}}^{i}}{{a}_{ref}^{i}}*100\%$$

where, ${\stackrel{-}{a}}^{i}$ the average concentration of the element *i* calculated for all measurement series, ${a}_{ref}^{i}$ the reference value of the concentration of the element *i* in IAEA-A-13 SRM.

### Instrument and measurements conditions

The measurements were carried out in the Laboratory of X-ray Methods of the Institute of Physics at the Jan Kochanowski University in Kielce. For this purpose, S2 PICOFOX (Bruker Nano) TXRF spectrometer was used. The instrument is equipped with an air cooled X-ray tube with molybdenum anode and multilayer monochromator. The energy of the exciting beam was 17.5 keV and the focal spot was equal to 1.2 × 0.1 mm^2^.

Samples on the quartz glass carriers were placed in 25-position spectrometer cassette. The measurement time was 20 min per sample and each of six SRM samples were measured 10 times.

### Elemental composition of the normal rat organs

**Figure 6:**
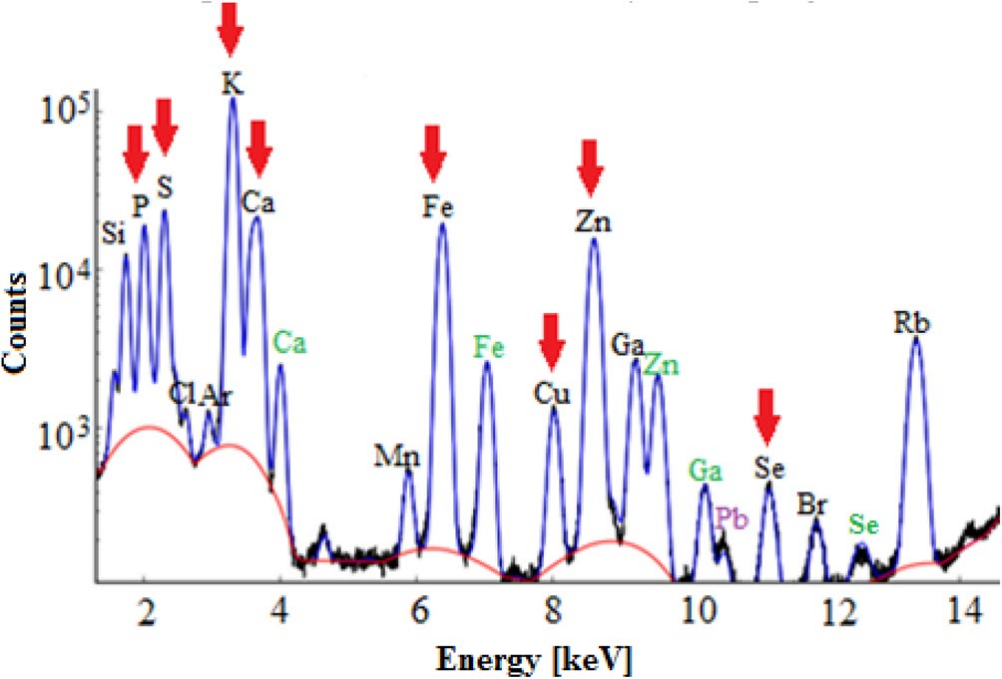
An example of X-ray fluorescence spectrum registered for liver sample. Kα, Kβ and L spectral lines were marked with black, green and purple colours, respectively. Lines of the analysed elements were marked with red arrows.

Energy calibration of the obtained TXRF spectra, carried out in the PyMCA program, allowed us to identify the elements present in the examined tissues. As an example, the spectrum of selected liver sample is shown in the Fig. 6. The identification and quantification of elements was performed based on their ${K}_{\alpha }$ lines and the following elements: P, S, K, Ca, Fe, Cu, Zn and Se were the subject of further quantitative study. As one can notice, Si line was also present in the spectrum, the source of which was the used quartz sample carrier, the Ar line from the air and Ga, which was added to the sample as an internal standard.

**Figure 7:**
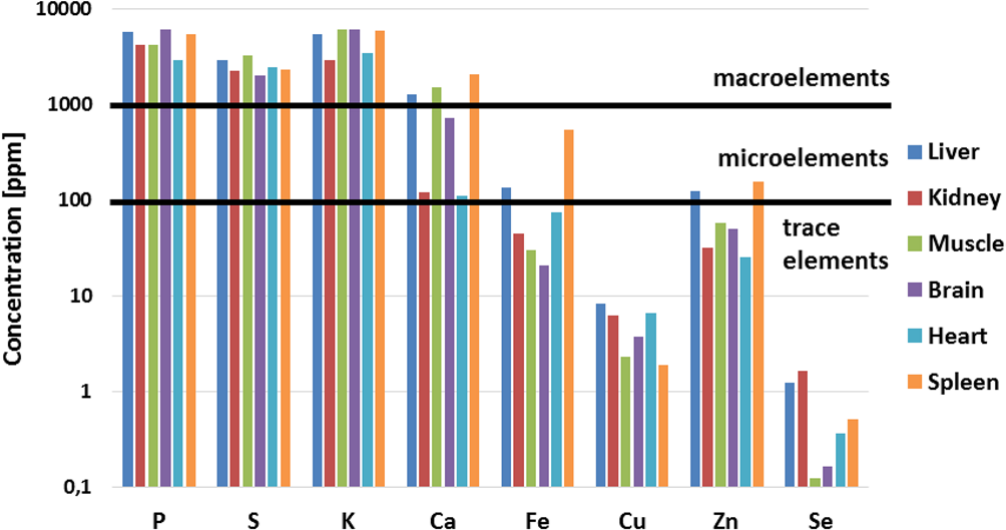
The contents of elements in every organ determined using TXRF.

The quantitative analysis, performed on the way described earlier, allowed us to obtain information about the concentrations of P, S, K, Ca, Fe, Cu, Zn and Se in the examined organs of normal rats and the obtained data were used to prepare Fig. 7 and placed in the Table 5 which contains also the comparison of the recorded concentrations with the available literature data from various techniques of elemental analysis.

**Table 5:** The median value [μg/g] of P, S, K, Ca, Fe, Cu, Zn and Se concentrations obtained for each organ using TXRF together with corresponding literature data. In the parentheses the interquartile range of the concentration value is shown.

Element	Liver		Kidney		Muscle		Brain		Heart		Spleen	
	**Measured by TXRF**	**Literature data**	**Measured by TXRF**	**Literature data**	**Measured by TXRF**	**Literature data**	**Measured by TXRF**	**Literature data**	**Measured by TXRF**	**Literature data**	**Measured by TXRF**	**Literature data**
**P**	5750 (704)	ICP-OES 2983.44 ± 163.37^78^ 3900 ± 400^103^ 3700^103^	4250 (280)	ICP-OES 3300 ± 20^28^ 3400^28^ 2531.77 ± 167.09^78^	4270 (120)	ICP-OES 1971.29 ± 121.21^78^	6110 (390)	ICP-OES 2521.85 ± 99.51^78^	2970 (270)	ICP-OES 2129.39 ± 94.55^78^	5460 (860)	ICP-OES 3188.04 ± 241.8^78^
**S**	2980 (150)	ICP-OES 2346.88 ± 202.56^78^	2290 (150)	ICP-OES 2327.04 ± 210.56^78^	3260 (150)	ICP-OES 2417.92 ± 194.24^78^	2030 (160)	ICP-OES 1320.64 ± 73.28^78^	2490 (310)	ICP-OES 2524.16 ± 113.92^78^	2370 (460)	ICP-OES 1953.6 ± 122.88^78^
**K**	5440 (610)	ICP-OES 3197.22 ± 152.88^78^ 3300 ± 300^103^ 3000^103^	2920 (200)	ICP-OES 2600 ± 20^28^ 2600^28^ 2467.14 ± 166.14^78^	6110 (380)	ICP-OES 3460.47 ± 231.66^78^	6100 (520)	ICP-OES 3084.51 ± 180.96^78^	3470 (180)	ICP-OES 2760.81 ± 157.95^78^	5920 (1060)	ICP-OES 4034.16 ± 253.5^78^
**Ca**	1300 (630)	ICP-OES 30.28 ± 2.48^78^ 36.6 ± 4.3^103^ 37.5^103^	120 (210)	ICP-OES 81 ± 9^28^ 104^28^ 54.52 ± 5.08^78^	1530 (3300)	ICP-OES 41.12 ± 3.48^78^	730 (1540)	ICP-OES 33.04 ± 3.52^78^	112 (320)	ICP-OES 25.72 ± 2.16^78^	2070 (6060)	ICP-OES 32.92 ± 5^78^
**Fe**	137 (17)	ICP-OES 531.0 ± 18.0^79^ 1023.5 ± 95.9^79^ 71.064 ± 8.43^78^ 598 ± 102^103^ 643^103^ ASA: 73.4 ± 10.4^137^	45.3 (4.9)	ICP-OES 361.2 ± 9.4^79^ 416.3 ± 97.1^79^ 170 ± 22^28^ 267^28^ 40.88 ± 5.08^78^	30.1 (4.3)	ICP-OES 9.52 ± 2.07^78^	20.6 (4.4)	ICP-OES 11.48 ± 0.95^78^	75 (13)	ICP-OES 643.1 ± 31.3^79^ 454.2 ± 9.4^79^ 57.12 ± 6.26^78^ ASA: 62.4 ± 6.0^137^	547 (200)	ICP-OES 185.19 ± 41.61^78^ ASA: 173.3 ± 32.2^137^
**Cu**	8.37 (0.78)	ICP-OES 13.3 ± 0.2^79^ 17.0 ± 0.3^79^ 3.65 ± 0.31^78^ 3.82 ± 0.2^21^ 3.16 ± 0.2^21^ 5.4 ± 0.8^103^ 5.2^103^ ASA: 6.50 ± 0.55^137^	6.37 (0.69)	ICP-OES 29.4 ± 1.1^79^ 48.7 ± 5.8^79^ 7.44 ± 1.41^78^ 8.84 ± 0.5^21^ 6.78 ± 0.8^21^ ICP-MS 25 ± 5^28^ 28^28^	2.35 (0.052)	ICP-OES 0.95 ± 0.18^78^	3.78 (0.61)	ICP-OES 1.951 ± 0.204^78^	6.61 (0.25)	ICP-OES 18.4 ± 0.5^79^ 21.6 ± 0.6^79^ 4.52 ± 0.35^78^ ASA: 9.04 ± 1.25^137^	1.90 (0.15)	ICP-OES 1.107 ± 0.069^78^ ASA: 2.34 ± 0.44^137^
**Zn**	125 (29)	ICP-OES: 103.0 ± 0.4^79^ 92.7 ± 0.7^79^ 24.39 ± 1.62^78^ 30.91 ± 1.7^21^ 29.43 ± 1.8^21^ 32.8 ± 3.6^103^ 32.3^103^ ASA: 25.3 ± 3.8^137^	32 (13)	ICP-OES 80.4 ± 1.2^79^ 74.0 ± 0.3^79^ 29 ± 2^28^ 32^28^ 18.79 ± 1.52^78^ 18.67 ± 1.1^21^ 17.96 ± 1.5^21^	58 (180)	ICP-OES 10.59 ± 2.93^78^	50 (110)	ICP-OES 10.77 ± 0.72^78^	25.4 (5.1)	ICP-OES 62.9 ± 0.3^79^ 68.7 ± 0.4^79^ 14.502 ± 0.75^78^ ASA: 17.9 ± 1.1^137^	159 (360)	ICP-OES 16.98 ± 1.23^78^ ASA: 20.4 ± 1.7^137^
**Se**	1.25 (0.11)	ASA: 0.720 ± 0.030^137^ 0.65 ± 0.06^138^ ICP-MS: 1.4 ± 0.2^103^ 1.4^103^	1.64 (0.15)	ICP-MS 1.6 ± 0.2^28^ 1.9^28^	0.120 (0.019)		0.160 (0.033)		0.364 (0.032)	ASA: 0.430 ± 0.110^137^	0.520 (0.019)	ASA: 0.500 ± 0.080^137^

Based on the data presented in the Fig. 7 and Table 5, the analyzed elements can be classified into three groups: macroelements (with concentration higher than 1000 ppm), microelements (with concentration higher than 100 ppm and lower than 1000 ppm) and trace elements (with concentration lower than 100 ppm). The elements that in all organs persisted at the level of macroelements were: P, S and K. In case of Ca, the concentration above 1000 ppm was observed in the liver, muscle and spleen. In the three remaining organs, Ca was qualified to the micronutrient group. Fe and Zn concentrations above 100 ppm were recorded for liver and spleen. In the remaining organs, their contents were determined as trace. Cu and Se in all the examined organs were classified into the group of trace elements. The TXRF results, presented in the Table 5, pointed also at high variability in the concentrations of the studied elements in tissues taken from individual animals. The high population variability concerned, mainly, the accumulation of Ca and Zn, and in some organs also Fe.

The aim of the study was also to compare the results of the elemental analysis of rat organs carried out using the TXRF with available literature evidence. The elemental data obtained using the techniques of AAS, ICP-OES and ICP-MS were used for this purpose. In the case of light elements, the concentrations determined using the TXRF were compared with the results obtained with ICP-OES technique by Leblondel et al.^78^ and Shimamura et al.^28,103^*.* It was noted that in all organs, the P, S, K and Ca concentrations obtained using the TXRF were higher than those determined with ICP-OES^28,78,103^ or consistent with them within the limits of observed population dispersion (concentration of S in kidney and heart^78^). The higher levels of light elements measured in our study may be influenced by the sample preparation method. Leblondel et al. used digestion of sample for 3 h at room temperature and then next 3 h at 70 °C in the presence of HNO_3_^78^. Shimamura et al. performed sample decomposition using HNO_3_ and H_2_O_2_ in high temperature (180 °C) conditions and they repeated that procedure 2 or 3 times^28,103^. No clear information on whether the digestion was performed in a close or open system was found in the mentioned papers. In our study, for tissue digestion we applied wet mineralization in a closed system using microwave energy. The technique is very often used to digest biomedical samples of different types, including the samples of organs^20,52,101,102^, body fluids^17,32,83^ or bones^12^*.* To its advantages one can include small amount of sample required for analyte preparation as well as the limitation of the risks of sample contamination and loss of light elements by limiting their volatility during digestion^10,38^.

Concentrations of Fe, Cu, Zn and Se measured with TXRF were also confronted with the literature values obtained with various analytical techniques. In contrary to macroelements, the values determined with TXRF and measured with other techniques were usually in quite good accordance. The highest agreement was observed for Ca, Zn and Se levels for all the organs, excluding liver.

### Comparison of validation parameters obtained for TXRF with other techniques of elemental analysis

Based on the obtained results, for each organ the detection limits of the elements were calculated and compared with the values of LODs obtained for other analytical techniques and published in the literature (Table 6). The detection limits of elements are expressed either in mass or volume units. For general comparisons between TXRF and the discussed techniques, only LODs values expressed in mass units are useful and therefore they are placed in the Table 6. Additionally, the values of LODs determined for real biological samples are underlined. The remaining, not underlined values were in most cases determined in blank measurements. Additionally, when the detection limits for the element was not found in the papers dealing with the analysis of biomedical samples, the investigations concerning environmental or food samples were used for comparisons.

**Table 6:** Comparison of LODs of elements in [μg/g] obtained for examined organs using TXRF with the literature values for other analytical techniques.

	LOD calculated for TXRF measurements						Literature values of LOD for other techniques			
	Liver	Brain	Heart	Kidney	Muscle	Spleen	F-AAS	GF-AAS	ICP-OES	ICP-MS
**P**	19.5 (4.9)^#^	13.3 (1.4)	11.7 (1.9)	14.1 (3.5)	28.6 (7.5)	19.8 (3.9)	n.f.	0.5 μg/g^a^^139^	0.68 μg/g^a^^140^	n.f.
**S**	5.0 (1.0)	4.96 (0.50)	4.80 (0.61)	5.3 (1.7)	10.2 (2.5)	6.79 (0.61)	30 μg/g^a^^141^	n.f.	8.7 μg/g^a^^142^	0.004 μg/g^a^^143^
**K**	2.37 (0.59)	2.16 (0.26)	1.76 (0.30)	1.88 (0.57)	4.5 (1.3)	3.06 (0.51)	0.01 μg/g^a^^144^ 0.1 μg/g^a^^144^	0.0032 μg/g^a^^145^	0.68 μg/g^a^^140^	3 μg/g^38^
**Ca**	1.49 (0.51)	0.74 (0.25)	0.66 (0.25)	0.72 (0.23)	1.9 (1.1)	1.44 (0.71)	0.01 μg/g^34^	n.f.	0.005 ^69^	0.801 μg/g^101^
**Fe**	0.24 (0.18)	0.116 (0.014)	0.111 (0.015)	0.129 (0.040)	0.266 (0.082)	0.229 (0.035)	0.08 μg/g^34^	1.6 μg/g^45^	0.5 μg/g^69^–2.3 μg/g^45^	0.00149 μg/g^98^–4 μg/g^38^
**Cu**	0.095 (0.021)	0.0764 (0.0084)	0.067 (0.011)	0.080 (0.025)	0.174 (0.058)	0.116 (0.024)	0.03 μg/g^34^	0.00000017 μg/g^60^–0.2 μg/g^45^	0.0109 μg/g^76^–2.1 μg/g^45^	0.00059 μg/g^98^–0.1 μg/g^38^
**Zn**	0.092 (0.021)	0.0722 (0.0080)	0.065 (0.014)	0.082 (0.027)	0.176 (0.054)	0.123 (0.026)	0.03 μg/g^34^	0.3 μg/g^45^	0.0357 μg/g^76^–1.1 μg/g^45^	0.00251 μg/g^98^–0.9 μg/g^101^
**Se**	0.0413 (0.0058)	0.0403 (0.0055)	0.0335 (0.0035)	0.043 (0.013)	0.090 (0.026)	0.0527 (0.0052)	0.0032 μg/g^ab^^146^	0.26–1.0^a^^147^	0.0002 μg/g^56^	0.0015 μg/g^101^–0.12 μg/g^18^

^#^LOD in [μg/g] together with the uncertainty calculated as standard deviation;

^a^Value from the paper that does not concern the analysis biomedical samples;

^b^Determined using HG-AAS;

*n.f.* value not found in the discussed papers or not expressed in consistent unit.

As one can notice from Table 6, the lowest limits of detection in our study were found for heart samples. They ranged from 0.0335 µg/g in case of Se to 11.7 µg/g for P. For most of the elements, the direct comparison between values of LODs obtained in the frame of our work and found in the literature is difficult, due to different sample matrices (real biological samples or blank samples) and/or inconsistent units. The exceptions are Ca and Fe, for which one can find the literature values of this parameter determined by Yang et al. for ICP-OES. The values of LODs were obtained there for the biological matrix, namely rabbit arteries sample, and were expressed in µg/g^69^. The Ca detection limits obtained for examined organs using TXRF are higher than determined for ICP-OES. In turn, those obtained for Fe are better than presented in the work of Yang et al.^69^*.*

The general insight into the data presented in the Table 6 allows to conclude that the LODs received using TXRF for P are worse comparing to GF-AAS and ICP-OES, for which this parameter was obtained based on blank measurements. In the case of S, values of LODs received in our work are better than those of F-AAS for all examined organs and of ICP-OES for most of them, but much worse than obtained for ICP-MS. The LODs of K are similar to the values found for the ICP-MS but worse than for other discussed techniques in case of which the parameter was calculated for blank measurements. The Ca LODs for the TXRF are worse comparing to F-AAS and ICP-OES but similar to those of ICP-MS. The LODs of Fe achieved using TXRF are better than those of GF-AAS and ICP-OES and are within the range of values obtained for ICP-MS. They are, however, worse than Fe LODs achieved using F-AAS. Similar conclusions can be made for Cu, Zn and Se. However, for the last element better detection limits were obtained using ICP-OES than for TXRF.

To assess the reliability of the used analytical technique, the quantitative analysis of IAEA-A-13 reference material was performed. The values of trueness and repeatability calculated for TXRF are placed in Tables 7 and 8, respectively. The Tables contain also the lowest and highest values of the parameters found in the examined literature. If information about repeatability or trueness of techniques was not found in the papers concerning the analysis of biomedical samples, the data from environmental or food samples were used.

**Table 7:** Values of trueness [%] obtained using TXRF for the reference material IAEA-A-13 together with the lowest and highest values of the parameter met in examined literature.

Element	Recommended value [μg/g]	95% Confidence Interval [μg/g]	Measured value [μg/g]	TXRF	F-AAS	GF-AAS	ICP-OES	ICP-MS
P	940^#^	690–1120	653	69	n.f.	99.7^139^*^a^	90^13^–99.8^84^*	104^119^
S	6500	6000–7000	6219	95	96.1–109.4^a^^141^	n.f.	99 ± 14^a^^148^, 91 ± 2^a^^148^	100^119^
K	2500	2100–2700	2877	115	94–101^a^^144^	88 ± 7–104 ± 8^a^^145^	72^13^–104^84^*	98^119^–98.7^38^*
Ca	286	226–332	469	164	102.4^34^	n.f.	94^71^*–105^69^	64^96^*–105^39^*
Fe	2400	2200–2500	3048	127	97.8^34^	97^45^*–106^105^*	91.5 ± 1.8^82^–113^47^	71^101^*–111^101^*
Cu	4.3	3.7–4.8	5.6	130	104.1^34^	96.4^60^*–103.8^110^*	90^47^–108^86^*	81^100^*–125^101^*
Zn	13	12–14	18	140	95.1^110^*–95.9^110^*	97^45^*	87^79^–113^71^*	111 ± 30^95^
Se	0.24	0.15–0.31	0.22	92	97.8 ± 5.2^a,b^^146^, 102.2 ± 5.3^a,b^^147^	89.4–98.8^a^^147^	91^83^–109^3^*	62^100^*–125^101^*

^#^Information value;

*Calculated based on the literature data;

^a^Value from the paper that does not concern the analysis biomedical samples;

^b^Determined using HG-AAS;

*n.f.* Value not found in discussed papers or not expressed in consistent unit.

**Table 8:** Values of repeatability [%] obtained using TXRF for the reference material IAEA-A-13 with the lowest and highest values of these parameters met in examined literature.

Element	TXRF	F-AAS	GF-AAS	ICP-OES	ICP-MS
P	11.7	n.f.	n.f.	1.0^13^–3.0^35^	0.9^119^–3.9^119^
S	5.1	n.f.	n.f.	5.7^a^^148^, 1.1^a^^148^	0.8^119^–3.6^119^
K	2.9	0.7–9.4^a^^149^	4–7^a^^145^	0.6^35^–4.7^13^	1.2^119^–3.1^119^
Ca	5.4	0.7–4.3^a^^150^	n.f.	2.0^35^–6^16^	1.2^90^–9.5^119^
Fe	5.1	1.2–10.0^a^^149^	< 7^a^^151^	0.5^83^–25^25^	1.13^96^–4.1^90^
Cu	5.7	3.4^a^^152^, 7.2^a^^152^	1.29^60^–7.35^60^	0.6^83^–11.1^26^	0.7^100^–9.7^89^
Zn	4.1	0.8–8.6^a^^149^	8–15^a^^153^	0.3^83^–15^25^	0.2^100^–22.8^95^
Se	28.0	1.4–11.7^ab^^146^	< 10^a^^147^	9.1^83^–9.3^83^	1.8^17^–30^35^

^a^Value from the paper that does not concern the analysis biomedical samples;

^b^Determined using HG-AAS;

*n.f.* Value not found in discussed papers or not expressed in consistent unit.

Based on the data presented in Table 7 it can be seen that for most of the elements, the values of trueness obtained using TXRF are a little worse than those received using other instrumental techniques. The exception is the values of trueness for S and Se which in case of the TXRF, were equalled to 95% and 92%, respectively. In turn, the greatest discrepancies were observed for Ca (164%) and Zn (140%).

As one can notice from Table 8, for most of the elements the repeatability of the technique was in the range 2.9–5.7%. The values of this parameter calculated for S, K, Ca, Fe, Cu and Se were in good agreement or were slightly higher than that met in literature for other techniques. Exceptions were the values obtained for P and Se, in case of which this parameter was equal to 11.7 and 28.0%, respectively. Such high spread of results for Se may be connected with its low concentration in analysed reference material.

## Conclusions

The comparison of the validation parameters of the discussed instrumental techniques, done based on the literature studies, pointed out that ICP-MS offers the best analytical possibilities. The technique allows multielemental analysis, including the determinations of ultra-trace elements due to the very low detection limits. Also precision and trueness of the results for ICP-MS are usually better than for other techniques of elemental analysis which makes it a powerful tool in case of biomedical samples examinations. Its widespread use in research is, however, limited, due to the high costs of both the instrument and the analysis of a sample. A very good analytical tool in term of the achieved detection limits of elements is GF-AAS and the detection limits of Be, Cr, Ni or As obtained using this technique are lower or comparable with the values for the plasma techniques.

The physiological concentrations of P, S, K, Ca, Fe, Cu, Zn and Se were determined using TXRF in the rat livers, kidneys, muscles, brains, hearts and spleens and compared with the literature data. Some discrepancies were observed for light elements (P, S, K, Ca) which may be connected with the used methods of tissue preparation. For higher-Z elements (Fe, Cu, Zn, Se) the differences were smaller and the results obtained using TXRF usually agreed with the data from other techniques within the limits of observed population dispersions.

Due to inconsistences in the type of matrices of examined samples and units in which detection limits were expressed, direct comparison of LODs values received for TXRF and the discussed techniques was difficult. The exceptions are detection limits of Ca and Fe achieved in ICP-OES for biomedical sample. LODs of Fe received in our work was better than found in the literature. In turn, the detection limits of Ca were higher for TXRF than for ICP-OES. The general comparison of discussed analytical techniques in respect of achieved LODs, showed that the values obtained using TXRF are usually better than those of GF-AAS for Fe, Zn and Se, than F-AAS for S and ICP-OES for Fe. The detection limits calculated in this work are within the range of the values determined for ICP-MS in case of Fe, Cu, Zn and Se, for ICP-OES in case of Cu and Zn, and for GF-AAS in case of Cu. TXRF, in terms of LODs, occurred to be worse than F-AAS for K, Ca, Fe, Cu, Zn and Se, than GF-AAS for P and K, than ICP-OES for P, K, Ca and Se and than ICP-MS for S.

Our results showed that the precision of the TXRF is comparable with those of other discussed instrumental techniques. The repeatability of the technique is good and for most of the examined elements is within the range of 2.9–5.7%. Some discrepancies between the reference and the measured values of concentrations for the IAEA-A-13 reference material were, however, observed.

## Supplementary Information

Supplementary Information
