# Supplementary material for: The assessment of the usability of selected instrumental techniques for the elemental analysis of biomedical samples
Source: Sci Rep. 2021 Feb 12;11:3704. doi: 10.1038/s41598-021-82179-3 (PMC7881205; doi:10.1038/s41598-021-82179-3)
Supplement: Supplementary file 1 — Supplementary Information [file 41598_2021_82179_MOESM1_ESM.docx]

**Supplementary materials**

Table S1 – Details concerning the type of sample and preparation methods in particular papers discussed in current work.

| Ref. | Type of sample | Preparation |
| --- | --- | --- |
| 3. | human brain, heart, muscle, kidney, liver, ovary, pancreas and spleen | Organs were pre-washed and surround surface tissues, fatty tissues, membranes and blood vessels were removed. Approximately 1 g of wet tissue was digested with 5 ml of HNO_3_ and 2 ml of H_2_O_2_ using microwave-assisted system. Then, sample solutions were evaporated to volume 0.3 - 0.9 ml and diluted up to 3 ml with deionized water. Yttrium was used as internal standard and added to sample prior to mineralization. The reference materials were prepared in the same way. |
| 5. | human blood and serum | Blood and serum samples were prepared by a 8-fold and 5-fold dilution with a Nash Reagent (containing nitric acid, ammonium hydroxide, Triton X-100, antifoam B and EDTA). Magnesium nitrate and palladium/citric acid were tested as chemical modifiers |
| 8. | human erythrocytes, blood, plasma, urine and liver | 1-2 ml of blood were mixed gently before preparation. 500 μl of blood sample was diluted with 500 μl solution of Triton X-100 (0.02%) and internal standard (25 μg/l Rh). Then, 150 μl of ammonia solution (20%, v:v) was added and finally sample was diluted to 5 ml. Plasma samples were prepared in the same way as blood. Erythrocytes, after isolation from blood, were washed three times with 1 ml of 0.9% NaCl (m:v). Then, solution with cells was filled up to 1 ml with Triton-X-100 (1%, v:v) and mixed. Prior to analysis, 500 μl of the sample solution and 500 μl of internal standard solution (25 μg/l Rh) were diluted to 5 ml with ammonia solution (0.5%). Urine samples were prepared in both ways. For total As and Cr determination urine was diluted 1:10 (v:v) in a solution of HNO3 (1%), internal standard (2.5 μg/l Rh) and ethanol (1%, v:v). For As species determination, 500 μl of urine was diluted to 4.5 ml with deionized water. Liver tissue samples were placed in tube and dissolved with 2 ml of 32% nitric acid (v:v) at 90 °C for 1 h. 500 μl of resulting solution was diluted with 500 μl of internal standard solution and filled up with deionized water to 4 ml. |
| 9. | human brain | Brains were fixed in formalin and stored at least 27 days. After that time, they were dissected into slices and tissue specimens (0.011-3.370 g wet mass) were taken. Then, tissue specimens were transferred into polypropylene container and freeze-dried. Amount of 0.1 mg of each sample was digested with nitric acid in microwave-heated autoclave. |
| 12. | human bone, serum and dialysis fluid | Bone samples were washed, defatted, freeze-dried and stored at -70 °C. About 0.2 - 0.5 g segments of bone were put in Teflon vessels to which concentrated nitric acid V (10 ml) was added and then closed microwave digestion were performed. Serum and dialysis fluid were diluted (1+2) with a solution containing Triton X-100 (0.15%, v:v) and Ca (as Ca(N0_3_)_2_, used as chemical modifier). |
| 13. | rat liver | Frozen liver tissues were cut into small pieces, freeze dried at -20 °C for 12 h and then dried in oven at 70 °C for 3h. 200 mg of the sample were pre-digested with mixture of HNO_3_ (68%, 7.5 ml) and HF (38%, 1.0 ml) in a Teflon beaker at 80 °C for 3h. Sample solutions were then transferred to Teflon vessels and heated in microwave digester. After cooling, the content of vessels were evaporated at 150 °C to dryness. Then HNO_3_ (1.0 ml) and HClO_4_ (0.5 ml, 70%) were added to samples and dried again. The obtained sediment was dissolved in 1ml of HNO_3_ (27%) and then diluted up to 20 ml. The reference samples were prepared in a similar way. |
| 14. | human blood | For GF-AAS: 200 μl of blood was mixed with 100 μl of Triton (10%), 200 μl of diammonium hydrogen phosphate (10%) and 200 μl of nitric acid (0.1 N). 10 μl of resulting solution was used for analysis.  For ICP-MS: 100 μl of blood sample was digested in 500 μl of 68% nitric acid using closed-vessel microwave digestion. The resulting solution was diluted to 5 ml with ultra-pure water. |
| 15. | human urine | 10 ml of urine was mixed with concentrated hydrogen peroxide (0.5 ml), concentrated nitric acid (100 μl) and Triton X-100 (100 μl of a 10%, w:v). |
| 16. | human cerebrospinal fluid | For ICP-AES: 100 μl of fluid were placed in tube and diluted with 80 mM HNO_3_ to 2.5 ml. Then, 0.5 ml of the acidified sample was transferred to tube and diluted to 5 ml with solution of Y (1 μg/ml in 80 mM HNO_3_).  For ICP-MS: 100 μl of fluid were placed in tube and diluted with 80 mM HNO3 to 2.5 ml. |
| 17. | human amniotic fluid | Each amniotic fluid sample (5 ml) was centrifuged for 10 min (at 3000 rpm, 4 °C), placed in Eppendorf tubes and frozen at -80 °C. Before analysis, amniotic fluids were defrosted and 1 ml of sample were microwave digested with HNO3 (0.5 ml, 65%) and H2O2 (0.5 ml, 30%). The resulting solutions were transferred to flasks and diluted to 10 ml with demineralized water. In the case of determination of Ca and Mg, samples were diluted 100-fold. The certified reference material was digested in a microwave digestion system and diluted with demineralized water to 10 ml. |
| 18. | human lung | The samples of tissues were dried in oven at 70 °C for three days until they reached constant weight. Then, each sample was divide into three small sub-samples (average weight was 10±0.6 mg) and individually transferred into microwave vessels, to which 2 ml of concentrated nitric acid and 1 ml of hydrogen peroxide were added. Then, vessels were sealed and microwave digested. The resulting solution was diluted to 10 ml with water. |
| 19. | human brain, stomach, liver, kidneys, heart, lungs and blood | Tissues were homogenized, while blood were mixed on a vortex. 1.5 ml of blood and 1.5 g of organ tissue were treated with mixture of nitric acid V and hydrogen peroxide (30%, v/v) in ratio 5:1 and digested for 24 h. The resulting solutions were diluted up to 10 ml with deionized water. The certified reference materials were treated in the same way. |
| 24. | human hair and fingernails | The samples were washed with acetone, chloroform and distilled water and then dried overnight at 60 °C. Dried samples were placed in beaker, to which 2 ml of concentrated HClO4 and 12 ml of concentrated HNO3 were added, and they were heated for 30 min at 150 °C. Next, about 5 ml of 30% hydrogen peroxide was added to samples and heated at 200 °C to dryness. To the residue, 10 ml of HNO3 (0.1 ml/l) was added and heated again for 10 min at 100 °C. The solution was diluted to final volume 200 ml, pH was adjusted to 5.5 and then solution was passed through the microcolumn, where metal ions where retained and then eluted with HCl solution. |
| 25. | human gallstones | Gallstones were crushed and homogenized and then 0.1 - 0.2 g of sample were dried in oven at 100±5 °C to constant weight. 0.1 - 0.2 g amount of dried sample was transferred into Pyrex vessels and digested using open-focused microwave digester. 5 ml of HNO_3_, 3 ml and 2 ml of H_2_O_2_ were added to the sample at next steps of digestion procedure which total time was 1 h 30 min. The resulting solution was filtered and diluted to 25 ml with double deionized water. |
| 26. | human teeth | After extraction, teeth were treated with Clorox solution to remove blood or germs and rinsed with triply distilled ultrapure water. Then, teeth were put for 5 min into 25 ml of 40% HNO3 and rinsed again with ultrapure water and acetone. Next, teeth were dried in oven for 5 min at 133 °C, weighted and transferred to test tube together with 25 ml of 10% HNO3. Test tubes were heated in water bath for 1 h at 80 °C and left overnight in room temperature. Then they were again heated to 80 °C for 1 h and cooled down. The resulting solutions were filtered and diluted to 25 ml with ultrapure water. |
| 34. | human scalp hair | The samples were washed with acetone and ethanol and rinsed with deionized water, then dried in oven (90 °C, 20 min). Amount of 1.0 g of dried hair samples were digested with nitric acid V (15 ml), then filtered and diluted to 25 ml with deionized water. |
| 35. | human bone-marrow fluid | Human bone-marrow fluid; the fluid samples were diluted with 1ml of 0.1 M Tris-HCl and centrifuged for 15 min at 3000 rpm to exclude bone pieces. Then, the 0.3 g of supernatant was digested with concentrated HNO3 (0.5 ml) at 100 °C. Samples were heated almost to dryness and then dissolved with 5 ml of mixture HNO3 (0.1 M) and internal standards. |
| 37. | human cerebrospinal fluid | After collection, samples of cerebrospinal fluid were stored in tubes at -20 °C. Before analysis, samples were diluted with deionized water as 1+4 (v+v). |
| 38. | human and animal thyroid tissue | Human dried thyroid tissue samples (10 - 30 mg) were digested in microwave oven with 0.5 ml of concentrated subboiled nitric acid and 0.2 ml of hydrogen peroxide (30%). Animal dried thyroid tissue samples (1 - 2 mg) were dissolved in 0.2 ml of ultrapure nitric acid (65%). The standard reference material (20 mg) were treated in the same way as human thyroid samples. |
| 39. | human liver | The freeze-dried human liver samples (0.5 – 2.0 mg) were placed into PFA vessels to which 100 μl of concentrated nitric acid was added. Then, vessels were closed, transferred in digestion vessels containing high-purity water (10ml) and microwave-assisted digestion was performed. The resulting solution was adjusted to 1 ml and before analysis diluted in 1:20. |
| 44. | human hair and urine | 10 ml of urine sample together with 10 ml mixture of concentrated 63% HNO3 and 70% HClO4 (2:1) were heated on a hot plate, Then, the acid was evaporated to dryness and to resulting residue 3 ml of HClO4 was added and heated again to dryness at 160 °C. Next, 5 ml of H2SO4 was added and heated for 1 min at 150 °C. The resulting solution was diluted to 50 ml. Human hair samples were cut into small pieces, washed (acetone and distilled water) and dried in oven at 100 °C. To 0.2 mg of dried hair sample, 12 ml of concentrated nitric acid and 2 ml of concentrated HClO2 were added. The solution was heated on a hot plate for 90 min and then cooled to 70 °C. 5 ml of 30% H2O2 was added to the sample solution, which was next heated at 200C to dryness. To the residue 10 ml of 1 M H2SO4 was added and heated at 100 °C for 1 h. Finally, it was dissolved in water and diluted to 50 ml. For sample preconcentration, cloud point extraction was performed. 10 ml aliquots of sample, Triton-X-114 (0.05%, w:v), dithizone (5x10-6 mol/l) and sulfuric acid were heated at 50 °C for 5 min. After phase separation, the supernatant phase was completely separated by a syringe. To the surfactant-rich phase 50 μl of THF (tetrahydrofuran) was added. 20 μl of final solution was mixed with 10 μl of chemical modifier (Pd(NO3)2, 0.1%, w:v). |
| 45. | human brain | Tissues were dried at 105 °C to constant weight and 0.1 g were used for further preparation. Then, for comparison, two different digestion methods were used: 1) thermal heating in stainless-steel acid digestion Parr bomb with 2.5 ml of 65% nitric acid (2 hours at 150 °C), 2) microwave-assisted digestion in a Teflon PFA vessels with 3.0 ml of 65% of nitric acid. The digested solutions were diluted to 50 ml with deionized water. |
| 46. | human blood, hair, stomach, liver, kidneys | For GF-AAS: The samples of organ tissues (10 g; previously homogenized in the case of liver and kidney), hair samples (0.25 g; washed and cut into small parts), blood and urine (5 ml) were digested twice in closed glass vessels in the mixture of nitric and sulfuric acids (2 ml and 10 ml, respectively).  For ICP-AES: Internal organs tissues (2 g), blood (2 ml) and urine (5 ml) were digested in microwave oven in high pressure Teflon vessels with mixture of nitric acid V (3 ml) and hydrogen peroxide (1 ml). |
| 47. | human hair and serum | Hair samples were washed, dried and chopped into small pieces. 5 mg of sample was placed into test tube together with 20 μl of concentrated HNO3. After 3 hours, to the tube with the sample 5.0 μl of PTFE emulsion (60%, m:v) and 2.0 μl of Triton X-100 (0.1%) were added. Solution was diluted with double distilled water to 50 μl. To 20 μl of serum 5.0 μl of PTFE emulsion (60%, m:v) and 2.0 μl of Triton X-100 (0.1%) were added, and then diluted to 50 μl with double distilled water. |
| 51. | human brain | The samples were chopped into small pieces during freeze drying. 20 mg of tissue were placed in a Teflon vessel in which 1.0 ml of HNO_3_ was added. Then heating of sealed vessels in microwave oven was performed until completely digestion of samples. The obtained solutions were cooled down and diluted to 5 ml. Potassium dichromate was used as chemical modifier. |
| 53. | human scalp hair | hair samples were washed with acetone and ultrapure water and dried in oven at 100 °C. Then, samples were pulverized (mean particle diameter of 0.8 μm was obtained). Hair powder (0.1 g) was suspended in ultrapure water and diluted to 25 ml. Palladium and magnesium nitrate were used as chemical modifiers and glycerol (0.4 % (m/v)) as wetting agent. |
| 55. | serum and fibrous breast capsule tissue | The serum and standard silicon solution were diluted with matrix modifier (200 μl + 800 μl, respectively). Tissues were dried at 90 °C to constant weight and then digested in nitric acid (about 1 g of tissue in 0.5ml of acid) at 90 °C for 1h. The resulting solutions were diluted with water (3.5 ml) and potassium hydroxide (50%, 1 ml). Lanthanum oxide and ammonium phosphate were used as chemical modifier and added during dilution of samples before analysis. |
| 56. | human breast cancer tissue | Different separated regions of breast tissue were freeze-dried at -64 °C for 2-30 hours. About 1 g of each sample was placed in digestion tube together with 5 ml of concentrated HNO_3_ for 3 hours at 100 °C on hot block digester. Then, 1 - 3 ml of H_2_O_2_ (30%) was added and heated again for 1 hour. The resulting solution was cooled down, filtered and diluted to 25 ml with deionized water. |
| 57. | human cancer breast tissue | The samples were placed in microwave vessels and dried at 37 °C to constant weight. 1 ml of HNO_3_ and 1 ml of H_2_O_2_ were added to the dried sample and digested in a microwave oven. Digestion solutions were cooled down and diluted to 5 ml with ultrapure water. |
| 59. | human blood, urine and scalp hair | Blood samples, after collection, were stored at -4 °C. Urine samples were filtered and acidified with concentrated HNO_3_ (65%) and stored at -4 °C. Hair samples were cut into pieces and washed with mixture of diethyl ether-acetone, non-ionic detergent solution and ultra-pure water. Then, they were dried for 6 h at 80 °C. Two methods of digestion were tested, conventional and microwave assisted. In the first case, 0.5 ml of blood and urine or 0.2 mg of human hair were placed into flasks, in which 5 ml of mixture HNO_3_ and H_2_O_2_ (2:1, v/v) was added. Then, samples were heated on a hot plate for 2 - 3 h at 80 °C. The resulting solutions were diluted up to 10 ml with 2 M HNO_3_ and stored in flasks. For microwave-assisted acid digestion, 200 mg of dried hair, 0.5 ml of blood and 1.0 ml of urine were placed into Teflon PFA vessels. Into each vessel 2 ml of HNO_3_ and 1 ml of H_2_O_2_ (30%) were added and left for 10 min. Then, vessels were sealed and heated in microwave digester. After cooling, the resulting solutions were evaporated almost to dryness and diluted with HNO_3_ to 10 ml. The certified reference materials were treated in the same way. |
| 60. | human blood, urine and scalp hair | Venous blood samples, after collection, were stored at -4 °C. Urine samples were acidified with concentrated HNO_3_ and stored at -4 °C. Prior to subsampling, the samples were shaken to ensure homogeneity. Hair samples were cut into pieces and washed. Duplicate samples of dried hair were placed in PTFE flasks, while 0.5 ml of blood and urine samples were transferred into Teflon PTFE flasks. To each flask, 2 ml of mixture of concentrated HNO_3_ and H_2_O_2_ (2:1, v/v) was added and stored for 10 min at room temperature. Then, flasks were placed in covered PTFE container and microwave digested (2 - 4 min for blood and urine and 5 - 8 min for hair). After cooling, the resulting solutions were evaporated to remove acid. Then, 10 ml of nitric acid (0.1 M) was added to the residue, mixed and filtered. The certified reference materials were treated in the same way. For Co and Mn determination Mg (NO3)2 was used as chemical modifier, while Mg (NO3)2 + Pd (NO3)2 was used for As. |
| 63. | human blood and serum | Whole blood and serum samples (0.5 ml) were placed in PTFE flask with 3 ml mixture of HNO_3_ and H_2_O_2_ (2:1, v:v) and left for 10 min at room temperature. Then, flasks were placed in PTFE container and heated for 3 - 4 min. The resulting sample solutions were diluted with HNO_3_ to 10 ml. Then, cloud-point extraction procedure was performed. Digested samples were placed in tubes where 0.2 - 1.0 ml of APDC (0.1 - 0.5%), 2 ml of buffers (0.1 mol/l HNO_3_ and NaOH) and 2 ml of Triton X-114 (0.1 - 1%, v/v) were added. Then, tubes were heated at 40 - 60 °C for 10 - 30 min in ultrasonic bath. Phase separation was accelerated by centrifuging at 3500 rpm for 5 min. After cooling the solutions, the upper aqueous phase was decanted and to surfactant-rich phase acidic ethyl alcohol (0.5 ml, 0.1 mol/L HNO_3_) was added. |
| 64. | human scalp hair | 200 mg of samples were treated with 2 ml mixture of HNO3 and H2O2 (2:1, v:v) and left for 10 min; then they were digested by heating in PTFE container. The obtained solution was evaporated to dryness and diluted with HNO3 to 10 ml. Dual-cloud point extraction was performed in two steps. First, liquid sample was transferred to centrifuge tubes, into which 0.5 ml of PAN, 2 ml of Triton X-112 (0.1 - 0.5 %, v:v) and 2 ml of phosphate buffer were added. The pH of the solution was adjusted to range 7 - 11. The tubes were heated at 30 - 60 °C for 2 - 20 min. After centrifugation (5 min at 3500 rpm) separation of the phases was obtained. The aqueous phase was removed and surfactant-rich phase was treated with 2 ml mixture of HCl and HNO3 (0.5 - 2.0 mol/l) and heated at 30 - 60 °C for 5 - 20 minutes. Then, tubes were centrifuged (5 min at 3500 rpm) and supernatant was analysed. Standard solutions and blanks were prepared in the same way. |
| 69. | rabbit arteries | The samples were washed and dried at 60 °C for 48 h to constant weight. Then, they were placed in PFA Teflon vessels together with 2 ml of nitric acid (50%, v:v) and digested using close-vessel microwave system. The resulting solutions were cooled to room temperature, transferred to sterile tubes and diluted with distilled and deionized water to 5 ml. Before analysis, sample solutions were mixed by hand-shaking. A standard reference material was prepared in the same way. |
| 71. | human brain | The samples of brain tissue were dried in oven at 105 °C for 36 h till they reach constant mass. Two types of digestion were tested: 1) 100 mg of sample and 2.5 ml of nitric acid (65%) were digested in a Parr high-pressure bomb at 150 °C for 2 h, 2) 100 mg of sample was heated in microwave digester with 3 ml of nitric acid (65%). After cooling, the obtained solutions were diluted to 5 ml with distilled water. |
| 76. | sheep liver, kidney and muscle | Tissues were thawed and homogenized. For each type of tissue, 1 g of sub-sample were digested using nitric acid. |
| 77. | wolves liver | To 0.3 - 0.5 g portions of wet liver tissue 9 ml of HNO_3_ (65%) and 1 ml of H_2_O_2_ (30%) were added. Then samples were digested using microwave-assisted system. After digestion, samples were cooled down to room temperature and diluted to 25 ml with a distilled water. |
| 78. | rat whole blood, plasma, brain, heart, skeletal muscle, liver, kidney, spleen, thymus and bone | Organs tissues were first digested at room temperature for 3 h and then at 70 °C for another 3 h in nitric acid (about 1 ml of acid per 300 mg of tissue). The obtained solutions were diluted 1:10 with deionized water (1:1000 for determination of Ca in digested bone). The standard solutions were prepared in the same way. Analysis of blood and plasma were performed immediately without digestion. |
| 79. | rat liver, kidney, heart, lung and serum | Organ tissues were washed, dried and stored at -80 °C for further lyophilisation. About 0.5 g of each tissue were placed into PTFE vials together with 3.5 ml HNO3 (65%, v:v), 3.5 ml of H2O and 1 ml of H2O2 (30%, v:v). Then, the flasks were closed and microwave-assisted digestion was perform. After mineralization, the obtained solutions were diluted to 20 ml with deionized water. Samples of serum and plasma (aliquots of 0.1 ml) were diluted 100-fold with Triton X-100 (0.01%, v:v) to final volume 10 ml. |
| 81. | human whole blood, serum, cerebrospinal fluid and urine | 1 ml of blood was digested in microwave oven together with 2 ml of HNO_3_. To 1ml of urine 0.25 ml of HNO_3_ was added and diluted with water. Serum and cerebrospinal fluid were diluted with high purity deionized water. |
| 82. | human serum | 2g of serum was microwave digested with mixture of HNO_3_ (65%, 6 ml, v:v) and H_2_O_2_ (30%, 1 ml, v:v). |
| 83. | human serum | To 3 ml of serum sample 2ml of nitric acid, 1 ml of hydrogen peroxide and yttrium (11.2 μmol/l) were added. Sample solutions were digested in microwave oven. Then, the resulting solutions were cooled down, placed in vacuum module and evaporated till 0.6 – 1.0 ml volume of sample was obtained. The content of vessels was diluted to 3 ml with deionized water. The control and reference materials were prepared in the same way. |
| 84. | human rib bone | Tamples were transferred into one-chamber autoclaves together with 1.5 ml of concentrated HNO3 (65%) and 0.3 ml of H2O2. Then, they were heated at 160 – 200 °C for 3 h. After cooling the autoclaves to room temperature, sample solutions were diluted to 20 ml with deionized water and placed in measuring bottles. The standard reference material was treated in the same way. |
| 86. | human hair | The samples were dissolved by using nitric acid and hydrogen peroxide at higher temperature condition. Different temperature and time of digestion as well as composition of acid mixture were tested and optimized. For 1 g of hair sample, best results of mineralization were obtained for temperature in the range of 120-200 °C (150 °C was chosen), minimum 30 min of digestion time and 12 ml of HNO3-H2O2 at mixture ratio 2:1. |
| 89. | human plasma | The plasma samples were stored at -80 °C and prior to analysis they were reconstituted at room temperature. Then, samples were shaken and centrifuged for 15 min at 2000 g. 0.1 ml of plasma supernatant was diluted to 2 ml with HNO3 (0.5 %, v:v) and internal standard solutions in 1:20. The certified reference material was prepared according to manufacturer instructions, further diluted with HNO3 (0.5%, v:v). |
| 90. | human serum | 200 μl of serum were mixed with 250 μl of HNO_3_, 250 μl of H_2_O_2_, 250 μl of deionized water and 25 μl of an internal standard solutions (100 μg/l Rh and 1000 μg/l Se). Blank samples and reference materials were prepared in the same way, but instead the sample volume, 200 μl of deionized water or 200 μl of reference serum were used. The solutions were heated and shaken at 65 °C for 30 min and at 95 °C for another 30 - 45 min. After cooling, solutions volume loss was refilled using 25% HNO_3_. The resulting solution was diluted with 1.5 ml of deionized water. In the case of I determination, 25 μl of serum was diluted with NaOH (0.1 M) containing Rh (at 1 μg/l) up to 1 ml. |
| 95. | human brain | The samples were freeze-dried at -80 °C for 5 days until they reached constant weight and then they were removed from surface tissues and meninges. 50 - 150 mg of dried brain tissue were placed in Teflon vessels together with 2 ml of HNO3 and microwave digested. The resulting solutions were cooled down, transferred to tubes and diluted to 10 ml with MilliQ water. All analysed samples were diluted 1+4. |
| 96. | human esophagus tissue | About 0.1 g of dried tissues were placed into Teflon PFA vessels to which 8 ml of concentrated nitric acid was added. Then, microwave-assisted digestion of samples was performed. The resulting solutions were diluted to a final volume 10 ml with ultrapure water. |
| 98. | human liver | The samples were dried for 72 h at 105 °C to constant weight. Next, they were transferred to experimental tubes to which 2 ml of nitric acid of HNO3 (65%) and 1 ml of HClO4 (60%) were added. Samples then were heated for 8 h at 120 °C. The resulting solutions were diluted to 10 ml with deionized water. |
| 99. | human heart | Tissues were removed from paraffin wax, first mechanically with spatulas and then by immersing in xylene. Next, samples were dried in oven for minimum 3 h at 90 °C to completely dryness. Samples weighted 0.05-0.2 g were placed in microwave digestion vials to which 1 ml of hydrochloric acid and 4 ml of nitric acid were added. Each vessel was capped and samples were digested in microwave digestion system. The resulting solution was diluted up to 25 ml with reagent grade water. The certified reference materials were prepared in the same way, but without xylene treatment and drying steps. |
| 100. | human hair | 25 mg of ground hair samples were placed in tubes together with 2 ml of HNO_3_ (20%) and sonicated at 2 min (50 W, 100% amplitude). Then, liquid sample was diluted with aqueous solution of Rh (10μg/L) to 10 ml. The resulting diluted slurries were centrifuged (for 2 min at 900 x g) and analysis of supernatant was directly performed. |
| 101. | liver, diaphragm, brain and spleen of beef calves | About 1 g of tissue sub-sample were digested using microwave-digestion system together with 5 ml of concentrated nitric acid and 3 ml of hydrogen peroxide (30%, w:v). The resulting solutions were diluted with ultrapure water up to 15 ml. |
| 102. | rat livers, kidneys and urines | Rat organs were cut into small pieces and homogenized. Samples, each weighted 0.5 g, were placed in microwave digestion tank to which 4 ml of HNO_3_ and 1 ml of H_2_O_2_ were added. Then, microwave assisted digestion was performed. The resulting solution was evaporated at 150 °C for 2.5 h and the residue was dissolved in 2 ml 8-HQ solution and diluted with ammonium buffer solution to 50 ml. Aliquot of 40 ml was transferred into centrifuged tube, in which Triton-X-100 was added. Then the tube was shaken on vortex for 20 s and heated at 90 °C for 90 min. After a cloudy state was formed, the upper aqueous phase was removed and the gel-like sediment was obtained. Before analysis, sediment was dissolved with 1 ml of HNO_3_ (1%). |
| 105. | human brain | Brains were washed and removed from meninges. From different brain regions tissue fragments were collected and stored at -4 °C. After defrosting, samples were washed and dried in oven at 110 °C until they reached constant weight. Dried samples (100 - 500 mg) were placed in vessels, to which 2.5 ml of concentrated HNO_3_ (≥ 65%, w:w) and 1.0 ml of H_2_O_2_ (≥30%, v:v) were added. Samples were digested using microwave oven and, after cooling, diluted up to 50 ml with ultrapure water. Until analysis, the resulting solutions were stored in tubes at 4 °C. The certified reference materials were prepared in the same way. Mg(NO_3_)_2_ was used as matrix modifier. |
| 108. | animal muscle and liver | 2 - 10 g of homogenized sample was dried overnight in oven at 120±20 °C and then at 450 °C. After cooling the sample, 1 ml of concentrated nitric acid was added and dried on a hot plate. Then, sample was again heat at 450 °C for 1 h. After cooled the sample to room temperature, the carbon-free ash was dissolved in hydrochloric acid, transferred into flasks and diluted with 0.2 % nitric acid. Magnesium nitrate (1%) was used as a matrix modifier. |
| 110. | human liver | 1 - 2 g of wet liver samples were placed in tube and dried at 60 °C for 90 h. Then, 5 ml of nitric acid were added and solution was left for 72 h at room temperature. The standard reference material (0.5 g) was treated in the same way. |
| 118. | human blood, serum and urine | 2 ml of blood, 0.5 ml of serum and 20 ml of urine were digested with 3 ml of concentrated nitric acid V and 1 ml of 30% hydrogen peroxide using microwave digestion system. Before measurement, samples were diluted with deionized water. The reference materials were treated in a similar way. |
| 119. | E. coli cells | The cells were incubated at 37 °C for 24 h, suspended with 10 ml of 0.9% NaCl (g:g) and centrifuged at 4 °C for 5 min at 4500 x g. These steps were performed two times. After centrifugation, 10 mg of cells were transferred into vessel to which 1.45 ml of HNO_3_, 0.5 ml of H_2_O_2_ and 50 mg of internal standard solution (Rh and Re, 20 μg/l) were added. Then, samples were microwave digested. After mineralization, the solution was cooled down and mixed with 0.5 ml of water. The standard reference material were digested using microwave digestion system. Then, internal standard solutions were added and sample was diluted with MilliQ water up to 20 g. |

Table S2 - Details concerning the instrument and operating parameters in particular papers discussed in current work.

| Ref. | Technique | Instrument details | Operating parameters |
| --- | --- | --- | --- |
| 3. | ICP-OES | Ash IRIS/AP (Thermo Jarell, MA, USA) ICP-AES spectrometer; charged injection device detector; axial viewing mode; the Micromist nebulizer (Glass Expansion Pty. Ltd., Australia); spray chamber: cyclone; | plasma frequency: 27.12 MHz; Rf power: 1150 W; gas flow rates: 0.56 L/min (torch), 0.5 L/min (auxiliary), 0.56 L/min (nebulizer); nebulizer uptake: 0.5 ml/min; sample volume: 1.5 ml for 2 repeats; flush time: 40 s; purge time: 90 s; signal integration time: 60 s (high wavelength), 10 s (low wavelength); |
| 5. | GF-AAS | Varian AA280Z Zeeman atomic absorption spectrometer with a Zeeman background correction; GTA 120 graphite tube atomizer; PSD 120 programmable sample dispenser; beryllium hollow cathode lamp (Varian, Part No. 5610100500); | analytical spectral lines: 309.3 nm (Al), 234.9 nm (Be), 228.9 nm (Cd), 357.9 nm (Cr), 253.7 nm (Hg), 279.5 nm (Mn), 232.0 nm (Ni), 283.3 nm (Pb), 276.8 nm (Tl); argon flow rate: 0.3 L/min; operating conditions: 1) drying: 5 s at 85 °C, 30 s at 95 °C, 20 s at 120 °C, 2) pre-pyrolysis 450 °C for 22 s, 3) pyrolysis: 17 s at 1000 °C (serum) and 800 °C (blood), 4) atomizing: 3 s at 2900 °C (serum) and 2900 °C (blood), 5) cleaning: 2900 °C for 2 s; three experimental setups were tested: without modifier and with magnesium nitrate or palladium/citric acid as modifiers; |
| 8. | ICP-MS | total As and Cd determination - Agilent 7700x (Agilent Technologies, Waldbronn, Germany) spectrometer; Cetac ASX-500 Series autosampler (Cetac Technologies, Omaha, USA) set up in a laminar flow box FB 24 (Spetec, Erding, Germany); Babington nebulizer; Scott spray chamber (Agilent Technologies); injector tube with an inner diameter 2.5 mm;  As species determination - Agilent 1200 Series HPLC (Agilent Technologies); isocratic pump; autosampler, vacuum degasser; anion exchange column and HPLC column (Hamilton, PRPX100, 250x4 mm, particle size 10 μm); | isotopes: 52-Cr, 75-As; total As and Cd determinations - collision cell gas: 4 ml/min of He; collision/reaction gas: 1.4 ml/min of He and 4 ml/min of H_2_; generator power: 1500 W; gas flow rates: 15 L/min (outer); 1 L/min (intermediate), 1.2 L/min (nebulizer);  As species determination - mobile phase (Solution of 20 mM NH4HCO3, 8mM CH3COONa, 2.4 mM of NaNO3 and 1% ethanol, adjusted to pH 8.9) flow rate: 1.5 ml/min; sample injection volume: 50 μl; |
| 9. | ICP-MS | Agilent 7500ce (Agilent Technologies) spectrometer; | m/z: 56-Fe, 43-Ca, 65-Cu, 66-Zn, 24-Mg, 55-Mn; collision gas flow rate: 5.3 ml/min, He; |
| 12. | GF-AAS | Z5100 atomic absorption  spectrophotometer (Perkin-Elmer, Norwalk, CT, USA);  HGA 600 furnace system; transverse Zeeman-effect  background correction system; AS-60 autosampler; pyrolytic graphite-coated graphite tubes with solid pyrolytic graphite L'vov platforms | operating conditions: 1) dry: 230 °C, ramp 2 s, hold 30 s, Ar flow rate 300 ml/min, 2) pyrolysis: 1400 °C, ramp 3 s, hold 20 s, Ar flow rate 300 ml/min, 3) atomization: 2400 °C, ramp 0 s, hold 4 s, Ar flow rate 0 ml/min, 4) clean: 2650 °C, ramp 1 s, hold 10 s, Ar flow rate 300 ml/min; injection temperature: 100 °C; injection volume: 10 μl; |
| 13. | ICP-MS  ICP-OES | HP4500 (Yokogawa-Hewlett Co. Ltd, Tokyo, Japan);  SPS7000A (Seiko Electric Co. Ltd, Tokyo, Japan) spectrometer; | Plasma frequency: 27.12 MHz; Rf power 1.2 kW; argon flow: 14 L/min; sampling distance: 4.8 mm. |
| 14. | GF-AAS  ICP-MS | Hitachi polarized Zeeman  atomic absorption spectrophotometer (Z-5710, Hitachi High-technologies Corp., Tokyo, Japan); autosampler;  Thermo Scientific ELEMENT2 High Performance High Resolution ICPMS (Thermo Fisher Scientific Inc, Bremen, Germany); | Analytical spectral line: 288.8 nm (Cd);  Rf power: 1.250 kW; nebulizer: micro-uptake concentric nebulizer; plasma gas: argon; gas flow rate: cool gas 16 L/min, auxiliary gas 0.87 L/min, sample gas 0.979 - 1.190 L/min, additional gas 0.005 - 0.120 L/min; diameter of sampling cone orifice: 1.0 mm; diameter of skimmer cone orifice: 0.8 mm; mass resolution: medium resolution R=4,000; Samples per peak: 20; integration window: 60%; fore vacuum: 2-3 E-4 mbar; high vacuum: 1-2 E-7 mbar; |
| 15. | GF-AAS | Perkin-Elmer Model 1100B atomic absorption spectrometer equipped with deuterium-arc background correction; HGA-400 graphite-furnace atomizer (Perkin-Elmer); pyrolytic graphite coated tubes (part number B013-5653) and pyrolytic graphite platforms (part number B012-1092) (Perkin-Elmer); ATI-Unicam 939QZ spectrometer (Unicam Atomic Absorption, Cambridge, UK); GF90 graphite furnace; FS90 Plus autosampler; Pyrolytic tubes (Unicam); Zeeman correction; | analytical spectral lines: 313.3 nm (Mo), 309.3 nm (Al), 357.9 nm (Cr); lamp current: 12 mA (Mo), 10 mA (Al), 15 mA (Cr); wall atomization for Mo and Cr; platform atomization for Al; bandwidth: 0.7 nm; inert gas flow rate: Ar, 300 ml/min; |
| 16. | ICP-MS  ICP-OES | NexION 300D ICP-MS (Perkin Elmer, USA); 1.1 version of the control software; nickel cones; cyclonic spray chamber; Meinhardt nebulizer (type-C); standard factory tubing;  Spectro Ciros CCD ICP-AES; modified Lichte nebulizer; cyclonic spray-chamber; | isotopes: 47-Ti, 51-V, 55-Mn, 61-Ni, 66-Zn, 75-As, 85-Rb, 88-Sr, 107-Ag, 118-Sn, 138-Ba, 208-Pb; 3 readings of 30 sweeps; integration time: 75 ms; sample aspiration rate: 0.3 ml/min; sample pre-flush: 60 s;  analytical spectral lines: Ca 396.847 nm, Cu 324.754 nm, Fe259.941 nm, Mg 279.553 nm, P 177.495 nm, S 180.731 nm, Si 251.612 nm, Sr 407.771 nm, Zn 213.856 nm, K 766.491 nm, Na 330.237, Y 371.030 nm; aspiration of samples and standards: 2 ml/min; argon flow rates: 0.9 ml/min (nebulization), 0.9 ml/ min (auxiliary), 14.0 ml/min (coolant); plasma power: 1400W at 27 MHz; sample aspiration prior to collection for 45 s; signals collection: 3 x 24 s; |
| 17. | ICP-MS | ICP-MS Elan DRC II (PerkinElmer SCIEX, Canada); cyclonic spray chamber; concentric glass nebulizer; quartz torch with an injector; quadrupole mass analyzer with gold coated rods; calibrated autolens; Pt sampler and skimmer cones; detector mode: dual (pulse counting and analog mode); | isotopes: 24-Mg, 27-Al, 44-Ca, 51-V, 52-Cr, 55-Mn, 59-Co, 60-Ni, 63-Cu, 66-Zn, 78-Se, 88-Sr, 91-AsO, 111-Cd, 121-Sb, 138-Ba, 208-Pb, 238-U; DRC reaction gases: NH_3_, O; gas flow rates: 0.89 – 0.91 L/min (nebulizer),1.2 L/min (auxiliary), 16 L/min (plasma); Rf power: 1050 - 1150 W; scan mode: peak hopping; Dwell time: 50 ms per mass; |
| 18. | ICP-MS | X Series 2 ICP–MS and ICAP Q ICP–MS (ThermoFisher Scientific, Bremen, Germany); | four measurement methods: 1) standard mode - Thermo X Series 2 with 1% v/v nitric acid solution; calibration range 0.01 - 10 μg/L, 2) collision cell mode - kinetic energy discrimination with a collision cell gas (7% hydrogen in helium at a flow of 3.5 ml/min) using Thermo X Series 2 with 1% v/v nitric acid solution; calibration range 0.001 - 100 μg/L, 3) standard mode - ICAP Q with 1% v/v nitric acid solution; calibration range 0.002 - 0.2 μg/L, 4) standard mode - ICAP Q with 1%v/v HCl solution; calibration range 0.01 - 2.5 μg/L; |
| 19. | GF-AAS | Solaar MQZe atomic absorption spectrometer with Zeeman background correction; heated graphite atomizer (GF95) and autosampler (FS95) (Thermo Electron, Waltham, MA); | analytical spectral line: 232.0 nm (Ni); slit width 0.2 nm; sample solution volume: 20 μl; heating program details: 1) drying - 100 °C, ramp 10 s, hold 30 s, Ar flow 200 ml/min, 2) pyrolysis - 1,000 °C (1,300 °C) , ramp 150 s, hold 20 s, Ar flow 200 ml/min, 3) atomization - 2,500 °C (2,400 °C) , ramp 0 s, hold 3 s, Ar flow 0 ml/min, 4) cleaning - 2,600 °C, ramp 0 s, hold 3 s, Ar flow 200 ml/min; |
| 24. | F-AAS | Analytik Jena model nova 300 (Jena, Germany) F-AAS spectrometer; acetylene-air burner; single-element hollow cathode lamp (Cd and Pb); | analytical spectral lines: 228.8 nm (Cd), 283.3 nm (Pb); spectral slit: 1.2 nm; |
| 25. | ICP-OES | Thermo Jarell Ash model 25 ICP-AES (Waltham, MA, USA); cross-flow nebulizer; 2 mm i. d. plasma torch; polychromator with 2400 grooves/mm; | Rf: 27.12 MHz; Rf power: 1150 W; analytical spectral lines: 228.6 nm (Co), 267.7 nm (Cr), 324.7 nm (Cu), 259.9 nm (Fe), 257.6 nm (Mn), 231.6 nm (Ni), 213.8 nm (Zn) |
| 26. | ICP-OES | Perkin-Elmer Plasma 40 ICP-OES spectrometer; | analytical spectral lines: 324.754 nm and 224.700 nm (Cu), 213.856 nm and 202.548 nm (Zn), 220.353 nm and 216.999 nm (Pb); measurement time of each sample: the order of 2 min. |
| 34. | F-AAS | AA-6300 F-AAS spectrophotometer (Shimadzu); pre-mixed burner air-acetylene flame; | analytical spectral lines: Fe 248.3 nm, Cd 228.8 nm, Ca 422.7 nm, Zn 213.9 nm, Cu 309.93 nm, Pb 283.3 nm, Mg 285.2 nm, Cr 357.9 nm; |
| 35. | ICP-OES  ICP-MS | Plasma AtomComp MkII (Jarrell-Ash, Franklin, MA, USA); Pashen-Runge type of polychromator with 39 channels; fassel type torch; single type spray chamber; cross-flow type nebulizer.  Model SPQ 8000A (Seiko Instruments, Chiba) with quadrupole mass spectrometer; fassel type torch; scott type spray chamber; concentric type nebulizer; skimmer cone: Cu; | plasma Rf frequency: 27.12 MHz; plasma incident Rf power: 1.0 kW; Ar gas flow rates: 20 l/min (outer), 1.0 l/min (intermediate), 0.5 l/min (carrier); sampling observation height: 18 mm above work coil; sampling uptake rate: 1.2 ml/min; analytical spectral lines: Na 589.0 nm, K 788.4 nm, Fe 259.9 nm, P 213.6 nm, Ca 317.9 nm, Mg 279.0 nm, Al 308.2 nm, Zn 213.8 nm.  plasma Rf frequency: 27.12 MHz; plasma incident Rf power: 1.0 kW; Ar gas flow rates: 16 l/min (outer), 1.0 l/min (intermediate), 0.95 l/min (carrier); sampling depth: 12 mm from work coil; sample uptake rate: 0.8 ml/min; sampling cone: Cu, 1.1 mm orifice diameter;, 0.35 orifice diameter; data accumulation: 20 times; dwell time: 10 ms; repetition: 5 times; channel width: 3 channels; m/z: 85-Rb, 65-Cu, 82-Se, 138-Ba, 88-Sr, 91-Zr, 133-Cs, 121-Sb, 120-Sn, 98-Mo, 107-Ag, 184-W; |
| 37. | ICP-OES  ICP-MS  ICP-MS | Optima 3100 XL spectrometer (Perkin Elmer, Norwalk, CT, USA); cross-flow nebulizer with Ryton Scott chamber; polychromator equipped with an echelle grating; detector: simultaneous solid-state Segmented-array Charged-coupled device;  USA)  SF-ICP-MS ELEMENT model (Thermo Finnigan, Bremen, Germany); Guard Electrode device; nebulizer: Meinhardt glass type; water-cooled Scott type spray chamber; interface: Pt cones;  SF-ICP-MS (ELEMENT 2, Thermo Finnigan, Bremen, Germany); torch guard electrode device; Meinhardt-type glass nebulizer; water-cooled Scott chamber; | Rf power: 1.3 kW; Ar gas flow rates: 13.0 L/min (plasma), 0.5 L/min (auxiliary), 0.7 L/min (nebulizer); max. resolution: 0.006 nm at 200 nm; analytical spectral lines: 393.3 nm (Ca), 324.7 nm (Cu), 259.9 nm (Fe), 279.5 nm (Mg), 251.6 nm (Si), 213.8 nm (Zn);  Rf power: 1200 W; gas flow rates: 14 L/min (plasma), 0.9 L/min (auxiliary), 0.85 L/min (nebulizer); low resolution (LR): 300 m/Δm; medium resolution (MR): 3000 m/Δm; isotopes: 27-Al (MR), 9-Be (LR), 114-Cd (LR and MR), 59-Co (MR), 52-Cr (MR), 202-Hg (LR), 55-Mn (MR), 60-Ni (MR), 208-Pb (LR), 51-V (MR); mass window: 150% (LR), 100% (MR); search window: 100% (LR), 80% (MR); integration window: 80% (LR), 60% (MR); scans (number): electric (25);  isotopes: 138-Ba, 9-Bi, 7-Li, 100-Mo, 123-Sb, 120-Sn, 88-Sr, 205-Tl, 184-W, 90-Zr; resolution: low, 300 m/Δm; |
| 38. | ICP-MS | ELAN 6000 quadrupole-based mass spectrometer (Perkin Elmer Sciex, Ontario, Canada); microconcentric MicroMist nebulizer (Model MicroMist AR30-1F02); minicyclonic spray chamber (both from Glass Expansion, Pty. Ltd., Camberwell, Victoria, Australia); ELAN 6000 mass flow controller (MKS Instruments); peristaltic pump (Perimax 12, Spetec GmbH, Erding, Germany); | Rf power: 1350 W; cylindrical lens potential: 10.2 V; dwell time: 500 ms; mass range: 23 u - 238 u; scanning mode: peak hopping; optimization: max. ^137^Ba^+^ intensity; detection system dead time: 53 ns; gas flow rates: 13.5 l/min (coolant), 0.7 l/min (auxiliary); mass resolution: 300 m/Δm; solution uptake rates: 160 μl/min and 320 μl/min; nebulizer gas flow rate: 0.81 l/min and 0.76 l/min; no. of replicates: 8 and 10; measurement time: 3.2 min. and 4.0 min; |
| 39. | ICP-MS  TXRF | Thermo Elemental X7 ICP-MS spectrometer; normal flow, concentric, pneumatic nebulizer;  Atomika Extra IIA TXRF spectrometer; line-focused X-ray tubes; energy dispersive Si(Li) detector; | collision cell gas flow rate: 7.2 ml/min, 93% He - 7% H_2_; Rf power: 1410 W; Ar gas flow rates: 13 L/min (plasma), 0.95 L/min (auxiliary), 0.84 L/min (nebulizer); sample uptake rate: 0.82 ml/min; isotopes: 52-Cr, 55-Mn, 56-Fe, 60-Ni, 65-Cu, 66-Zn, 85-Rb, 208-Pb;  Excitation: Mo Kα 17.4 keV; data acquisition live time: 1000 s; K lines were used for determination of: Cr, Mn, Fe, Ni, Cu, Zn and Rb; L line was used for determination of Pb.; |
| 44. | GF-AAS | AA 6800G atomic absorption spectrometer (Shimadzu); graphite furnace atomizer (GFA-6500); autosampler (ASC-6100); bismuth hollow cathode lamp (Hamamatsu Photonics, L233 Series); Pyrolytic graphite-coated graphite tubes (P/N 206-69984-02) (Shimadzu); | analytical spectral line: 223.1 nm; spectral bandpass: 0.5 nm; hollow cathode lamp current: 10 mA; sample injection volume: 20 μl. |
| 45. | ICP-OES  GF-AAS | Labtest Plasmalab ICP-AES spectrometer (40 analytical channels plus monochromator); GMK-nebulizer with a Gilson Minipuls 2 pump; sample-loop injector.  Model SIMAA 6000 multielement GF-AAS system (solid state detection, Echelle polychromator optics, transverse-heated graphite atomiser with longitudinal Zeeman-effect background  correction; stabilised temperature platform furnace; true temperature control; AS-72 autosampler (Perkin-Elmer); | Rf: 27.12 MHz; Rf power: 2 kW;  inert gas flow rate: Ar 250 ml/min; integration time: 5 s; experimental conditions on each step: 1) 110 °C, 1 ramp time/s, 20 hold time/s, internal flow 250 ml/min, 2) 130 °C, 5 ramp time/s, 45 hold time/s, internal flow 250 ml/min, 3) 600 °C, 10 ramp time/s, 20 hold time/s, internal flow 250 ml/min, 4) 1600 °C, 0 ramp time/s, 5 hold time/s, internal flow 0 ml/min, 5) 2300 °C, 1 ramp time/s, 3 hold time/s, internal flow 250 ml/min. |
| 46. | ICP-OES  CV-AAS | iCAP 6300 emission spectrometer (Thermo Electron Corp., Waltham, MA, US); Echelle type monochromator; radio frequency - 27.12 MHz; radial/axial plasma observation;  Solar MQZe atomic absorption spectrometer with vapor system VP100 Continuous Flow Vapor Accessory; mercury absorption cell (Thermo Electron Corp., Waltham, MA, US); | range of recorded emission spectrum: 166.250 nm - 847.000 nm; |
| 47. | ICP-OES | ICP spectrometry source (2 kW, 27±3 MHz) (Beijing  Second Broadcast Equipment  Factory, China); conventional plasma torch; modified graphite furnace vaporizer; WDG-500-1A monochromator (Beijing Second Optics, Beijing, China); R456 type photo-multiplier tube (Hamamatsu, Japan); home-built direct current amplifier; U-135 recorder (Shimadzu, Japan); | incidient power: 1.1 kW; Ar gas flow rate: 0.5 L/min (carrier), 18 L/min (coolant); observation height: 12 mm; entrance slit width 25 μm; exit slit width: 25 μm; drying temperature: 100 °C, ramp 10 s, hold 20 s; ashing temperature: 500 °C, ramp 10 s, hold 50 s; vaporization temperature: 2400 °C; clear-out temperature: 2700 °C; vaporization time: 4 s; sample volume: 10 μl; |
| 51. | GF-AAS | Varian atomic absorption spectrometer (Model 475,  Sunnyvale, CA); graphite furnace atomizer (Model GTA-95); | analytical spectral line: 309.3 nm; bandpass: 0.5 nm; inert gas: Ar; volume of deposited sample: 20 mm^3^; operating conditions: 1) drying - 120 °C, 20 ramp time/s, 25 hold time/s, 2) ashing - 1400 °C, 10 ramp time/s, 40 hold time/s, 3) atomizing - 2600 °C, 1 ramp time/s, 2 hold time/s, 4) cleaning - 2600 °C, 1 hold time/s; |
| 53. | GF-AAS | Perkin-Elmer 1100B atomic absorption spectrometer; Perkin-Elmer HGA 700  graphite furnace; Perkin-Elmer AS 70 autosampler; Perkin-Elmer HGA 400 graphite furnace; Perkin-Elmer AS 40 autosampler; deuterium lamp as a background correction system; Pyrolytic graphite tubes; L’vov platform; | analytical spectral lines: 309.3 nm (Al), 279.5 nm (Mn); spectral bandwidth: 0.7 nm (Al), 0.2 nm (Mn); integration time: 3 s; peak-area measurements; D_2_ lamp background corrector; injection volume: 20 ml; operating conditions: 1) drying - 150 °C, ramp time - 20 s; hold time - 15 s, Ar flow - 300 ml/min, 2) pyrolysis - 1500 °C (Al) and 1200 °C (Mn), ramp time - 10 s; hold time - 15 s, Ar flow - 300 ml/min, 3) atomization - 2500 °C (Al) and 2200 °C (Mn), ramp time - 0 s; hold time - 3 s, Ar flow - 200 ml/min (Al), 4) cleaning - 2600 °C, ramp time - 2 s; hold time - 2 s, Ar flow - 300 ml/min; |
| 55. | GF-AAS | Varian Zeeman SpectrAA-300  AAS instrument (Varian Canada Inc.); pyrolytic coated graphite partition tube; | analytical spectral line: 251.6 nm; lamp current: 10 mA; sample volume: 10 μl; operating conditions: 1) 90 °C, furnace time 15 s, gas flow 3.0 L/min, 2) 95 °C, furnace time 10 s, gas flow 3.0 L/min, 3) 100 °C, furnace time 5 s, gas flow 3.0 L/min, 4) 300 °C, furnace time 5 s, gas flow 3.0 L/min, 5) 1400 °C, furnace time 19 s, gas flow 3.0 L/min, 6) 40 °C, furnace time 6.8 s, gas flow 0.2 L/min, 7) 2700 °C, furnace time 1.4 s, gas flow 0 L/min, 8) 2700 °C, furnace time 2.0 s, gas flow 0 L/min, 9) 2700 °C, furnace time 2.0 s, gas flow 3.0 L/min; |
| 56. | GF-AAS  ICP-OES | graphite furnace technique model AA-670;  ICP-OES spectrometer (ULTIMA 2CE); |  |
| 57. | GF-AAS | AAnalyst600 atomic absorption spectrometer, transversely heated graphite atomizer (THGA), longitudinal Zeeman-effect background corrector, AS-800 autosampler, WinLab 32 software (Perkin Elmer, UK); standard THGA graphite tubes, integrated L’vov platform (Perkin Elmer, UK); | operating conditions: 1) drying - 110 °C, ramp time 10 s, hold time 40 s, internal gas flow rate 250 ml/min, 2) drying - 130 °C, ramp time 15 s, hold time 40 s, internal gas flow rate 250 ml/min, 3) pyrolysis - 500 °C, ramp time 10 s, hold time 10 s, internal gas flow rate 250 ml/min, 4) pyrolysis - 1200 °C, ramp time 10 s, hold time 15 s, internal gas flow rate 250 ml/min, 5) atomisation - 2300 °C, ramp time 0 s, hold time 5 s, internal gas flow rate 0 ml/min, 6) clean out - 2500 °C, ramp time 1 s, hold time 5 s, internal gas flow rate 250 ml/min; |
| 59. | GF-AAS | Analyst 700 Perkin-Elmer atomic absorption spectrometer in conjunction with a graphite furnace model GF 3000 included PAL 3000 autosampler; | analytical spectral lines and slit-width: 283.3 nm and 1.3 nm (Pb), 228.8 nm and 1.3 nm (Cd), 357.9 nm and 1.3 nm (Cr), 232.0 nm and 0.2 nm (Ni); lamp current: 7.5 mA (Pb), 7.5 mA (Cd), 7.5 mA (Cr), 10 mA (Ni); background correction: D_2_ lamp; cuvette: cup (Pb and Cd) and tube (Cr and Ni); carrier gas flow rate: 200 ml/min; sample volume: 10 μl; temperature conditions - dry: 80 °C - 120 °C /15 s (Pb, Cd, Cr, Ni); ash: 300 °C - 600 °C /15 s (Pb, Cd) and 300 °C - 700 °C /15 s (Cr, Ni); atomisation: 2000 °C - 2100 °C /5 s (Pb), 1500 °C - 1800 °C /5 s (Cd), 2600 °C - 2700 °C /5 s (Cr), 2500 °C - 2600 °C /5 s (Ni); cleaning: 2100 °C - 2400 °C /2 s (Pb), 1800 °C - 2000 °C /2 s (Cd), 2700 °C - 2900 °C /2 s (Cr), 2600 °C - 2800 °C /2 s (Ni); |
| 60. | GF-AAS | Hitachi model 180-50 (Hitachi, Tokyo, Japan); Hitachi Model 056 recorder; hollow cathode lamps (Hitachi); | analytical spectral lines and slit-width: 193.8 nm and 2.6 nm (As), 240.7 nm and 0.2 nm (Co), 324.8 nm and 1.3 nm (Cu), 279.5 nm and 0.4 nm (Mn); lamp current: 10.0 mA (As), 10.0 mA (Co), 7.5 mA (Cu), 7.5 mA (Mn); background correction: D_2_ lamp; cuvette: cup; carrier gas (Ar) flow rate: 200 ml/min; sample volume: 10 μl; temperature conditions - dry: 80 °C - 120 °C /10 s (As) and 80 °C - 120 °C /15 s (Co, Cu, Mn); ash: 300 °C - 400 °C /10 s (As), 400 °C - 600 °C /15 s (Co, Cu) and 400 °C - 500 °C /15 s (Mn); atomisation: 2700 °C - 2800 °C /5 s (As), 2600 °C - 2700 °C /5 s (Co, Cu), 2500 °C - 2600 °C /5 s (Mn); cleaning: 2800 °C - 2900 °C /3 s (As), 2700 °C - 2800 °C /2 s (Co, Cu), 2600 °C - 2800 °C /2 s (Mn) |
| 63. | F-AAS | Perkin Elmer model AAnalyst 700 (Norwalk, CT) flame atomic absorption spectrophotometer; hollow cathode lamp of Ni; | analytical spectral line: 232.0 nm; spectral bandwidth: 0.7 nm; lamp current: 30 mA; |
| 64. | F-AAS | Perkin-Elmer Model AAnalyst 700 (Norwalk, CT, USA) flame atomic absorption  spectrophotometer; hollow cathode lamp of Mn; | analytical spectral line: 279.5 nm; spectral bandwidth: 0.2 nm; lamp current: 2.0 mA; acetylene flow rate 1.6 L/min; air flow 8.0 L/min; burner height 7.0 mm; |
| 69. | ICP-OES | Optima 3100-XL ICP-AES spectrometer (Perkin-Elmer, Norwalk, CT); concentric nebulizer; cyclonic spray chamber; axially-viewed system; segmented-array charge-coupled device; AS-91 auto-sampler; | analytical spectral lines: 317.99 nm (Ca), 238.20 nm (Fe), 279.08 nm (Mg); Rf power: 1.2 kW; gas flow rates: 15 l/min (outer Ar), 0.5 l/min (auxiliary Ar), 0.8 l/min (nebulizer); quantitative mode: peak area; background correction mode: two-point; |
| 71. | ICP-OES | Labtest Plasmalab ICP spectrometer; pneumatically operated injection valve; | argon plasma; incident power: 1.3 kW; Rf: 27.12 MHz; observation height: 16 mm; volume of injected sample: 130 μl; |
| 76. | ICP-MS  ICP-OES | ICP-MS spectrometer Elan 9000 (PerkinElmer, Waltham, MA); nebulizer: cross flow type; spray chamber: double pass;  Varian MPX ICP-OES spectrometer; axial view mode; nebulizer: concentric - sea spray; spray chamber: cyclonic baffled; | isotopes: 75-As, 111-Cd, 59-Co, 202-Hg, 98-Mo, 208-Pb, 82-Se; plasma Rf power: 1200W; gas flow rates: 15 l/min (plasma), 1.2 l/min (auxiliary), 1.0 l/min (nebulizer); auto lens: on; Dwell time: 200 ms; sweeps: 10; 3 replicates; read delay: 25 s; rinse delay: 25 s; pump rate: 13 rpm;  analytical spectral lines: 324.754 nm (Cu), 213.857 nm (Zn); plasma Rf power: 1200 W; gas flow rates: 15 l/min (plasma), 1.5 l/min (auxiliary), 1.0 l/min (nebulizer); replicate read time: 30 s; fitted background correction; |
| 77. | ICP-OES | ICP-OES spectrometer Spectro Genesis EOP II, Spectro Analytical Instruments (DmbH, Kleve, Germany); | analytical spectral lines: 394.401 nm (Al), 249.773 nm (B), 233.527 nm (Ba), 228.802 nm (Cd), 228.616 nm (Co), 205.552 nm (Cr), 324.754 nm (Cu), 259.941 nm (Fe), 184.950 nm (Hg), 460.289 nm (Li), 259.373 nm (Mn), 202.095 nm (Mo), 231.604 nm (Ni), 220.353 nm (Pb), 460.733 nm (Sr), 206.1919 nm (Zn); |
| 78. | ICP-OES | Jobin-Yvon JY 48 instrument composed of a vacuum  polychromator and a Plasma-Therm source; pneumatic nebulization; | analytical spectral lines: 455.40 nm (Ba), 393.37 nm (Ca), 324.75 nm (Cu), 259.94 nm (Fe), 766.49 nm (K), 279.55 nm (Mg), 403.08 nm (Mn), 386.41 nm (Mo), 589.0 nm (Na), 178.28 nm (P), 182.03 nm (S), 407.77 nm (Sr), 213.86 nm (Zn); |
| 79. | ICP-OES | Vista simultaneous ICP-OES  spectrometer (Varian - Mulgrave, Australia); axial viewing; charge coupled device solid state detector; V-Groove nebulizer; Sturman – master nebulization chamber; | analytical spectral lines: 226.502 nm (Cd); 238.203 nm (Fe); 327.398 nm (Cu); 280.267 nm (Mg); 220.354 nm (Pb); 196.026 nm (Se); 213.858 nm (Zn); Rf generator power: 1.3 kW; gas flow rates: 0.7 ml/min (nebulizer), 1.5 ml/min (auxiliary), 15 ml/min (plasma); signal integration time: 1.0 s; stabilization time: 15 min; reading time: 1 min; 3 replicates; |
| 81. | ICP-OES  ICP-MS | Optima 3100 XL spectrometer (Perkin Elmer, Norwalk, CT,  USA); cross-flow nebulizer; Ryton Scott chamber; polychromator with echelle grating; detector: simultaneous solid-state segmented-array charged-coupled device;  SF-ICP-MS spectrometer ELEMENT model from Thermo Finnigan (Bremen, Germany); torch guard electrode device; platinum interface cones; Meinhardt type glass nebulizer;  water-cooled Scott chamber; | analytical spectral lines: 393.3 nm (Ca); 324.7 nm (Cu); 259.9 nm (Fe); 279.5 nm (Mg); 251.6 nm (Si); 371.0 nm (Y), 213.8 nm (Zn); radiofrequency: 1.3 kW; argon gas flow rates: 13.0 l/min (plasma), 0.5 l/min (auxiliary), 0.7 l/min (nebulizer); max. resolution: 0.006 nm at 200 nm;  isotopes: 27-Al, 55-Mn; medium resolution mode: 3000m/Δm; radiofrequency power: 1.2 kW; gas flow rates: 14.0 l/min (plasma), 0.9 l/min (auxiliary), 0.85 l/min (nebulizer); |
| 82. | ICP-OES | ULTIMA 2 ICP-OES spectrometer (Jobin Yvon, Longjumeau Cedex, France); radial viewing mode; Czerny-Turner monochromator; Meinhard nebulizer; cyclonic spray chamber; | analytical spectral lines: 167.02 nm (Al), 238.204 nm (Fe), 257.610 nm (Mn), 213.856 nm (Zn), 588.95 nm (Na), 670.784 nm (Li), 766.490 nm (K); argon flow rate: 12 l/min; optical bench temperature: 32 °C; |
| 83. | ICP-OES | IRIS/AP ICP-AES spectrometer (Thermo Jarell-Ash, Franklin, MA, USA); charged injection device detector; axial viewing mode; microconcentric (Micromist) nebulizer (Glass Expansion Pty. Ltd., Australia); cyclone spray chamber; | analytical spectral lines: 396.152 nm (Al), 249.678 nm (B), 493.409 nm (Ba), 313.107 nm (Be), 214.438 nm (Cd), 237.862 nm (Co), 267.716 nm (Cr), 324.754 nm (Cu), 259.940 nm (Fe), 670.784 nm (Li), 257.610 nm (Mn), 231.604 nm (Ni), 220.353 nm (Pb), 196.090 nm (Se), 407.771 nm (Sr), 206.200 nm (Zn); plasma frequency: 27.12 MHz; Rf power: 1150 W; gas flow rates: 0.56 L/min (torch), 0.5 L/min (auxiliary), 0.56 L/min (nebulizer); nebulizer uptake: 0.5 ml/min; sample volume: 1.5 ml/2 repeats; flush time: 40 s; purge time: 90 s; signal integration time: 60 s (high wavelength) and 10 s (low wavelength); |
| 84. | ICP-OES | ICP-OES spectrometer ICAP-61 (Thermo Jarrell Ash, USA); angular nebulizer; | analytical spectral lines: 308.215 nm (Al), 249.678 nm (B), 493.409 nm (Ba), 393.366 nm (Ca), 324.754 nm (Cu), 259.94 nm (Fe), 766.491 nm (K), 670.784 nm (Li), 279.553 nm (Mg), 257.61 nm (Mn), 588.995 nm (Na), 213.618 nm (P), 182.04 nm (S), 421.552 nm (Sr), 292.402 nm (V), 213.856 nm (Zn); generator output power: 1.200 W; reflected power: <5 W, gas flow rates 18 L/min (plasma), 0.9 L/min (auxiliary), 0.6 L/min (nebulizer), sample  flow rate: 1.5 mL/min; zone height for plasma observation: 14 mm; integration time: 5 s; |
| 86. | ICP-OES | JOBIN-YVON  PANORAMA ICP-AES spectrometer; axial  viewing configuration; | analytical spectral lines: 259.940 nm (Fe), 324.754 nm (Cu), 237.862 nm (Co), 213.856 nm (Zn), 232.003 nm (Ni), 228.802 nm (Cd), 257.610 nm (Mn), 220.353 nm (Pb); Rf Power: 1.0 kW; nebulizer pressure: 3.0 bar; gas flow rates: 16 l/min (plasma), 1.7 l/min (auxiliary); sample uptake rate: 0.8 ml/min; stabilization time: 15 s; read time: 1 s; |
| 89. | ICP-MS | HR-ICP-MS Thermo Finnigan Element II model (Bremen,  Germany); concentric glass  nebulizer; water-cooled Scott double-pass spray chamber; torch with guard electrode device and nickel interface  cones; | isotopes: 11-B, 85-Rb, 88-Sr, 95-Mo, 111-Cd, 208-Pb (analysed in low resolution, 300 m/Δm), 27-Al,  51-V, 52-Cr, 55-Mn, 56-Fe, 59-Co, 63-Cu, 66-Zn (analysed in medium resolution, 4000 m/Δm), 75-As and  77-Se (analysed in high resolution, 10000 m/Δm); Rf power: 1280 W - 1300 W; argon gas flow rates: 16.0 L/min (cool), 0.94 L/min (auxiliary), 1.110 -1.200 L/min (sample); sample uptake rate: 0.1-0.2 mL/min; |
| 90. | ICP-MS | ICP-MS/MS system Agilent ICP-QQQ-MS 8800 (Waldbronn, Germany); MicroMist nebulizer; Scott type spray chamber; | two modes: 1) on-mass mode: He as collision gas, 2) mass-shift mode: O_2_ and H_2_ as reaction/collision gas mixture; parameters for mode 1) forward power: 1550, gas flow rates: 15 L/min (cool), 0.9 L/min (auxiliary), 1 L/min (nebulizer), 3 ml/min (cell - He), m/z: 24-Mg, 44-Ca, 55-Mn, 56-Fe, 65-Cu, 66-Zn, 103-Rh, 111-Cd, 127-I; integration time: 0.3 s; replicates: 3 s; parameters for mode 2) cell gas flow rate: 0.4 mL/min (O_2_), 1 mL/min (H_2_); m/z: 75-As, 77-Se, 78-Se, 80-Se, 98-Mo, 103-Rh; 91AsO, 93-SeO, 94-SeO, 96-SeO, 114-MoO; |
| 95. | ICP-MS | Elan 6000 ICP-MS spectrometer (Perkin Elmer, Sciex, Toronto, Canada); cross flow nebulizer; peristaltic pump; AS-91 autosampler fitted with a 152 position tray | isotopes: 111-Cd, 67-Zn; |
| 96. | ICP-MS | Agilent 7500 ICP-MS (Agilent Technologies, Santa Monica, CA, USA); reaction mode: on; | Rf power: 1500 W; sampling depth: 7.2 mm; gas flow rates: 0.88 L/min (carrier, Ar), 0.25 L/min (makeup, Ar), 4.7 mL/min (He); integration time: 0.1 s; |
| 98. | ICP-MS | ICP-MS spectrometer (Okamoto, 1997); |  |
| 99. | ICP-MS | ICP-MS spectrometer NexION 300D (Perkin Elmer, Waltham MA); Elemental Scientific Inc (ESI) SC2-DX autosampler (Omaha NE); platinum sampler and skimmer cones (Glass Expansion, West Melbourne, Australia); Microflow PFA-ST nebulizer (ESI); quartz cyclonic spray chamber with baffle (ESI) ; | two modes: 1) dynamic reaction cell mode with ammonia as a reactant gas, 2) kinetic energy discrimination with helium as an inert gas; power: 1600 W; cell gas flow rates: 0.6 mL/min (ammonia), 4.0 mL/min (helium); nebulizer gas flow rate: 0.8 L/min - 1.2 L/min; isotopes: 52-Cr, 51-V, 59-Co; Dwell time: 50 ms; sweeps: 5; |
| 100. | ICP-MS | ICP-MS spectrometer (Elan DRC II PerkinElmer, Norwalk, CT); Meinhard concentric nebulizer (Spectron/Glass Expansion, Ventura, CA, USA); cyclonic spray chamber; | isotopes: 107-Ag, 27-Al, 75-As, 138-Ba, 9-Be, 111-Cd, 59-Co, 53-Cr, 63-Cu, 55-Mn, 98-Mo, 208-Pb, 82-Se, 205-Tl, 238-U, 51-V, 64-Zn; Rf power: 1200 W; data acquisition: 20 sweeps/reading, 1 reading/replicate; dwell time: 50 ms; Ar nebulizer gas flow rate: 0.5 L/min - 0.9 L/min; integration time: 1000 s; |
| 101. | ICP-MS | ICP-MS spectrometer VGElemental PlasmaQuad SOption; |  |
| 102. | ICP-MS | ICP-MS spectrometer (X-SERIES II, Thermo Fisher, USA); interface: Nickel Xt; concentric glass nebulizer; | isotope: 90-Zr; Rf power: 1400 W; gas flow rates: 0.85 L/min (nebulizer), 13.0 L/min (cooling), 0.80 L/min (auxiliary); sampling depth: 150 steps; peristaltic pump speed: 30; 10 sweeps; 3 main runs; dwell time: 30 ms; acquisition mode: peak jumping; |
| 105. | GF-AAS | 4100 ZL atomic absorption spectrometer (Perkin Elmer,  Germany); longitudinal Zeeman background correction; transversely heated graphite atomiser (THGA); end-capped graphite tubes; L’vov platform (PerkinElmer Part No. B3 000653); AS-70 autosampler; Intensitron hollow cathode lamp (Perkin Elmer) | analytical spectral line: 248.3 nm (Fe); slit width: 0.2 nm; lamp current: 30 mA; inert gas: Ar; flow rate: 250 ml/min; sample injection volume: 15 μl; matrix modifier injection volume: 5 μl; measurement mode: integrated absorbance; integration time: 5 s; baseline offset correction: 2 s; operating conditions: 1) dry: 110 °C, ramp 1 s, hold 30 s, 2) dry: 130 °C, ramp 15 s, hold 30 s, 3) pyrolysis: 1400 °C, ramp 10 s, hold 20 s, 4) atomisation: 2100 °C, ramp 0 s, hold 5 s, 5) cleaning: 2450 °C, ramp 1 s, hold 3 s; injection temperature: 20 °C; |
| 108. | GF-AAS | Atomic absorption spectrometer 4110 ZL (Perkin-Elmer); Zeeman-effect background correction; chromium hollow-cathode lamp; transversely heated graphite tubes; Lvov platforms; | analytical spectral line: 357.9 nm (Cr); spectral band pass: 0.7 nm; pure gas: Ar; operating conditions: 1) dry: 110 °C, ramp 10 s, hold 20 s, 2) dry: 130 °C, ramp 15 s, hold 30 s, 3) pyrolysis: 1500 °C, ramp 10 s, hold 20 s, 4) atomisation: 2300 °C, ramp 0 s, hold 5 s, 5) clean: 2600 °C, ramp 1 s, hold 3 s; |
| 110. | GF-AAS  F-AAS | Varian SpectrAA-300Z atomic absorption spectrophotometer (Victoria, Australia); graphite furnace and Zeeman background correction system; autosampler (Varian); Mn and Cu hollow cathode lamps; pyrolytically-coated partition graphite tubes (Varian);  Varian SpectrAA-20 flame atomic absorption spectrophotometer; deuterium background correction; zinc hollow cathode lamp; | analytical spectral lines: 324.8 nm (Cu), 279.5 nm (Mn); slit width: 0.5 nm (Cu), 0.2 nm (Mn); lamp current: 4 mA (Cu), 5 mA (Mn); volume of the injected to the graphite tube: 5 μl; operating conditions for Cu determination: 1) 120 °C, ramp 30 s, hold 10 s, Ar flow rate: 3.0 L/min, 2) 700 °C, ramp 30 s, hold 10 s, Ar flow rate: 3.0 L/min, 3) 2600 °C, ramp 1 s, hold 7 s, Ar flow rate: 0.0 L/min, 4) 40 °C, ramp 13 s, hold 2 s, Ar flow rate: 3.0 L/min; operating conditions for Mn determination: 1) 110 °C, ramp 35 s, hold 15 s, Ar flow rate: 3.0 L/min, 2) 650 °C, ramp 35 s, hold 20 s, Ar flow rate: 3.0 L/min, 3) 2500 °C, ramp 1 s, hold 67 s, Ar flow rate: 0.0 L/min, 4) 40 °C, ramp 10 s, hold 4 s, Ar flow rate: 3.0 L/min;  analytical spectral line: 213.9 nm (Zn); slit width: 1.0 nm (Zn); lamp current: 5 mA (Zn); |
| 118. | ICP-OES | iCAP 6300 duo plasma emission simultaneous spectrometer (Thermo Electron, Waltham, MA); detector: charge-injection device; observation mode: radial/axial (auto view); spray chamber: cyclonic with concentric nebulizer (Meinhard); | Rf power: 1.150 kW; Rf: 27.12 MHz; pump rate: 50 rpm; integration time: 15 s/15 s (low/high wavelength); gas flow rates: 0.5 L/min (auxiliary), 20 L/min (coolant); nebulizer gas pressure: 0.15 MPa; |
| 119. | ICP-MS | ELEMENT 2 (Thermo Fisher Scientific, Bremen, Germany); sampling and skimmer cone: Pt; two sample introduction systems: 1) proposed: HPCN nebulizer (customized concentric type nebulizer body from ONIZUKA Glass/ST JAPAN, Tokyo, Japan, and a tapered liquid capillary made from a fused silica capillary, GL Sciences, Japan), IsoMist spray chamber, loop injection unit (KP-11 model, Ogawa. Co. Ltd., Japan), polypropylene micro syringe; 2) conventional: conical nebulizer (Glass expansion); cyclone chamber at room temperature (i.e. 27 ◦C); | Rf: 27.12 MHz; incident Rf power: 1.5 kW; reflected power: <2W; gas flow rates: 16 L/min (outer gas), 0.9 L/min (intermediate gas), 1.05 L/min (carrier gas for HPCN) and 0.95 L/min (carrier gas for conical nebulizer); sampling depth: -2mm; solution flow rate: 10 μl/min (HPCN), 1 ml/min (conventional); mass resolution: m/Δm 4000; scanning mode: E-scan; integrated mass window: 50%; data points: 20 points/peak; dwell time: 10 ms/point; integration: 5 times; repetition: 5 times; oxide formation ratio YO^+^/Y^+^: 0.5% (HPCN), 1.5% (conventional); m/z: 23-Na^+^, 26-Mg^+^, 31-P^+^, 32-S^+^, 39-K^+^, 44-Ca^+^, 52-Cr^+^, 55-Mn^+^ 56-Fe^+^, 59-Co^+^, 60-Ni^+^, 63-Cu^+^, 66-Zn^+^, 89-Y^+^, 95-Mo^+^, 111-Cd^+^, 137-Ba^+^, 208-Pb^+^; |
